# Supplementary material for: Estimation of stillbirths attributable to ambient fine particles in 137 countries
Source: Nat Commun. 2022 Nov 29;13:6950. doi: 10.1038/s41467-022-34250-4 (PMC9709081; doi:10.1038/s41467-022-34250-4)
Supplement: Supplementary file 1 — Supplementary Information [file 41467_2022_34250_MOESM1_ESM.pdf]

Estimation of Stillbirths Attributable to Ambient Fine Particles in 137 Countries

Tao Xue[1,*,#], PhD, Mingkun Tong[1,#], et al.

Supplementary Figures

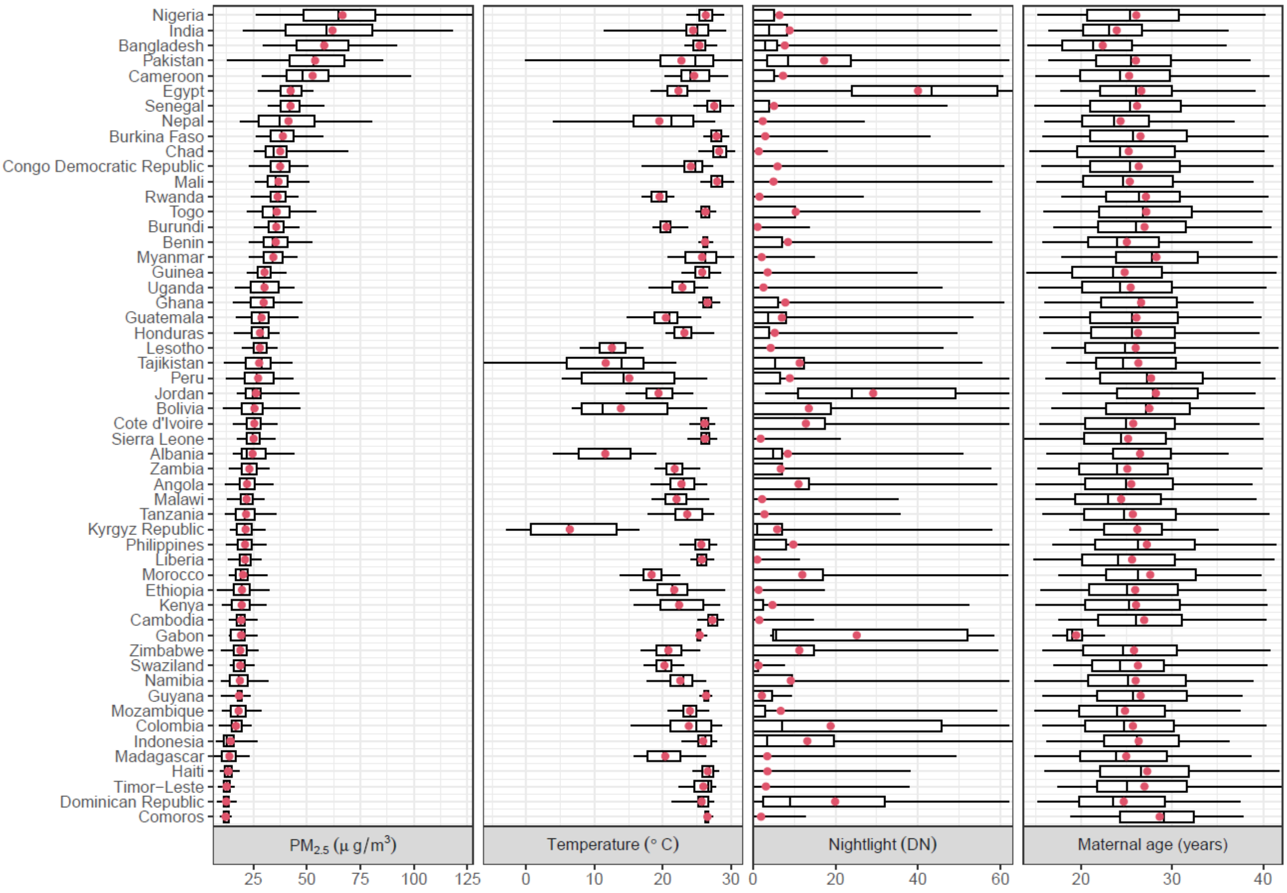

Supplementary Fig. 1 Distributions of continuous covariates among the 13,870 stillbirths and their 32,449 controls, by countries. The centre red dots and black bars are means and medians, respectively. The box bounds and whiskers indicate for ranges from 25th to 75th and from 2.5th to 97.5th percentile, respectively.

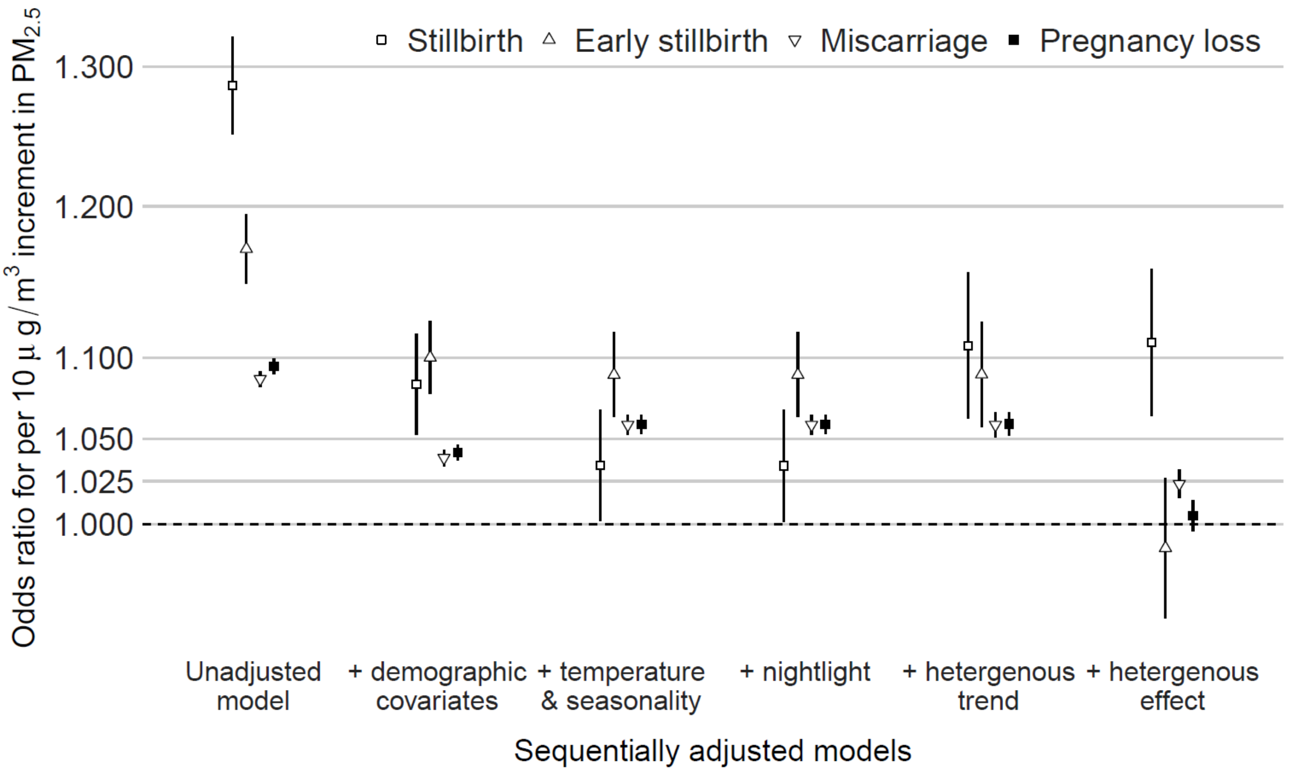

Supplementary Fig. 2 The linear association between $PM_{2.5}$ exposure and stillbirth or secondary outcomes, including early stillbirth, miscarriage and pregnancy loss. The dots are point estimates and the error bars are corresponding 95% confidence intervals.

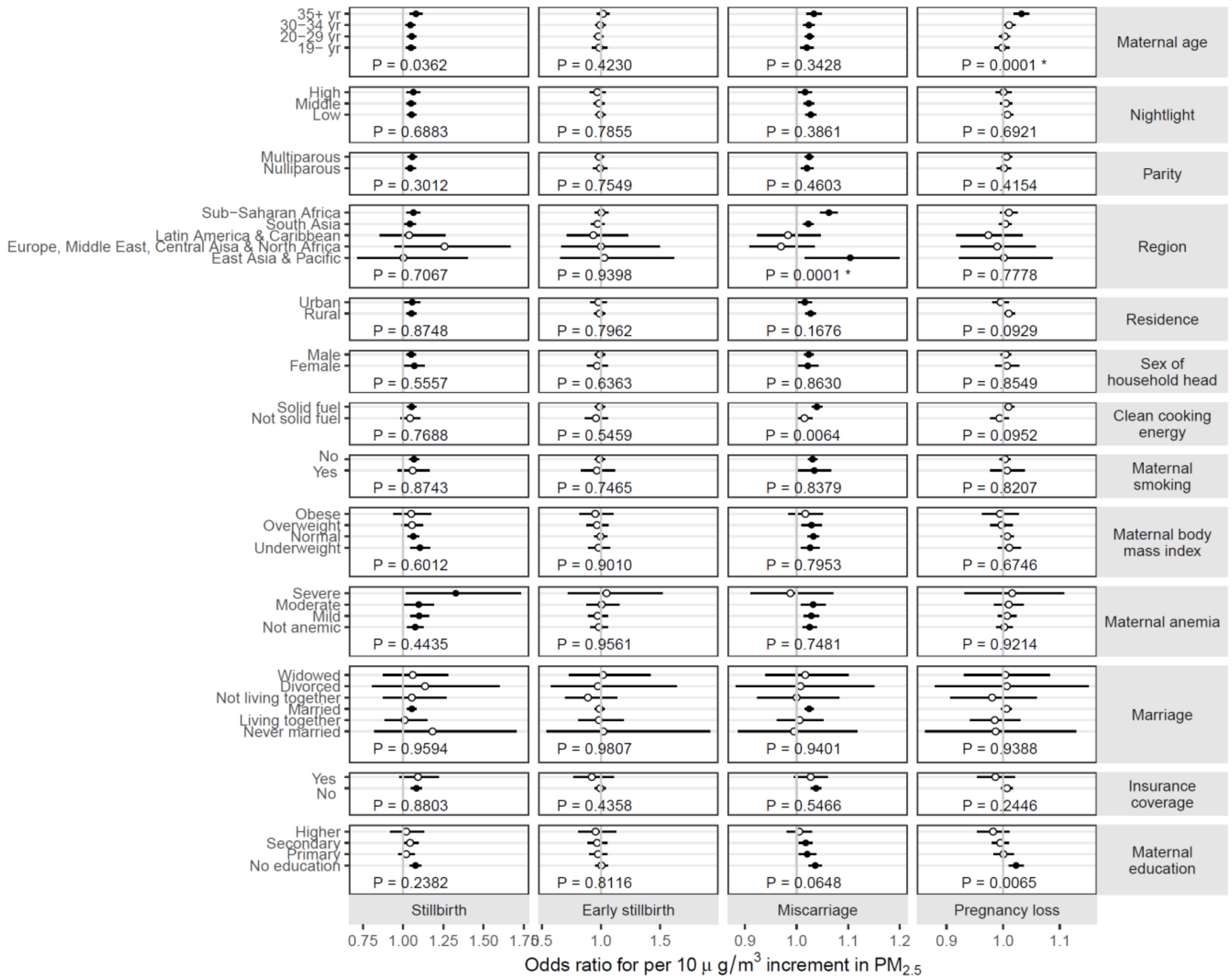

Supplementary Fig. 3 The subpopulation-specific linear associations between $PM_{2.5}$ exposure and stillbirth or secondary outcomes, including early stillbirth, miscarriage and pregnancy loss. The nightlight was classified as low (≤ 4 DN), middle (4 – 20.5 DN), or high (> 20.5 DN) level group. The dots are point estimates and the error bars are corresponding 95% confidence intervals. The original p-values for Wald tests on interaction effects are shown in each panel. The stars (*) indicate for statistically significant differences after Bonferroni corrections for multiple comparisons.

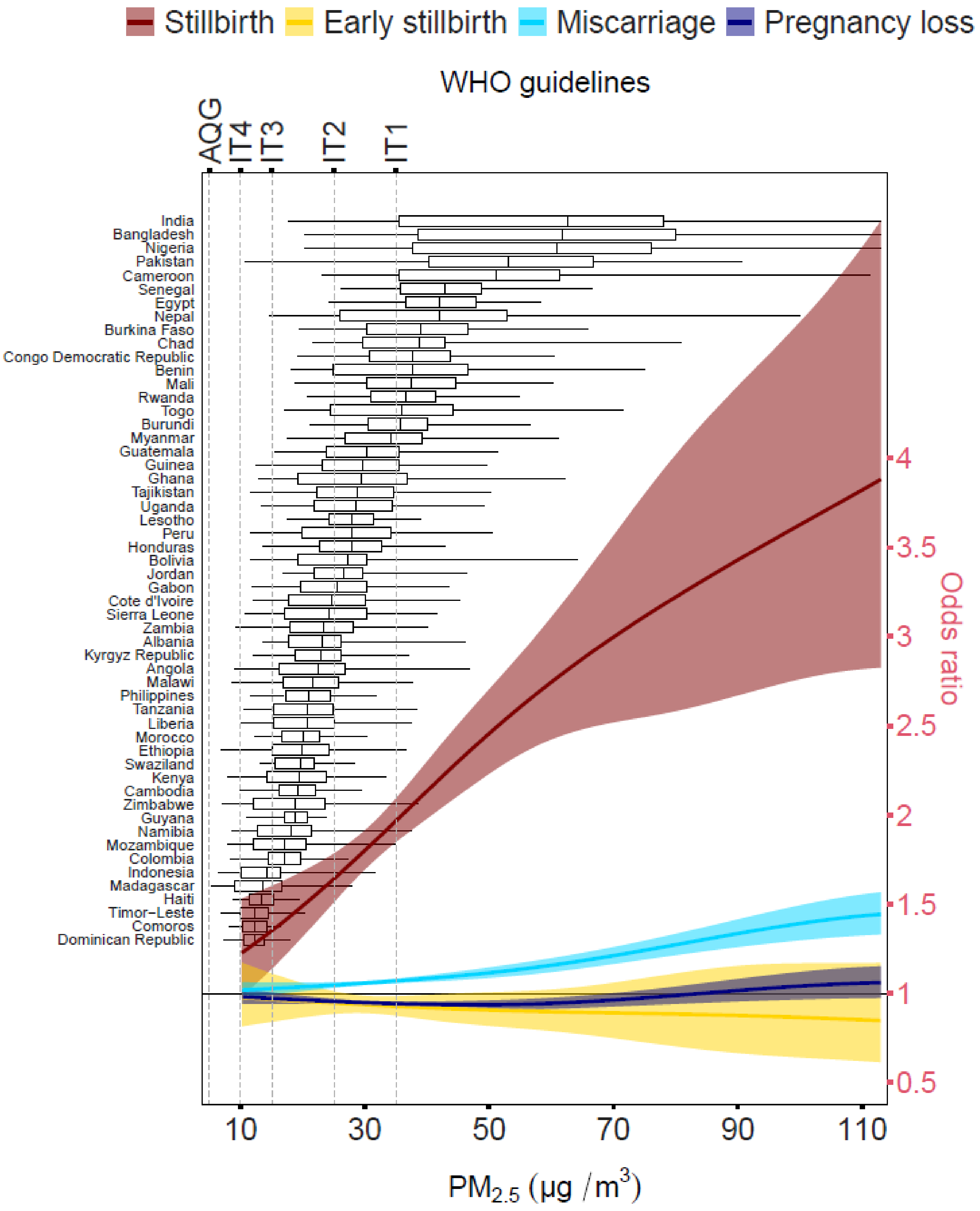

Supplementary Fig. 4 The nonlinear association between $PM_{2.5}$ and stillbirth, early stillbirth, miscarriage or pregnancy loss for all maternal ages. The corresponding $PM_{2.5}$ exposures for pregnancy loss in those countries are shown by the boxplots (The centre bars are medians; the box bounds and whiskers indicate for ranges from 25th to 75th and from 2.5^{th} to 97.5^{th} percentile, respectively.). The colored ribbons are the pointwise 95% confidence intervals.

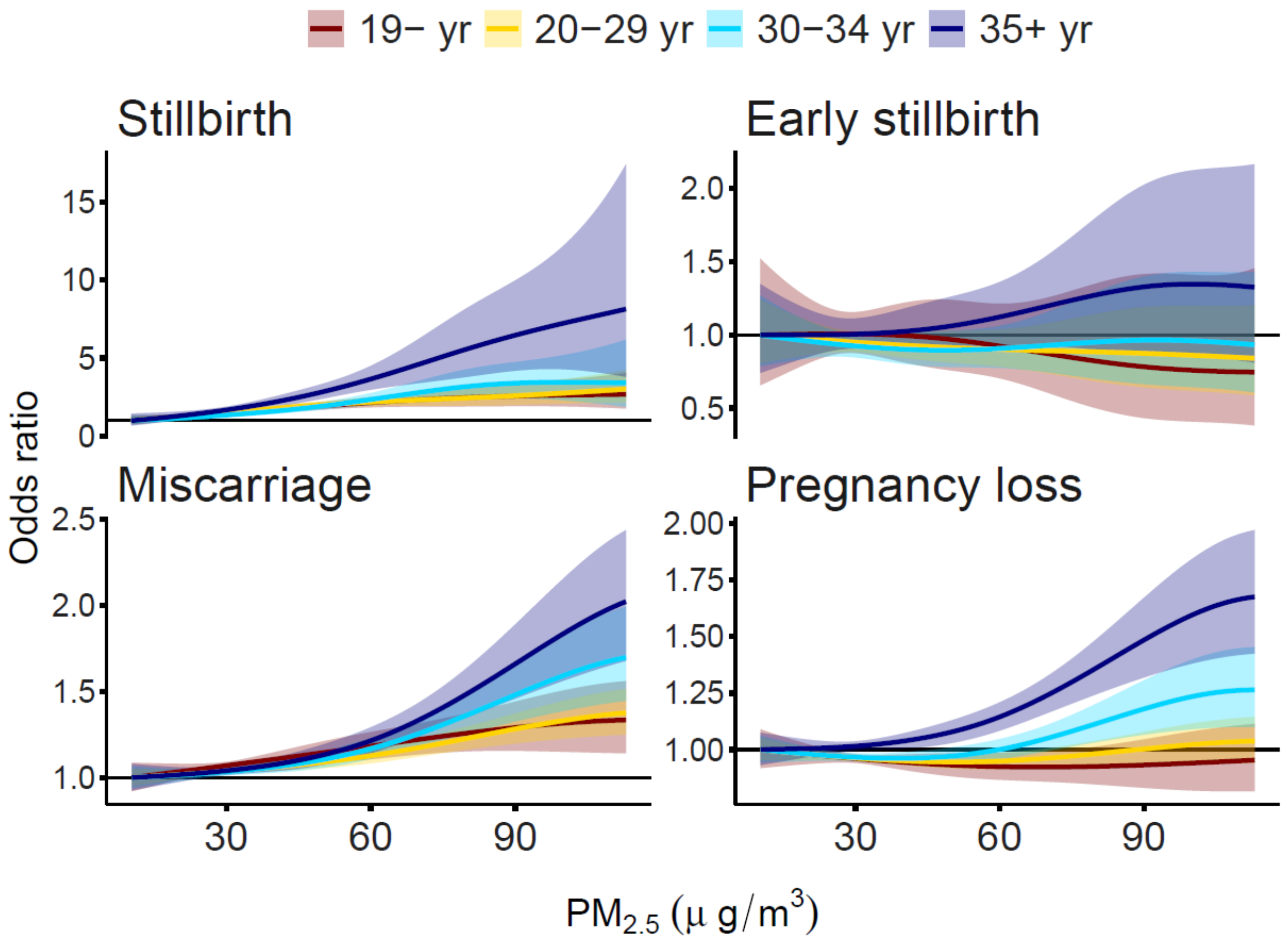

Supplementary Fig. 5 The nonlinear association between $PM_{2.5}$ and stillbirth, early stillbirth, miscarriage or pregnancy loss, specifically for four maternal age groups. The corresponding $PM_{2.5}$ exposures for pregnancy loss in those countries are shown by the boxplots. The colored ribbons are the pointwise 95% confidence intervals.

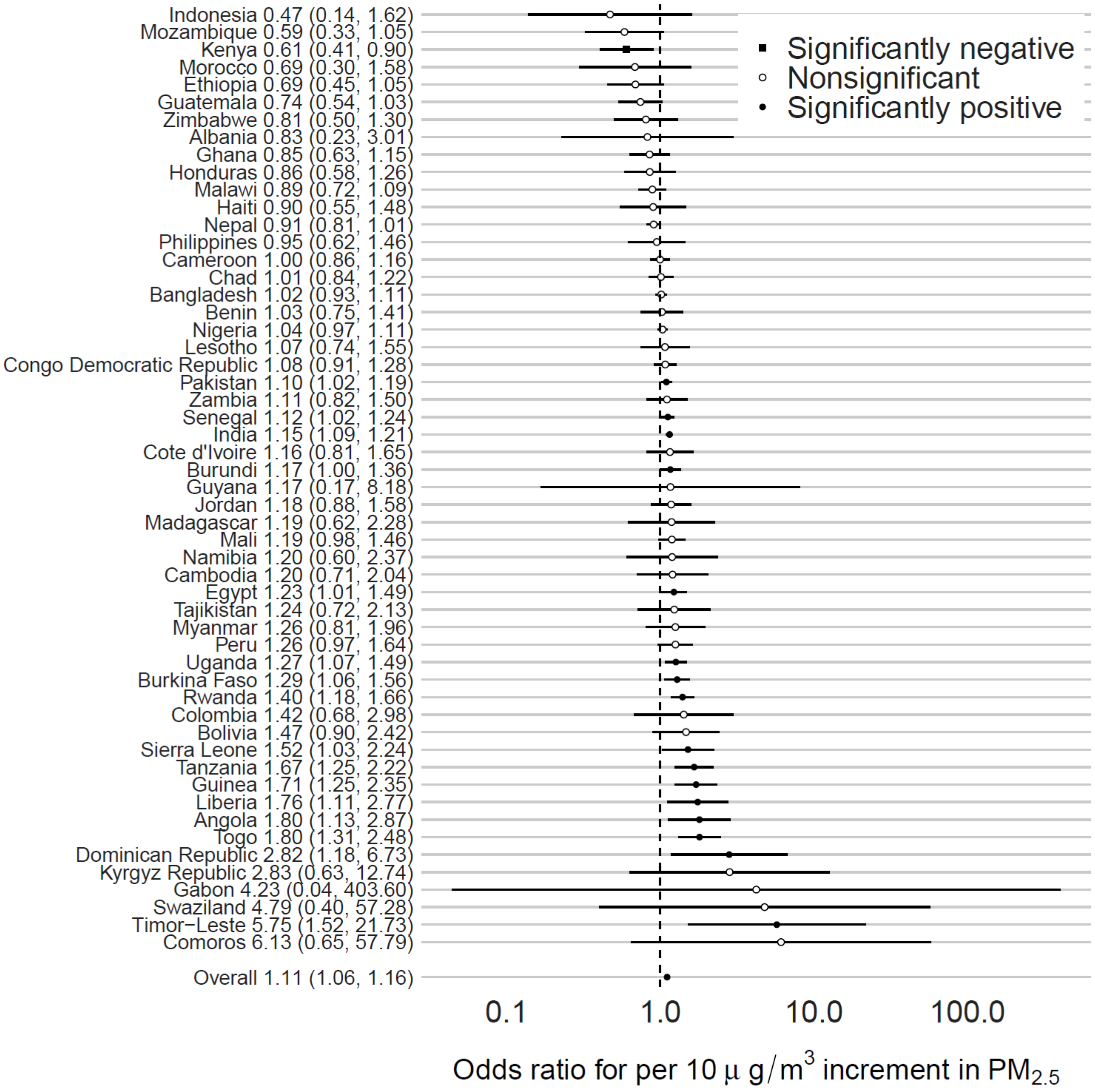

Supplementary Fig. 6 The linear association between $PM_{2.5}$ and stillbirth, estimated by countries. The dots are point estimates, and the error bars are corresponding 95% confidence intervals.

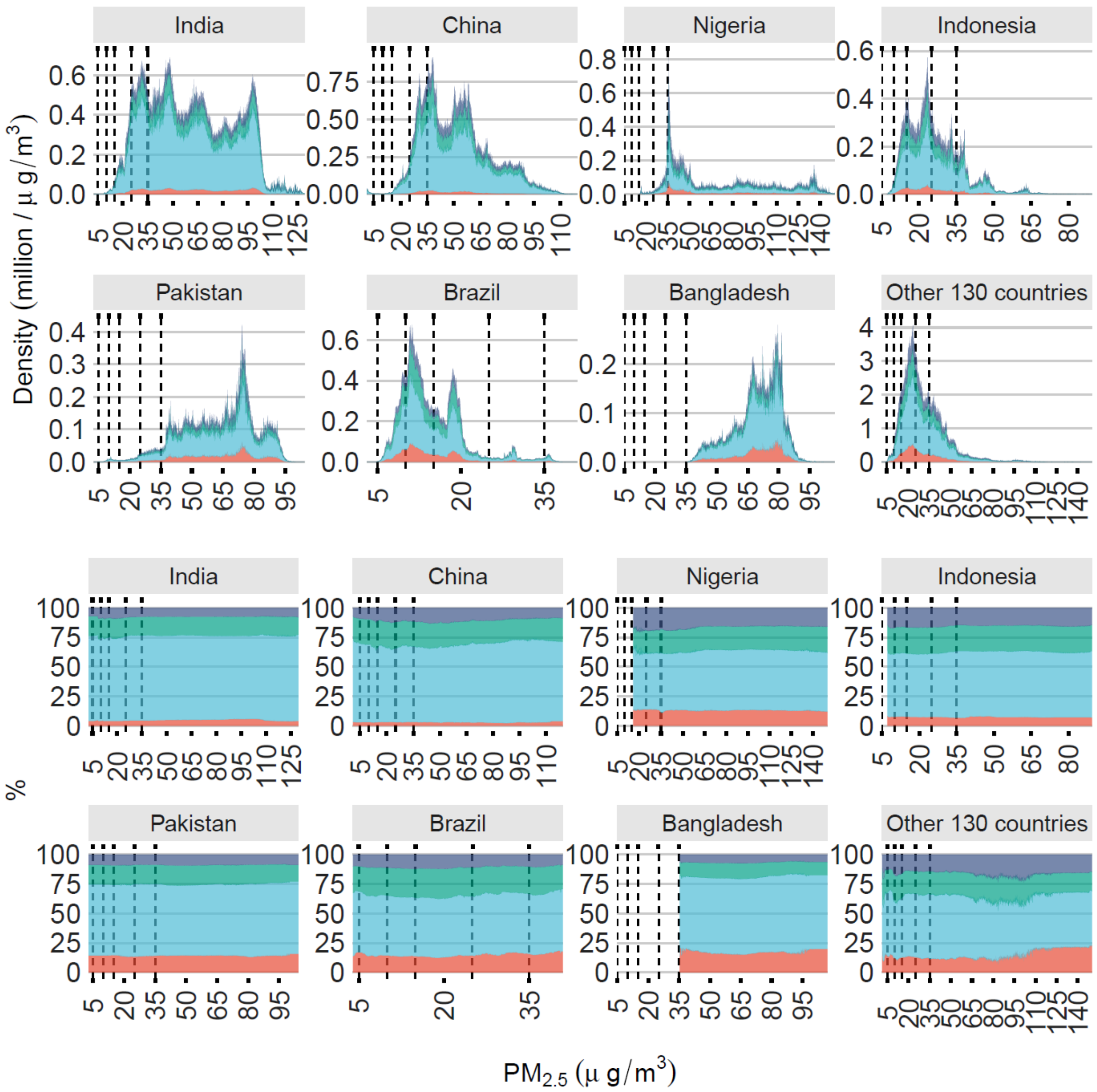

Supplementary Fig. 7 Distributions (top) and relative fractions (bottom) of age-specific populations at risk (i.e., pregnancies stratified by maternal ages) by different PM$_{2.5}$ exposure levels among each of the seven countries with the largest number of pregnancies.

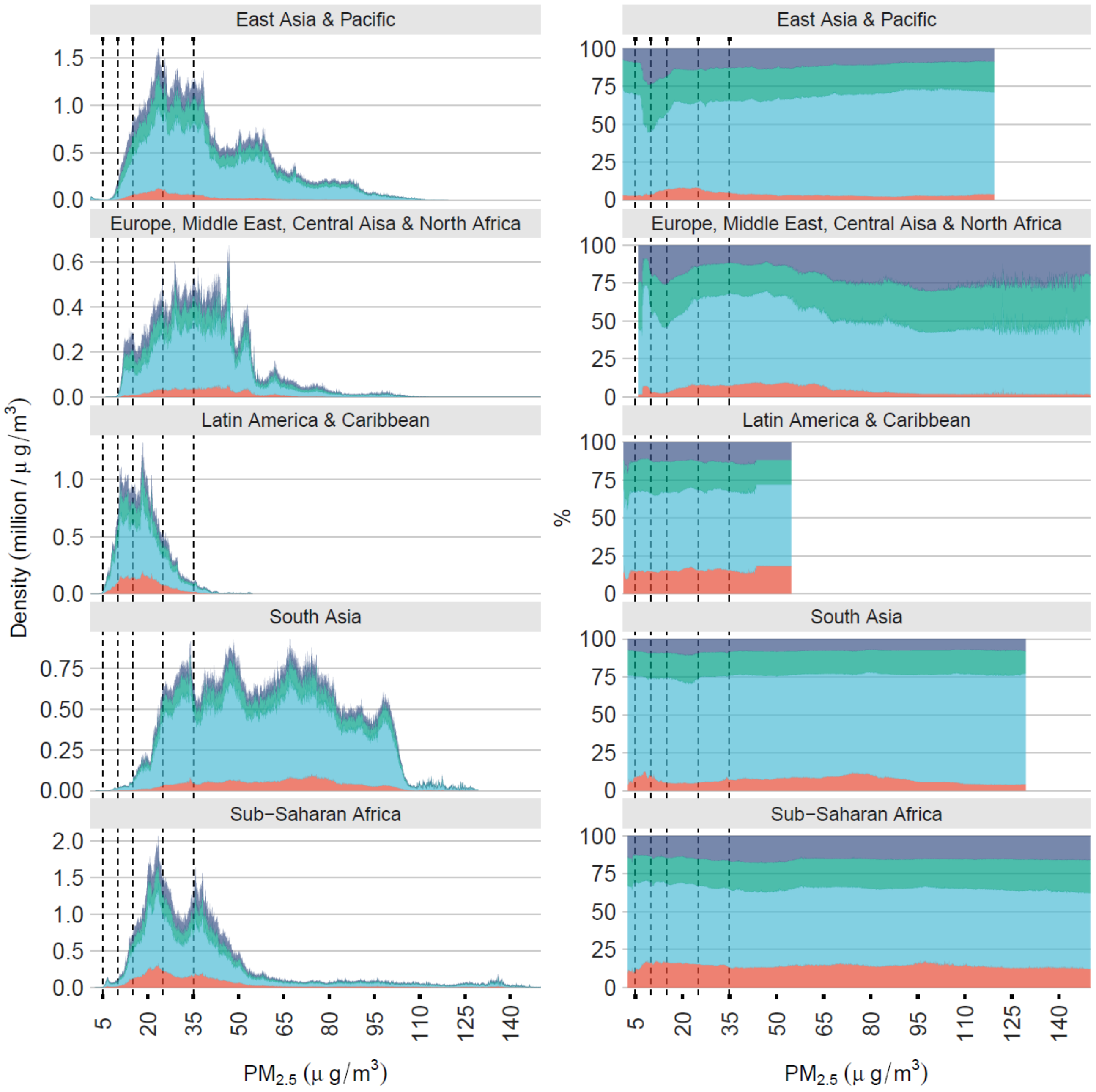

Supplementary Fig. 8 Distributions (left) and relative fractions (right) of age-specific populations at risk (i.e., pregnancies stratified by maternal ages) by different $PM_{2.5}$ exposure levels among each of the five studied regions.

(a)

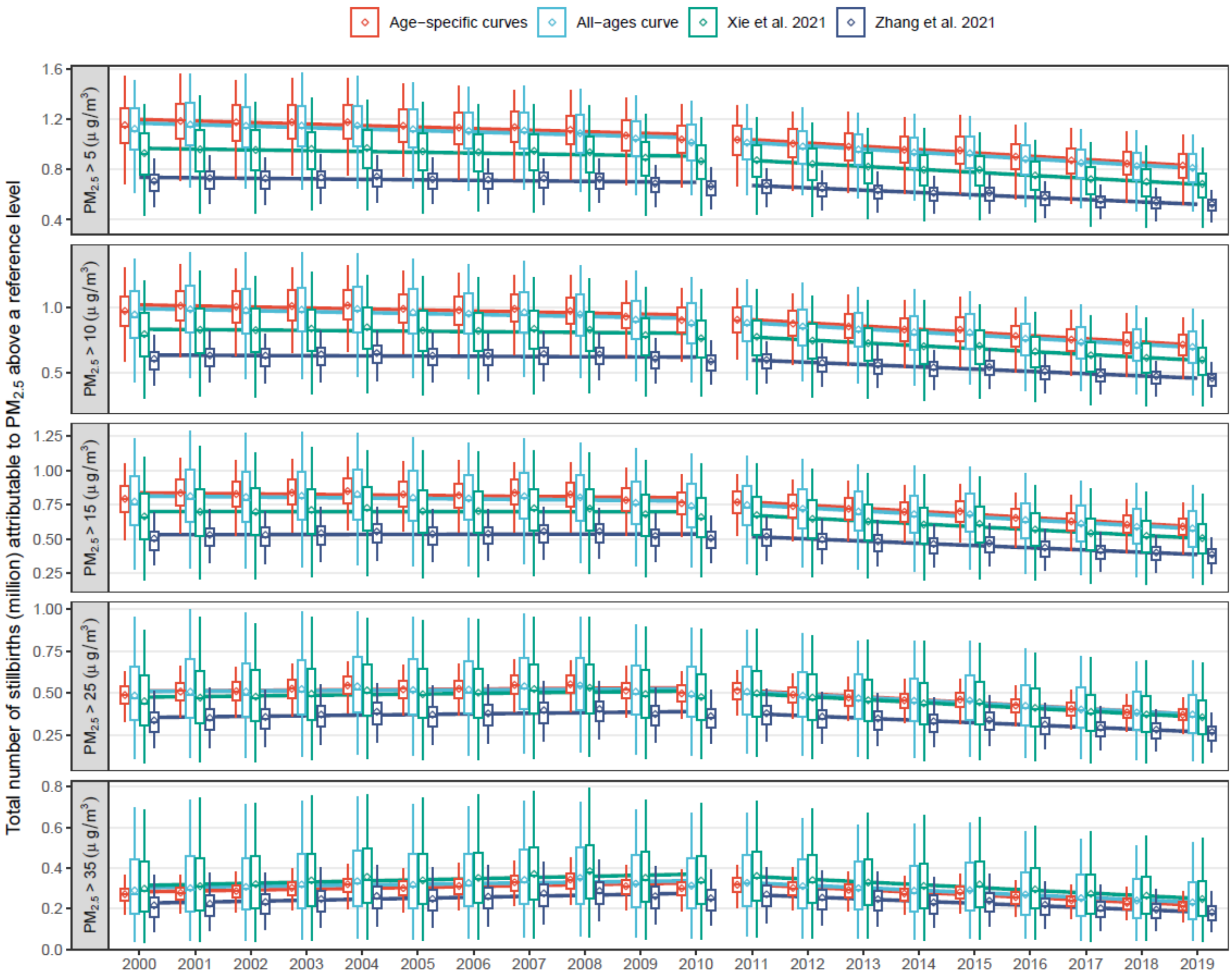

Age−specific curves
All−ages curve
Xie et al. 2021
Zhang et al. 2021
Total number of stillbirths (million) attributable to PM2.5 above a reference level
PM2.5 > 5 (μ g/m3)
PM2.5 > 10 (μ g/m3)
PM2.5 > 15 (μ g/m3)
PM2.5 > 25 (μ g/m3)
PM2.5 > 35 (μ g/m3)
1.6
1.2
0.8
0.4
1.0
0.5
1.25
1.00
0.75
0.50
0.25
0.8
0.6
0.4
0.2
0.0
2000
2001
2002
2003
2004
2005
2006
2007
2008
2009
2010
2011
2012
2013
2014
2015
2016
2017
2018
2019

(b)

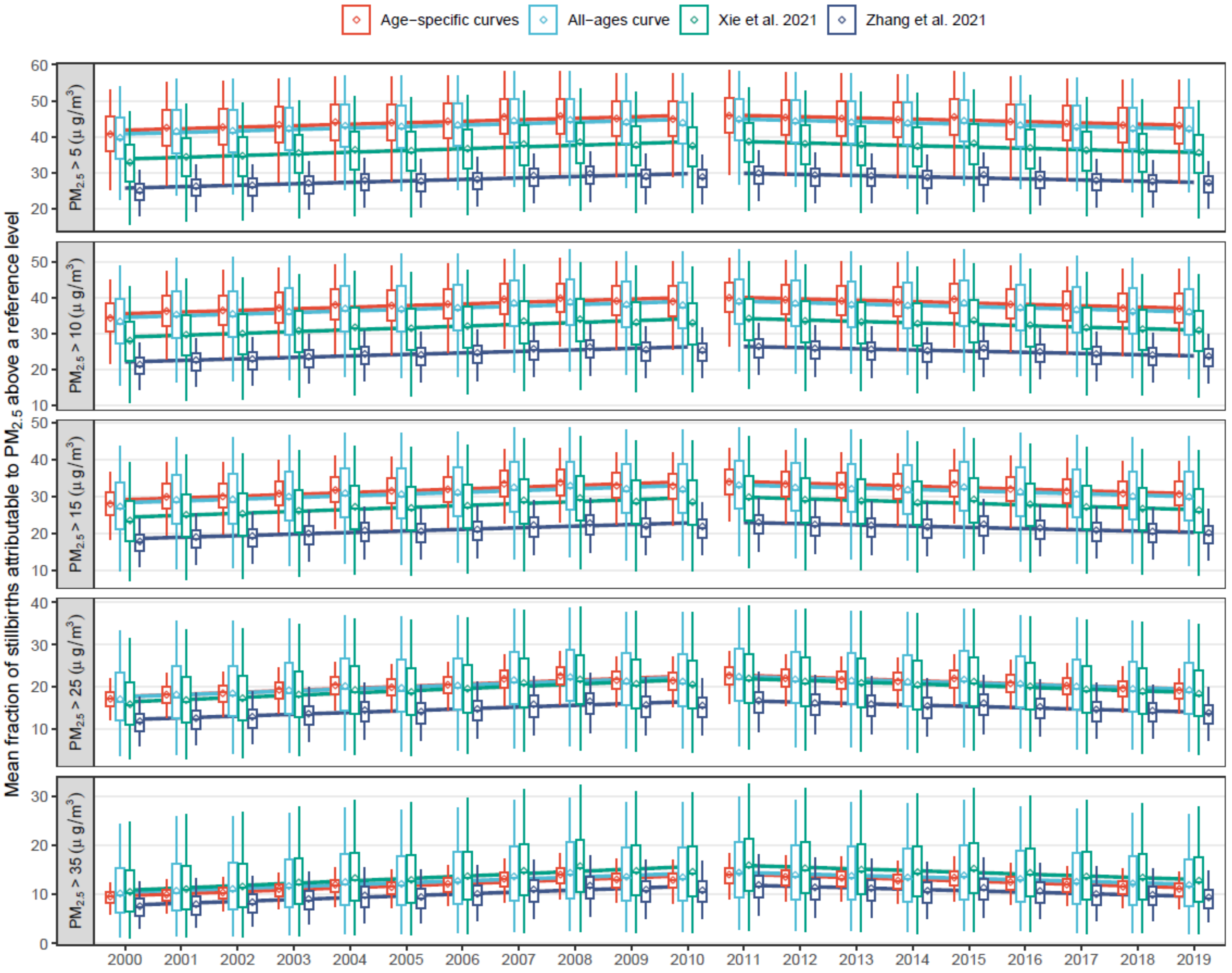

Supplementary Fig. 9 The total number (a) or average fraction (b) of stillbirths attributable to $PM_{2.5}$ exposure from 2000 to 2019 for the 137 countries, estimated by different exposure-response curves. The boxplots show the distributions of the Monte Carlo simulations. The centre diamond dots and bars are means and medians, respectively. The box bounds and whiskers indicate for ranges from 25th to 75th and from 2.5^{th} to 97.5^{th} percentile, respectively.

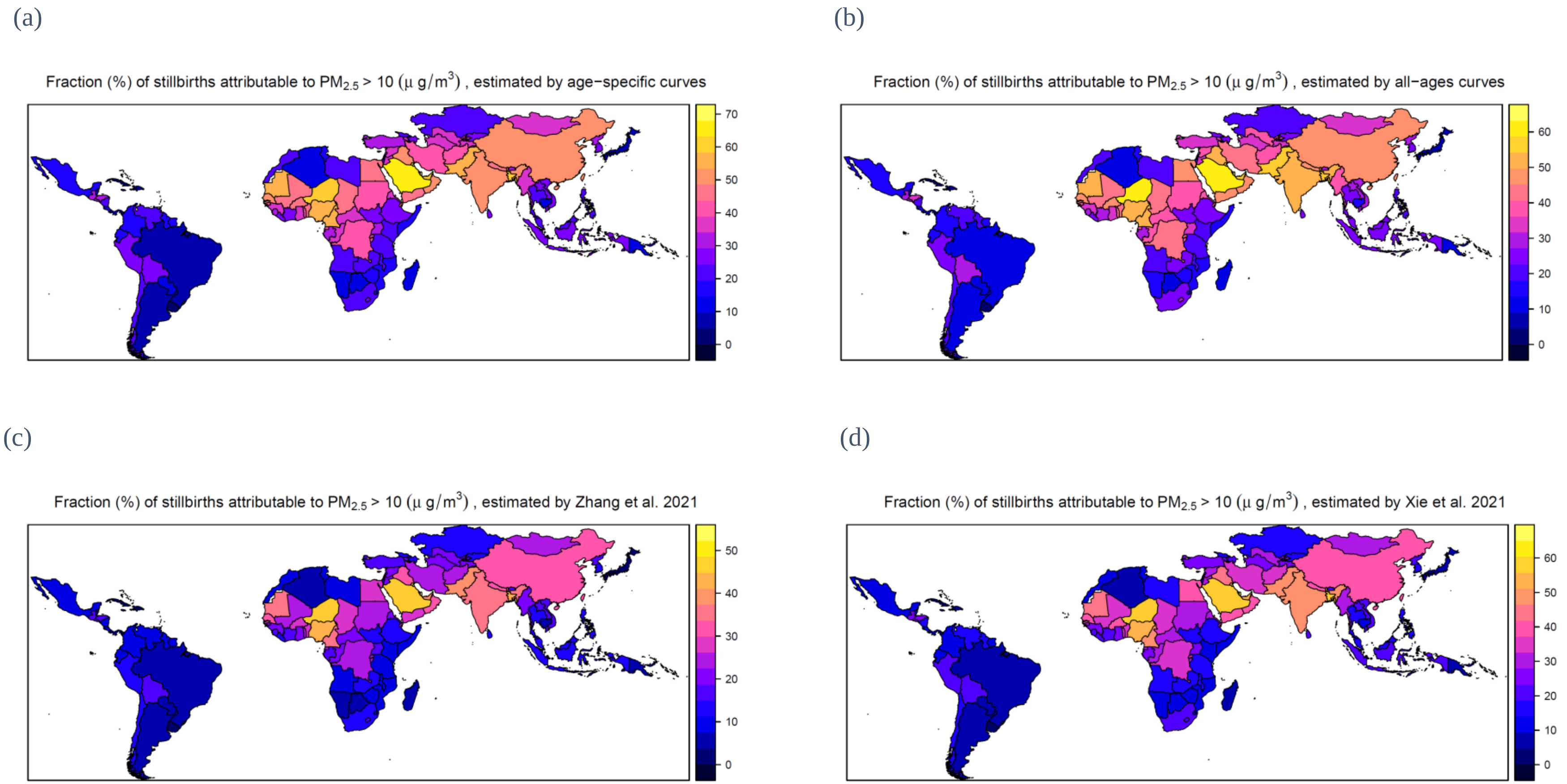

Supplementary Fig. 10 The spatial distribution of $PM_{2.5}$-related stillbirths, estimated by different exposure-response curves.

Supplementary Tables

Supplementary Table 1 List of the 137 countries included into our assessment.

ISO	NAME
AFG	Afghanistan
AGO	Angola
ARE	United Arab Emirates
ARG	Argentina
ARM	Armenia
ATG	Antigua and Barb.
AZE	Azerbaijan
BDI	Burundi
BEN	Benin
BFA	Burkina Faso
BGD	Bangladesh
BHR	Bahrain
BHS	Bahamas
BLZ	Belize
BOL	Bolivia
BRA	Brazil
BRB	Barbados
BRN	Brunei
BTN	Bhutan
BWA	Botswana
CAF	Central African Rep.
CHL	Chile
CHN	China
CIV	Cote d'Ivoire
CMR	Cameroon
COD	Dem. Rep. Congo
COG	Congo
COL	Colombia
COM	Comoros
CPV	Cabo Verde
CRI	Costa Rica
CUB	Cuba
CYP	Cyprus
DJI	Djibouti
DMA	Dominica
DOM	Dominican Rep.
DZA	Algeria
ECU	Ecuador
EGY	Egypt
ERI	Eritrea
ETH	Ethiopia
GAB	Gabon
GEO	Georgia
GHA	Ghana
GIN	Guinea
GMB	Gambia

ISO	NAME
GNB	Guinea-Bissau
GNQ	Eq. Guinea
GRD	Grenada
GTM	Guatemala
GUY	Guyana
HND	Honduras
HTI	Haiti
IDN	Indonesia
IND	India
IRN	Iran
IRQ	Iraq
ISR	Israel
JAM	Jamaica
JOR	Jordan
JPN	Japan
KAZ	Kazakhstan
KEN	Kenya
KGZ	Kyrgyzstan
KHM	Cambodia
KNA	St. Kitts and Nevis
KOR	South Korea
KWT	Kuwait
LAO	Laos
LBN	Lebanon
LBR	Liberia
LBY	Libya
LCA	Saint Lucia
LKA	Sri Lanka
LSO	Lesotho
MAR	Morocco
MDG	Madagascar
MDV	Maldives
MEX	Mexico
MLI	Mali
MMR	Myanmar
MNG	Mongolia
MOZ	Mozambique
MRT	Mauritania
MUS	Mauritius
MWI	Malawi
MYS	Malaysia
NAM	Namibia
NER	Niger
NGA	Nigeria
NIC	Nicaragua
NPL	Nepal

ISO	NAME
OMN	Oman
PAK	Pakistan
PAN	Panama
PER	Peru
PHL	Philippines
PNG	Papua New Guinea
PRK	North Korea
PRY	Paraguay
PSE	Palestine
QAT	Qatar
RWA	Rwanda
SAU	Saudi Arabia
SDN	Sudan
SEN	Senegal
SGP	Singapore
SLB	Solomon Is.
SLE	Sierra Leone
SLV	El Salvador
SOM	Somalia
SSD	S. Sudan
STP	São Tomé and Príncipe
SUR	Suriname
SWZ	eSwatini
SYC	Seychelles
SYR	Syria
TCD	Chad
TGO	Togo
THA	Thailand
TJK	Tajikistan
TKM	Turkmenistan
TLS	Timor-Leste
TTO	Trinidad and Tobago
TUN	Tunisia
TUR	Turkey
TZA	Tanzania
UGA	Uganda
URY	Uruguay
UZB	Uzbekistan
VCT	St. Vin. and Gren.
VEN	Venezuela
VNM	Vietnam
YEM	Yemen
ZAF	South Africa
ZMB	Zambia
ZWE	Zimbabwe

Supplementary Table 2 Summary of the constant variables of the analyzed mothers in the epidemiological study to establish the exposure-response curves between $PM_{2.5}$ and stillbirth or other similar outcomes.

Variable	Group	Stillbirth	Early stillbirth	Miscarriage	Pregnancy loss
		N (percentage)			
Total		13,870(100%)	9,783(100%)	85,548(100%)	109,201(100%)
Region	East Asia & Pacific	487(3.5%)	499(5.1%)	7,157(8.4%)	8,143(7.5%)
	Europe, Middle East, Central Asia & North Africa	532(3.8%)	635(6.5%)	10,254(12.0%)	11,421(10.5%)
	Latin America & Caribbean	1,064(7.7%)	958(9.8%)	10,237(12.0%)	12,259(11.2%)
	South Asia	4,755(34.3%)	2,875(29.4%)	31,227(36.5%)	38,857(35.6%)
	Sub-Saharan Africa	7,032(50.7%)	4,816(49.2%)	26,673(31.2%)	38,521(35.3%)
Residence	Rural	10,139(73.1%)	6,489(66.3%)	50,018(58.5%)	66,646(61.0%)
	Urban	3,731(26.9%)	3,294(33.7%)	35,530(41.5%)	42,555(39.0%)
Sex of household head	Female	2,225(16.0%)	1,782(18.2%)	14,681(17.2%)	18,688(17.1%)
	Male	11,645(84.0%)	8,001(81.8%)	70,867(82.8%)	90,513(82.9%)
Type of cooking energy	Not solid fuel	2,219(16.0%)	2,237(22.9%)	29,817(34.9%)	34,273(31.4%)
	Solid fuel	10,947(78.9%)	6,939(70.9%)	49,592(58.0%)	67,478(61.8%)
	Unknown	704(5.1%)	607(6.2%)	6,139(7.2%)	7,450(6.8%)
Smoking	Yes	806(5.8%)	636(6.5%)	6,045(7.1%)	7,487(6.9%)
	No	11,199(80.7%)	8,008(81.9%)	68,892(80.5%)	88,099(80.7%)
	Unknown	1,865(13.4%)	1,139(11.6%)	10,611(12.4%)	13,615(12.5%)
Maternal body mass index	Underweight	1,339(9.7%)	918(9.4%)	7,319(8.6%)	9,576(8.8%)
	Normal	5,878(42.4%)	4,063(41.5%)	35,304(41.3%)	45,245(41.4%)
	Overweight	1,638(11.8%)	1,300(13.3%)	14,006(16.4%)	16,944(15.5%)
	Obese	753(5.4%)	628(6.4%)	6,612(7.7%)	7,993(7.3%)
	Unknown	4,262(30.7%)	2,874(29.4%)	22,307(26.1%)	29,443(27.0%)
Maternal anemia	Mild	2,218(16.0%)	1,477(15.1%)	15,171(17.7%)	18,866(17.3%)
	Moderate	843(6.1%)	556(5.7%)	4,913(5.7%)	6,312(5.8%)
	Not anemic	3,396(24.5%)	2,470(25.2%)	25,434(29.7%)	31,300(28.7%)
	Severe	97(0.7%)	57(0.6%)	379(0.4%)	533(0.5%)
	Unknown	7,316(52.7%)	5,223(53.4%)	39,651(46.3%)	52,190(47.8%)
Marriage	Divorced	173(1.2%)	153(1.6%)	1,091(1.3%)	1,417(1.3%)
	Living together	1,380(9.9%)	1,230(12.6%)	9,826(11.5%)	12,436(11.4%)
	Married	11,575(83.5%)	7,701(78.7%)	69,914(81.7%)	89,190(81.7%)
	Never married	179(1.3%)	204(2.1%)	1,345(1.6%)	1,728(1.6%)
	Not living together	386(2.8%)	362(3.7%)	2,542(3.0%)	3,290(3.0%)
	Widowed	177(1.3%)	133(1.4%)	829(1.0%)	1,139(1.0%)
	Unknown			1(0.0%)	1(0.0%)
Insurance coverage	No	8,034(57.9%)	5,693(58.2%)	47,267(55.3%)	60,994(55.9%)
	Yes	796(5.7%)	752(7.7%)	9,047(10.6%)	10,595(9.7%)
	Unknown	5,040(36.3%)	3,338(34.1%)	29,234(34.2%)	37,612(34.4%)
	Higher	557(4.0%)	625(6.4%)	9,580(11.2%)	10,762(9.9%)

Maternal education	No education	5,504(39.7%)	3,216(32.9%)	20,415(23.9%)	29,135(26.7%)
	Primary	4,548(32.8%)	3,093(31.6%)	22,786(26.6%)	30,427(27.9%)
	Secondary	3,261(23.5%)	2,849(29.1%)	32,766(38.3%)	38,876(35.6%)
	Unknown			1(0.0%)	1(0.0%)
		Mean (Standard Deviation, Interquartile Range)			
Time intervals between case and controls (years)		3.81 (2.45, 1.96 ~ 5.14)	3.86 (2.49, 1.96 ~ 5.25)	3.92 (2.69, 1.83 ~ 5.42)	3.90 (2.65, 1.88 ~ 5.36)

Supplementary Table 3 Summary of exposure to $PM_{2.5}$ among the 137 studied countries for the population at risk of stillbirth.

ISO	Age group	$PM_{2.5}$ average ($\mu g/m^3$)	Percentage of pregnancies exposed to $PM_{2.5}$ > C_0 $\mu g/m^3$				
			C_0 = 5	C_0 = 10	C_0 = 15	C_0 = 25	C_0 = 35
ALL	All ages	43.24	99.96	98.87	93.51	73.16	53.69
ALL	< 20 yr	40.09	99.98	98.4	91.77	65.85	46.45
ALL	20-29 yr	44.8	99.96	99	94.43	75.73	56.54
ALL	30-34 yr	41.84	99.96	98.73	92.46	71.21	51.39
ALL	> 34 yr	40.36	99.97	98.86	92.08	69.42	49.11
AFG	All ages	39.31	100	99.97	99.82	94.29	55.59
AFG	< 20 yr	38.97	100	99.97	99.84	94.67	53.53
AFG	20-29 yr	39.35	100	99.97	99.83	94.35	55.64
AFG	30-34 yr	39.67	100	99.97	99.82	94.24	57.99
AFG	> 34 yr	39.08	100	99.97	99.8	93.71	54.8
AGO	All ages	21.59	100	99.71	88.54	21.31	2.46
AGO	< 20 yr	21.36	100	99.71	87.92	19.73	2.19
AGO	20-29 yr	21.51	100	99.7	88.17	21.1	2.36
AGO	30-34 yr	21.84	100	99.73	89.76	22.53	2.66
AGO	> 34 yr	21.79	100	99.75	89.06	22.35	2.82
ARE	All ages	62.29	100	100	100	100	100
ARE	< 20 yr	62.29	100	100	100	100	100
ARE	20-29 yr	62.29	100	100	100	100	100
ARE	30-34 yr	62.29	100	100	100	100	100
ARE	> 34 yr	62.29	100	100	100	100	100
ARG	All ages	14.4	100	95.92	35.25	0.78	0
ARG	< 20 yr	14.49	100	95.89	36.81	0.78	0
ARG	20-29 yr	14.39	100	95.92	35.23	0.74	0
ARG	30-34 yr	14.41	100	95.99	35.06	0.83	0
ARG	> 34 yr	14.33	100	95.86	34.24	0.86	0
ARM	All ages	32.36	100	100	100	92.16	43.94
ARM	< 20 yr	32.18	100	100	100	91.69	42.48
ARM	20-29 yr	32.37	100	100	100	92.23	43.92
ARM	30-34 yr	32.42	100	100	100	92.09	44.69
ARM	> 34 yr	32.33	100	100	100	91.92	43.9
ATG	All ages	13.37	100	99.69	0.26	0	0

ISO	Age group	$PM_{2.5}$ average ($\mu g/m^3$)	Percentage of pregnancies exposed to $PM_{2.5} > C_0$ $\mu g/m^3$				
			$C_0 = 5$	$C_0 = 10$	$C_0 = 15$	$C_0 = 25$	$C_0 = 35$
ATG	< 20 yr	13.37	100	99.69	0.26	0	0
ATG	20-29 yr	13.37	100	99.69	0.26	0	0
ATG	30-34 yr	13.37	100	99.69	0.26	0	0
ATG	> 34 yr	13.37	100	99.69	0.26	0	0
AZE	All ages	25.74	100	100	100	57.7	0
AZE	< 20 yr	25.78	100	100	100	58.91	0
AZE	20-29 yr	25.74	100	100	100	57.56	0
AZE	30-34 yr	25.7	100	100	100	57.01	0
AZE	> 34 yr	25.75	100	100	100	57.96	0
BDI	All ages	37.14	100	100	100	100	74.78
BDI	< 20 yr	37.14	100	100	100	100	74.85
BDI	20-29 yr	37.15	100	100	100	100	74.83
BDI	30-34 yr	37.13	100	100	100	100	74.7
BDI	> 34 yr	37.12	100	100	100	100	74.66
BEN	All ages	43.04	100	100	100	100	69.47
BEN	< 20 yr	43.51	100	100	100	100	71.25
BEN	20-29 yr	43.18	100	100	100	100	69.54
BEN	30-34 yr	42.91	100	100	100	100	69.09
BEN	> 34 yr	42.18	100	100	100	100	67.87
BFA	All ages	44.88	100	100	100	100	88.76
BFA	< 20 yr	44.86	100	100	100	100	88.59
BFA	20-29 yr	44.89	100	100	100	100	88.55
BFA	30-34 yr	44.92	100	100	100	100	89.11
BFA	> 34 yr	44.84	100	100	100	100	89.25
BGD	All ages	69.58	100	100	100	100	100
BGD	< 20 yr	69.74	100	100	100	100	100
BGD	20-29 yr	69.66	100	100	100	100	100
BGD	30-34 yr	69.21	100	100	100	100	100
BGD	> 34 yr	69.13	100	100	100	100	100
BHR	All ages	56.58	100	100	100	100	100
BHR	< 20 yr	56.58	100	100	100	100	100
BHR	20-29 yr	56.58	100	100	100	100	100
BHR	30-34 yr	56.58	100	100	100	100	100
BHR	> 34 yr	56.58	100	100	100	100	100
BHS	All ages	5.58	82.9	0.1	0	0	0
BHS	< 20 yr	5.59	82.92	0.12	0	0	0
BHS	20-29 yr	5.57	82.89	0.08	0	0	0
BHS	30-34 yr	5.58	82.89	0.11	0	0	0
BHS	> 34 yr	5.58	82.91	0.12	0	0	0
BLZ	All ages	17.3	100	99.99	81.34	0.13	0
BLZ	< 20 yr	17.4	100	99.99	81.42	0.14	0
BLZ	20-29 yr	17.3	100	99.99	81.42	0.13	0

ISO	Age group	$PM_{2.5}$ average ($\mu g/m^3$)	Percentage of pregnancies exposed to $PM_{2.5} > C_0\ \mu g/m^3$				
			$C_0 = 5$	$C_0 = 10$	$C_0 = 15$	$C_0 = 25$	$C_0 = 35$
BLZ	30-34 yr	17.26	100	99.99	81.29	0.12	0
BLZ	> 34 yr	17.22	100	99.99	80.79	0.11	0
BOL	All ages	27.47	100	99.94	99.63	59.43	12.51
BOL	< 20 yr	27.53	100	99.94	99.63	59.83	12.93
BOL	20-29 yr	27.54	100	99.94	99.64	60	12.66
BOL	30-34 yr	27.39	100	99.94	99.62	58.78	12.23
BOL	> 34 yr	27.3	100	99.93	99.61	58.13	12.03
BRA	All ages	14.67	99.99	81.97	39.37	5.55	0.92
BRA	< 20 yr	14.65	99.98	81.22	38.26	6.17	1.01
BRA	20-29 yr	14.67	99.99	81.76	39.27	5.65	0.93
BRA	30-34 yr	14.7	99.99	82.47	40	5.31	0.9
BRA	> 34 yr	14.65	99.99	82.76	39.75	4.89	0.84
BRB	All ages	16.09	100	100	76.88	0	0
BRB	< 20 yr	16.1	100	100	77.32	0	0
BRB	20-29 yr	16.1	100	100	77.45	0	0
BRB	30-34 yr	16.06	100	100	76	0	0
BRB	> 34 yr	16.07	100	100	75.99	0	0
BRN	All ages	12.35	100	100	0	0	0
BRN	< 20 yr	12.35	100	100	0	0	0
BRN	20-29 yr	12.35	100	100	0	0	0
BRN	30-34 yr	12.35	100	100	0	0	0
BRN	> 34 yr	12.35	100	100	0	0	0
BTN	All ages	28.85	100	99.94	96.51	51.91	22.95
BTN	< 20 yr	28.37	100	99.93	96.34	49.6	21.95
BTN	20-29 yr	28.99	100	99.94	96.6	52.67	23.16
BTN	30-34 yr	28.72	100	99.93	96.43	51.46	22.86
BTN	> 34 yr	28.64	100	99.93	96.28	50.3	22.78
BWA	All ages	17.71	100	100	80.07	7.68	0.07
BWA	< 20 yr	17.73	100	100	78.4	8.19	0.08
BWA	20-29 yr	17.71	100	100	80.48	7.65	0.06
BWA	30-34 yr	17.7	100	100	80.31	7.52	0.07
BWA	> 34 yr	17.69	100	100	79.49	7.57	0.07
CAF	All ages	34.9	100	100	100	100	49.52
CAF	< 20 yr	34.91	100	100	100	100	49.88
CAF	20-29 yr	34.98	100	100	100	100	50.68
CAF	30-34 yr	34.73	100	100	100	100	46.78
CAF	> 34 yr	34.82	100	100	100	100	47.98
CHL	All ages	22.97	100	99.73	87.13	47.39	1.64
CHL	< 20 yr	22.85	100	99.72	86.74	45.81	1.74
CHL	20-29 yr	22.9	100	99.73	86.87	46.76	1.69
CHL	30-34 yr	23.08	100	99.73	87.53	48.51	1.61
CHL	> 34 yr	23.05	100	99.73	87.49	48.36	1.52

ISO	Age group	$PM_{2.5}$ average ($\mu g/m^3$)	Percentage of pregnancies exposed to $PM_{2.5} > C_0$ $\mu g/m^3$				
			$C_0 = 5$	$C_0 = 10$	$C_0 = 15$	$C_0 = 25$	$C_0 = 35$
CHN	All ages	51.11	99.81	99.74	99.61	96.39	77.91
CHN	< 20 yr	50.09	99.8	99.73	99.6	96.14	76.65
CHN	20-29 yr	51.56	99.8	99.72	99.6	96.49	78.13
CHN	30-34 yr	50.27	99.8	99.73	99.59	96.11	77.05
CHN	> 34 yr	50.37	99.86	99.81	99.69	96.39	78.5
CIV	All ages	26.73	100	100	100	75.22	0.71
CIV	< 20 yr	26.71	100	100	100	75.38	0.75
CIV	20-29 yr	26.73	100	100	100	74.99	0.68
CIV	30-34 yr	26.71	100	100	100	74.73	0.73
CIV	> 34 yr	26.79	100	100	100	76.34	0.77
CMR	All ages	58.6	100	100	100	100	94.51
CMR	< 20 yr	59.26	100	100	100	100	94.35
CMR	20-29 yr	58.22	100	100	100	100	94.57
CMR	30-34 yr	58.88	100	100	100	100	94.67
CMR	> 34 yr	58.89	100	100	100	100	94.25
COD	All ages	39.37	100	100	100	98.75	75.1
COD	< 20 yr	39.46	100	100	100	98.87	75.9
COD	20-29 yr	39.35	100	100	100	98.78	75.27
COD	30-34 yr	39.23	100	100	100	98.67	73.82
COD	> 34 yr	39.51	100	100	100	98.67	75.43
COG	All ages	34.92	100	100	100	75.32	60.56
COG	< 20 yr	34.97	100	100	100	76.12	60.42
COG	20-29 yr	34.99	100	100	100	75.66	60.85
COG	30-34 yr	34.79	100	100	100	74.45	59.96
COG	> 34 yr	34.86	100	100	100	74.73	60.59
COL	All ages	18.78	100	99.8	92.68	2.78	0
COL	< 20 yr	18.81	100	99.81	92.41	2.9	0
COL	20-29 yr	18.77	100	99.81	92.54	2.76	0
COL	30-34 yr	18.79	100	99.79	92.93	2.75	0
COL	> 34 yr	18.83	100	99.75	93.26	2.74	0
COM	All ages	11.07	100	99.78	0	0	0
COM	< 20 yr	11.07	100	99.78	0	0	0
COM	20-29 yr	11.07	100	99.78	0	0	0
COM	30-34 yr	11.07	100	99.78	0	0	0
COM	> 34 yr	11.07	100	99.78	0	0	0
CPV	All ages	23.38	100	100	100	27.83	0.2
CPV	< 20 yr	23.21	100	100	100	25.92	0.21
CPV	20-29 yr	23.45	100	100	100	28.21	0.2
CPV	30-34 yr	23.47	100	100	100	28.99	0.2
CPV	> 34 yr	23.15	100	100	100	26.21	0.2
CRI	All ages	19.73	100	100	96.88	0	0
CRI	< 20 yr	19.63	100	100	96.72	0	0

ISO	Age group	$PM_{2.5}$ average ($\mu g/m^3$)	Percentage of pregnancies exposed to $PM_{2.5} > C_0$ $\mu g/m^3$				
			$C_0 = 5$	$C_0 = 10$	$C_0 = 15$	$C_0 = 25$	$C_0 = 35$
CRI	20-29 yr	19.75	100	100	96.94	0	0
CRI	30-34 yr	19.77	100	100	96.93	0	0
CRI	> 34 yr	19.74	100	100	96.75	0	0
CUB	All ages	10.54	100	52.09	0.21	0	0
CUB	< 20 yr	10.51	100	51.38	0.22	0	0
CUB	20-29 yr	10.54	100	52.06	0.21	0	0
CUB	30-34 yr	10.56	100	52.48	0.22	0	0
CUB	> 34 yr	10.55	100	52.69	0.2	0	0
CYP	All ages	18.32	100	100	99.83	0.15	0
CYP	< 20 yr	18.32	100	100	99.83	0.15	0
CYP	20-29 yr	18.32	100	100	99.83	0.15	0
CYP	30-34 yr	18.32	100	100	99.83	0.15	0
CYP	> 34 yr	18.32	100	100	99.83	0.15	0
DJI	All ages	42.68	100	100	100	100	94.93
DJI	< 20 yr	42.68	100	100	100	100	94.93
DJI	20-29 yr	42.68	100	100	100	100	94.93
DJI	30-34 yr	42.68	100	100	100	100	94.93
DJI	> 34 yr	42.68	100	100	100	100	94.93
DMA	All ages	13.94	100	100	2.74	0	0
DMA	< 20 yr	13.96	100	100	2.87	0	0
DMA	20-29 yr	13.93	100	100	2.67	0	0
DMA	30-34 yr	13.94	100	100	2.77	0	0
DMA	> 34 yr	13.95	100	100	2.73	0	0
DOM	All ages	13.27	100	90.43	19.6	0	0
DOM	< 20 yr	13.32	100	90.42	21.04	0	0
DOM	20-29 yr	13.27	100	90.43	19.25	0	0
DOM	30-34 yr	13.23	100	90.41	18.86	0	0
DOM	> 34 yr	13.24	100	90.58	18.88	0	0
DZA	All ages	16.59	100	99.89	47.96	6.22	2.72
DZA	< 20 yr	17.01	100	99.88	51.67	7.33	3.21
DZA	20-29 yr	16.75	100	99.89	49.13	6.7	2.96
DZA	30-34 yr	16.46	100	99.89	47.08	5.84	2.51
DZA	> 34 yr	16.45	100	99.89	46.88	5.81	2.54
ECU	All ages	18.21	100	99.85	78.22	1.91	0
ECU	< 20 yr	18.09	100	99.89	76.86	1.97	0
ECU	20-29 yr	18.19	100	99.85	78.03	1.94	0
ECU	30-34 yr	18.32	100	99.84	79.59	1.84	0
ECU	> 34 yr	18.29	100	99.83	79.3	1.82	0
EGY	All ages	44.42	100	100	100	99.6	86.54
EGY	< 20 yr	44.81	100	100	100	99.62	87.4
EGY	20-29 yr	44.44	100	100	100	99.61	86.66
EGY	30-34 yr	44.24	100	100	100	99.58	86.05

ISO	Age group	$PM_{2.5}$ average ($\mu g/m^3$)	Percentage of pregnancies exposed to $PM_{2.5} > C_0$ $\mu g/m^3$				
			$C_0 = 5$	$C_0 = 10$	$C_0 = 15$	$C_0 = 25$	$C_0 = 35$
EGY	> 34 yr	44.29	100	100	100	99.58	85.99
ERI	All ages	34.48	100	100	100	99.8	27.73
ERI	< 20 yr	34.48	100	100	100	99.8	27.73
ERI	20-29 yr	34.48	100	100	100	99.8	27.73
ERI	30-34 yr	34.48	100	100	100	99.8	27.73
ERI	> 34 yr	34.48	100	100	100	99.8	27.73
ETH	All ages	24.88	100	100	98.94	38.75	4.64
ETH	< 20 yr	24.85	100	100	98.89	38.61	4.63
ETH	20-29 yr	24.82	100	100	99.02	38.23	4.28
ETH	30-34 yr	24.93	100	100	98.86	39.15	4.96
ETH	> 34 yr	25.05	100	100	98.83	39.97	5.36
GAB	All ages	29.03	100	100	100	82.94	13.7
GAB	< 20 yr	29.08	100	100	100	82.31	14.22
GAB	20-29 yr	29.1	100	100	100	83.78	13.78
GAB	30-34 yr	28.93	100	100	100	82.66	13.32
GAB	> 34 yr	28.85	100	100	100	81.21	13.27
GEO	All ages	22.7	100	100	100	38.56	0
GEO	< 20 yr	22.57	100	100	100	36.63	0
GEO	20-29 yr	22.78	100	100	100	39.46	0
GEO	30-34 yr	22.64	100	100	100	37.87	0
GEO	> 34 yr	22.49	100	100	100	36.13	0
GHA	All ages	32.38	100	100	100	95.9	26.89
GHA	< 20 yr	32.26	100	100	100	95.96	25.94
GHA	20-29 yr	32.31	100	100	100	95.9	26.43
GHA	30-34 yr	32.54	100	100	100	95.95	28.04
GHA	> 34 yr	32.45	100	100	100	95.85	27.33
GIN	All ages	31.13	100	100	100	99.89	5.18
GIN	< 20 yr	31.11	100	100	100	99.88	5.17
GIN	20-29 yr	31.15	100	100	100	99.89	5.19
GIN	30-34 yr	31.15	100	100	100	99.89	5.23
GIN	> 34 yr	31.06	100	100	100	99.89	5.07
GMB	All ages	43	100	100	100	100	100
GMB	< 20 yr	43.07	100	100	100	100	100
GMB	20-29 yr	42.92	100	100	100	100	100
GMB	30-34 yr	43.08	100	100	100	100	100
GMB	> 34 yr	43.11	100	100	100	100	100
GNB	All ages	34.85	100	100	100	99.94	42.98
GNB	< 20 yr	34.88	100	100	100	99.94	43.18
GNB	20-29 yr	34.83	100	100	100	99.94	42.5
GNB	30-34 yr	34.85	100	100	100	99.94	43.11
GNB	> 34 yr	34.87	100	100	100	99.94	44.03
GNQ	All ages	37.42	100	100	97.97	97.83	67.8

ISO	Age group	$PM_{2.5}$ average ($\mu g/m^3$)	Percentage of pregnancies exposed to $PM_{2.5} > C_0$ $\mu g/m^3$				
			$C_0 = 5$	$C_0 = 10$	$C_0 = 15$	$C_0 = 25$	$C_0 = 35$
GNQ	< 20 yr	37.23	100	100	97.89	97.74	67.38
GNQ	20-29 yr	37.73	100	100	98.81	98.67	68.52
GNQ	30-34 yr	37.03	100	100	97.06	96.9	66.67
GNQ	> 34 yr	37.22	100	100	96.83	96.69	67.71
GRD	All ages	15.74	100	100	97.79	0	0
GRD	< 20 yr	15.72	100	100	97.88	0	0
GRD	20-29 yr	15.74	100	100	97.78	0	0
GRD	30-34 yr	15.74	100	100	97.75	0	0
GRD	> 34 yr	15.74	100	100	97.8	0	0
GTM	All ages	29.64	100	100	99.82	67.94	19.8
GTM	< 20 yr	29.63	100	100	99.82	67.9	19.74
GTM	20-29 yr	29.65	100	100	99.82	67.95	19.81
GTM	30-34 yr	29.65	100	100	99.82	67.95	19.81
GTM	> 34 yr	29.65	100	100	99.82	67.95	19.81
GUY	All ages	20.43	100	100	99.99	0	0
GUY	< 20 yr	20.47	100	100	99.99	0	0
GUY	20-29 yr	20.42	100	100	99.99	0	0
GUY	30-34 yr	20.42	100	100	99.99	0	0
GUY	> 34 yr	20.43	100	100	99.99	0	0
HND	All ages	30.19	100	100	99.84	87.64	16.36
HND	< 20 yr	30.09	100	100	99.85	87.03	16.14
HND	20-29 yr	30.18	100	100	99.83	87.59	16.33
HND	30-34 yr	30.28	100	100	99.83	88.2	16.67
HND	> 34 yr	30.25	100	100	99.84	88.13	16.43
HTI	All ages	14.88	100	99.81	47.68	0	0
HTI	< 20 yr	14.9	100	99.8	47.57	0	0
HTI	20-29 yr	14.88	100	99.81	48.31	0	0
HTI	30-34 yr	14.86	100	99.82	47.44	0	0
HTI	> 34 yr	14.86	100	99.82	46.44	0	0
IDN	All ages	26.19	100	99.28	85.96	45.39	18.43
IDN	< 20 yr	26.04	100	99.27	85.35	44.56	18.17
IDN	20-29 yr	26.36	100	99.3	86.09	46.17	19.02
IDN	30-34 yr	26.1	100	99.28	86	45	18.08
IDN	> 34 yr	25.8	100	99.23	85.72	43.66	17.06
IND	All ages	60.15	100	99.96	99.64	94	79.2
IND	< 20 yr	62.39	100	99.97	99.7	94.96	81.75
IND	20-29 yr	60.03	99.99	99.96	99.64	94.06	79.12
IND	30-34 yr	60.35	99.99	99.96	99.62	93.84	79.38
IND	> 34 yr	59.45	100	99.96	99.58	93.14	77.91
IRN	All ages	40.53	100	100	100	98.63	60.39
IRN	< 20 yr	41.29	100	100	100	98.61	62.97
IRN	20-29 yr	40.7	100	100	100	98.67	61.09

ISO	Age group	$PM_{2.5}$ average ($\mu g/m^3$)	Percentage of pregnancies exposed to $PM_{2.5} > C_0$ $\mu g/m^3$				
			$C_0 = 5$	$C_0 = 10$	$C_0 = 15$	$C_0 = 25$	$C_0 = 35$
IRN	30-34 yr	40.3	100	100	100	98.63	59.26
IRN	> 34 yr	39.99	100	100	100	98.5	58.41
IRQ	All ages	51.92	100	100	100	100	96.94
IRQ	< 20 yr	52.28	100	100	100	100	97.21
IRQ	20-29 yr	51.7	100	100	100	100	96.8
IRQ	30-34 yr	52.25	100	100	100	100	97.04
IRQ	> 34 yr	51.95	100	100	100	100	97.09
ISR	All ages	22.66	100	100	100	9.92	0.02
ISR	< 20 yr	22.6	100	100	100	10.57	0.02
ISR	20-29 yr	22.67	100	100	100	10.11	0.02
ISR	30-34 yr	22.67	100	100	100	9.64	0.02
ISR	> 34 yr	22.65	100	100	100	9.82	0.02
JAM	All ages	21.18	100	100	91.78	29.74	0
JAM	< 20 yr	20.99	100	100	91.29	28.04	0
JAM	20-29 yr	21.22	100	100	92.03	30.11	0
JAM	30-34 yr	21.12	100	100	91.48	29.01	0
JAM	> 34 yr	21.32	100	100	91.75	30.99	0
JOR	All ages	34.17	100	100	100	91.1	39.37
JOR	< 20 yr	33.9	100	100	100	90.57	37.85
JOR	20-29 yr	34.15	100	100	100	91.05	39.25
JOR	30-34 yr	34.2	100	100	100	91.16	39.42
JOR	> 34 yr	34.31	100	100	100	91.36	40.18
JPN	All ages	13.12	100	90.75	21.79	0	0
JPN	< 20 yr	13.03	100	90.11	20.56	0	0
JPN	20-29 yr	13.15	100	90.85	22.34	0	0
JPN	30-34 yr	13.12	100	90.71	21.76	0	0
JPN	> 34 yr	13.1	100	90.71	21.2	0	0
KAZ	All ages	22.98	100	99.81	68.95	47.78	9.02
KAZ	< 20 yr	22.49	100	99.8	66.9	45.21	8.39
KAZ	20-29 yr	22.93	100	99.81	68.9	47.62	8.82
KAZ	30-34 yr	23.12	100	99.81	69.43	48.46	9.34
KAZ	> 34 yr	23.16	100	99.81	69.14	48.42	9.79
KEN	All ages	20.92	100	99.92	90.1	22.01	0
KEN	< 20 yr	21.14	100	99.92	90.92	24.73	0
KEN	20-29 yr	20.95	100	99.92	89.8	22.21	0
KEN	30-34 yr	20.74	100	99.91	89.94	20.51	0
KEN	> 34 yr	20.8	100	99.91	90.96	20.67	0
KGZ	All ages	23.9	100	100	96.31	35.58	0.22
KGZ	< 20 yr	23.95	100	100	96.32	36.57	0.21
KGZ	20-29 yr	23.94	100	100	96.35	35.91	0.2
KGZ	30-34 yr	23.8	100	100	96.14	34.46	0.26
KGZ	> 34 yr	23.83	100	100	96.28	34.68	0.25

ISO	Age group	$PM_{2.5}$ average ($\mu g/m^3$)	Percentage of pregnancies exposed to $PM_{2.5} > C_0$ $\mu g/m^3$				
			$C_0 = 5$	$C_0 = 10$	$C_0 = 15$	$C_0 = 25$	$C_0 = 35$
KHM	All ages	17.58	100	100	95.45	0	0
KHM	< 20 yr	17.6	100	100	95.45	0	0
KHM	20-29 yr	17.6	100	100	95.64	0	0
KHM	30-34 yr	17.53	100	100	95.04	0	0
KHM	> 34 yr	17.55	100	100	95.08	0	0
KNA	All ages	12.82	100	100	0.76	0	0
KNA	< 20 yr	12.81	100	100	0.82	0	0
KNA	20-29 yr	12.84	100	100	0.75	0	0
KNA	30-34 yr	12.81	100	100	0.74	0	0
KNA	> 34 yr	12.81	100	100	0.73	0	0
KOR	All ages	24.73	100	100	100	40.41	0
KOR	< 20 yr	24.58	100	100	100	38.21	0
KOR	20-29 yr	24.74	100	100	100	40.47	0
KOR	30-34 yr	24.73	100	100	100	40.53	0
KOR	> 34 yr	24.68	100	100	100	39.9	0
KWT	All ages	64.84	100	100	100	100	100
KWT	< 20 yr	64.12	100	100	100	100	100
KWT	20-29 yr	64.66	100	100	100	100	100
KWT	30-34 yr	64.92	100	100	100	100	100
KWT	> 34 yr	65.07	100	100	100	100	100
LAO	All ages	26.71	100	100	100	61.74	3.2
LAO	< 20 yr	26.79	100	100	100	62.47	3.36
LAO	20-29 yr	26.73	100	100	100	61.91	3.21
LAO	30-34 yr	26.63	100	100	100	61.07	3.1
LAO	> 34 yr	26.61	100	100	100	60.68	3.1
LBN	All ages	30.99	100	100	100	88.45	23.62
LBN	< 20 yr	30.99	100	100	100	88.45	23.62
LBN	20-29 yr	30.99	100	100	100	88.45	23.63
LBN	30-34 yr	30.99	100	100	100	88.45	23.62
LBN	> 34 yr	30.99	100	100	100	88.45	23.62
LBR	All ages	25.8	100	100	100	63.34	0
LBR	< 20 yr	25.76	100	100	100	62.33	0
LBR	20-29 yr	25.79	100	100	100	63.28	0
LBR	30-34 yr	25.81	100	100	100	63.6	0
LBR	> 34 yr	25.84	100	100	100	64.3	0
LBY	All ages	21.54	100	100	98.49	16.64	5.86
LBY	< 20 yr	21.54	100	100	98.49	16.64	5.86
LBY	20-29 yr	21.54	100	100	98.49	16.64	5.86
LBY	30-34 yr	21.54	100	100	98.49	16.64	5.86
LBY	> 34 yr	21.54	100	100	98.49	16.64	5.86
LCA	All ages	15.03	100	100	50.57	0	0
LCA	< 20 yr	15.03	100	100	50.57	0	0

ISO	Age group	$PM_{2.5}$ average ($\mu g/m^3$)	Percentage of pregnancies exposed to $PM_{2.5} > C_0$ $\mu g/m^3$				
			$C_0 = 5$	$C_0 = 10$	$C_0 = 15$	$C_0 = 25$	$C_0 = 35$
LCA	20-29 yr	15.03	100	100	50.57	0	0
LCA	30-34 yr	15.03	100	100	50.57	0	0
LCA	> 34 yr	15.03	100	100	50.57	0	0
LKA	All ages	25.3	100	100	99.99	44.49	0
LKA	< 20 yr	25.23	100	100	99.98	43.75	0
LKA	20-29 yr	25.26	100	100	99.99	43.77	0
LKA	30-34 yr	25.35	100	100	99.99	45.3	0
LKA	> 34 yr	25.34	100	100	99.99	45.28	0
LSO	All ages	26.77	100	100	100	68.24	0.01
LSO	< 20 yr	26.62	100	100	100	66.14	0.01
LSO	20-29 yr	26.8	100	100	100	68.68	0.01
LSO	30-34 yr	26.79	100	100	100	68.52	0.01
LSO	> 34 yr	26.77	100	100	100	68.1	0.01
MAR	All ages	20.78	100	99.99	92.71	11.33	0.01
MAR	< 20 yr	20.62	100	99.99	92.06	10.78	0.01
MAR	20-29 yr	20.73	100	99.99	92.68	11.18	0.01
MAR	30-34 yr	20.84	100	99.99	92.95	11.62	0.01
MAR	> 34 yr	20.85	100	99.99	92.74	11.51	0.01
MDG	All ages	15.06	99.99	84.32	49.15	0.01	0
MDG	< 20 yr	14.95	99.99	83.8	48.48	0.01	0
MDG	20-29 yr	15.02	99.99	84.21	48.77	0.01	0
MDG	30-34 yr	15.15	100	84.34	49.65	0.01	0
MDG	> 34 yr	15.26	100	85.48	51.07	0.01	0
MDV	All ages	20.05	100	96.34	69.23	17.41	0
MDV	< 20 yr	20.05	100	96.34	69.23	17.41	0
MDV	20-29 yr	20.05	100	96.34	69.23	17.41	0
MDV	30-34 yr	20.05	100	96.34	69.23	17.41	0
MDV	> 34 yr	20.05	100	96.34	69.23	17.41	0
MEX	All ages	19.22	99.79	90.56	73.78	17.43	0
MEX	< 20 yr	19.03	99.78	90.25	72.97	16.42	0
MEX	20-29 yr	19.22	99.8	90.66	73.87	17.38	0
MEX	30-34 yr	19.29	99.78	90.64	74.07	17.89	0
MEX	> 34 yr	19.3	99.78	90.34	73.96	18.25	0
MLI	All ages	40.22	100	100	100	100	70.38
MLI	< 20 yr	39.96	100	100	100	100	68.3
MLI	20-29 yr	40.18	100	100	100	100	69.99
MLI	30-34 yr	40.37	100	100	100	100	71.76
MLI	> 34 yr	40.5	100	100	100	100	72.65
MMR	All ages	35	100	100	99.84	94.85	43.25
MMR	< 20 yr	34.82	100	100	99.83	94.37	41.71
MMR	20-29 yr	34.99	100	100	99.84	94.82	43.05
MMR	30-34 yr	35.05	100	100	99.85	94.93	43.8

ISO	Age group	$PM_{2.5}$ average ($\mu g/m^3$)	Percentage of pregnancies exposed to $PM_{2.5} > C_0$ $\mu g/m^3$				
			$C_0 = 5$	$C_0 = 10$	$C_0 = 15$	$C_0 = 25$	$C_0 = 35$
MMR	> 34 yr	35.06	100	100	99.85	95.07	44.03
MNG	All ages	40.69	100	99.97	89.11	54.34	48.04
MNG	< 20 yr	40.97	100	99.97	89.18	55.01	48.67
MNG	20-29 yr	41.02	100	99.97	88.91	54.99	48.83
MNG	30-34 yr	39.79	100	99.96	89.2	52.46	45.98
MNG	> 34 yr	40.38	100	99.97	89.85	53.81	47.16
MOZ	All ages	18.98	100	99.67	79.07	3.76	0
MOZ	< 20 yr	18.98	100	99.67	78.96	3.88	0
MOZ	20-29 yr	19.03	100	99.68	79.09	3.83	0
MOZ	30-34 yr	18.92	100	99.68	79.42	3.51	0
MOZ	> 34 yr	18.88	100	99.64	78.81	3.58	0
MRT	All ages	52.46	100	100	100	100	100
MRT	< 20 yr	52.61	100	100	100	100	100
MRT	20-29 yr	52.4	100	100	100	100	100
MRT	30-34 yr	52.41	100	100	100	100	100
MRT	> 34 yr	52.55	100	100	100	100	100
MUS	All ages	14.74	100	96.63	55.61	0	0
MUS	< 20 yr	14.73	100	96.4	55.66	0	0
MUS	20-29 yr	14.74	100	96.58	55.82	0	0
MUS	30-34 yr	14.74	100	96.67	55.13	0	0
MUS	> 34 yr	14.76	100	96.9	55.56	0	0
MWI	All ages	21.93	100	100	100	6.25	0
MWI	< 20 yr	21.91	100	100	100	6.6	0
MWI	20-29 yr	21.93	100	100	100	6.01	0
MWI	30-34 yr	21.94	100	100	100	6.21	0
MWI	> 34 yr	21.95	100	100	100	6.79	0
MYS	All ages	24.16	100	100	95.11	44.35	0
MYS	< 20 yr	23.66	100	100	95.19	38.43	0
MYS	20-29 yr	24.25	100	100	94.94	45.78	0
MYS	30-34 yr	24.09	100	100	95.16	43.36	0
MYS	> 34 yr	24.15	100	100	95.45	43.38	0
NAM	All ages	17.88	100	98.08	68.64	7.46	0
NAM	< 20 yr	18.45	100	98.38	75.12	7.6	0
NAM	20-29 yr	17.89	100	98.11	68.62	7.59	0
NAM	30-34 yr	17.57	100	97.91	65.62	7.1	0
NAM	> 34 yr	17.68	100	97.9	66.63	7.27	0
NER	All ages	77.32	100	100	100	100	100
NER	< 20 yr	78.28	100	100	100	100	100
NER	20-29 yr	76.98	100	100	100	100	100
NER	30-34 yr	77.07	100	100	100	100	100
NER	> 34 yr	77.4	100	100	100	100	100
NGA	All ages	69.66	100	100	100	98.9	90.59

ISO	Age group	$PM_{2.5}$ average ($\mu g/m^3$)	Percentage of pregnancies exposed to $PM_{2.5} > C_0$ $\mu g/m^3$				
			$C_0 = 5$	$C_0 = 10$	$C_0 = 15$	$C_0 = 25$	$C_0 = 35$
NGA	< 20 yr	70.01	100	100	100	98.85	90.4
NGA	20-29 yr	70.3	100	100	100	98.9	90.81
NGA	30-34 yr	70.13	100	100	100	99	90.88
NGA	> 34 yr	66.96	100	100	100	98.84	89.78
NIC	All ages	20.95	100	100	98.41	21.1	0
NIC	< 20 yr	20.93	100	100	98.37	20.74	0
NIC	20-29 yr	20.98	100	100	98.4	21.45	0
NIC	30-34 yr	20.95	100	100	98.45	20.96	0
NIC	> 34 yr	20.87	100	100	98.49	20.25	0
NPL	All ages	50.49	100	100	99.97	97.96	77.62
NPL	< 20 yr	50.54	100	100	99.97	97.98	77.79
NPL	20-29 yr	50.39	100	100	99.97	97.95	77.48
NPL	30-34 yr	50.83	100	100	99.98	98.01	77.99
NPL	> 34 yr	50.76	100	100	99.97	97.94	77.86
OMN	All ages	47.5	100	100	100	100	97.1
OMN	< 20 yr	47.46	100	100	100	100	96.89
OMN	20-29 yr	47.48	100	100	100	100	97.02
OMN	30-34 yr	47.51	100	100	100	100	97.15
OMN	> 34 yr	47.52	100	100	100	100	97.26
PAK	All ages	63.16	99.99	99.81	99.37	98.13	93.92
PAK	< 20 yr	63.07	99.99	99.81	99.36	98.17	94.06
PAK	20-29 yr	63.28	99.99	99.81	99.38	98.12	93.87
PAK	30-34 yr	62.97	99.99	99.81	99.37	98.13	93.97
PAK	> 34 yr	62.87	99.99	99.81	99.36	98.11	94.01
PAN	All ages	17.23	100	100	89.17	0	0
PAN	< 20 yr	17.18	100	100	88.61	0	0
PAN	20-29 yr	17.26	100	100	89.57	0	0
PAN	30-34 yr	17.22	100	100	88.65	0	0
PAN	> 34 yr	17.25	100	100	89.02	0	0
PER	All ages	26.59	99.95	98.42	92.64	60.43	17.69
PER	< 20 yr	26.42	99.96	98.49	92.86	59.58	16.61
PER	20-29 yr	26.63	99.95	98.41	92.64	60.66	17.89
PER	30-34 yr	26.64	99.95	98.4	92.56	60.68	17.99
PER	> 34 yr	26.55	99.95	98.41	92.54	60.09	17.56
PHL	All ages	22.52	100	99.84	92.46	29.09	0
PHL	< 20 yr	22.35	100	99.84	92.02	27.37	0
PHL	20-29 yr	22.57	100	99.84	92.63	29.58	0
PHL	30-34 yr	22.51	100	99.85	92.4	29.19	0
PHL	> 34 yr	22.44	100	99.84	92.18	28.49	0
PNG	All ages	15.53	100	99.69	60.36	0.06	0
PNG	< 20 yr	15.53	100	99.69	60.47	0.05	0
PNG	20-29 yr	15.53	100	99.69	60.39	0.06	0

ISO	Age group	$PM_{2.5}$ average ($\mu g/m^3$)	Percentage of pregnancies exposed to $PM_{2.5} > C_0$ $\mu g/m^3$				
			$C_0 = 5$	$C_0 = 10$	$C_0 = 15$	$C_0 = 25$	$C_0 = 35$
PNG	30-34 yr	15.52	100	99.69	60.3	0.06	0
PNG	> 34 yr	15.52	100	99.69	60.31	0.06	0
PRK	All ages	29.26	100	100	100	63	25.06
PRK	< 20 yr	29.09	100	100	100	62	24.21
PRK	20-29 yr	29.25	100	100	100	62.99	24.97
PRK	30-34 yr	29.26	100	100	100	63	25.15
PRK	> 34 yr	29.26	100	100	100	63.05	25.01
PRY	All ages	15.08	100	100	46.72	0	0
PRY	< 20 yr	15.07	100	100	46.68	0	0
PRY	20-29 yr	15.09	100	100	47.07	0	0
PRY	30-34 yr	15.07	100	100	46.17	0	0
PRY	> 34 yr	15.07	100	100	46.22	0	0
PSE	All ages	23.91	100	100	100	23.93	0
PSE	< 20 yr	23.84	100	100	100	23.19	0
PSE	20-29 yr	23.9	100	100	100	23.98	0
PSE	30-34 yr	23.95	100	100	100	24.38	0
PSE	> 34 yr	23.97	100	100	100	24.43	0
QAT	All ages	85.98	100	100	100	100	100
QAT	< 20 yr	85.98	100	100	100	100	100
QAT	20-29 yr	85.98	100	100	100	100	100
QAT	30-34 yr	85.98	100	100	100	100	100
QAT	> 34 yr	85.98	100	100	100	100	100
RWA	All ages	38.59	100	100	100	100	96.25
RWA	< 20 yr	38.66	100	100	100	100	96.3
RWA	20-29 yr	38.62	100	100	100	100	96.28
RWA	30-34 yr	38.55	100	100	100	100	96.19
RWA	> 34 yr	38.57	100	100	100	100	96.22
SAU	All ages	73.75	100	100	100	99.91	98.56
SAU	< 20 yr	73.1	100	100	100	99.9	98.53
SAU	20-29 yr	73.61	100	100	100	99.9	98.47
SAU	30-34 yr	73.88	100	100	100	99.91	98.56
SAU	> 34 yr	73.86	100	100	100	99.91	98.68
SDN	All ages	38.25	100	100	100	98.35	70.56
SDN	< 20 yr	38.06	100	100	100	98.46	69.52
SDN	20-29 yr	38.15	100	100	100	98.19	70.08
SDN	30-34 yr	38.35	100	100	100	98.49	70.86
SDN	> 34 yr	38.57	100	100	100	98.53	72.53
SEN	All ages	47.12	100	100	100	100	99.92
SEN	< 20 yr	47.22	100	100	100	100	99.92
SEN	20-29 yr	47.15	100	100	100	100	99.92
SEN	30-34 yr	47.1	100	100	100	100	99.91
SEN	> 34 yr	47.02	100	100	100	100	99.91

ISO	Age group	$PM_{2.5}$ average ($\mu g/m^3$)	Percentage of pregnancies exposed to $PM_{2.5} > C_0$ $\mu g/m^3$				
			$C_0 = 5$	$C_0 = 10$	$C_0 = 15$	$C_0 = 25$	$C_0 = 35$
SGP	All ages	27.19	100	100	100	100	0
SGP	< 20 yr	27.19	100	100	100	100	0
SGP	20-29 yr	27.19	100	100	100	100	0
SGP	30-34 yr	27.19	100	100	100	100	0
SGP	> 34 yr	27.19	100	100	100	100	0
SLB	All ages	11.36	100	99.4	0	0	0
SLB	< 20 yr	11.36	100	99.42	0	0	0
SLB	20-29 yr	11.36	100	99.4	0	0	0
SLB	30-34 yr	11.36	100	99.39	0	0	0
SLB	> 34 yr	11.36	100	99.39	0	0	0
SLE	All ages	29.06	100	100	100	99.56	0
SLE	< 20 yr	29.07	100	100	100	99.57	0
SLE	20-29 yr	29.04	100	100	100	99.56	0
SLE	30-34 yr	29.07	100	100	100	99.56	0
SLE	> 34 yr	29.09	100	100	100	99.56	0
SLV	All ages	29.38	100	100	100	88.2	1.02
SLV	< 20 yr	29.49	100	100	100	88.87	1.13
SLV	20-29 yr	29.32	100	100	100	87.84	1.03
SLV	30-34 yr	29.33	100	100	100	87.79	0.91
SLV	> 34 yr	29.59	100	100	100	89.4	0.9
SOM	All ages	19.13	100	99.75	62.59	22.98	3.35
SOM	< 20 yr	19.43	100	99.76	64.07	24.71	3.46
SOM	20-29 yr	19.14	100	99.75	62.65	23.08	3.35
SOM	30-34 yr	18.77	100	99.74	60.89	21	3.17
SOM	> 34 yr	19.18	100	99.75	62.88	23.23	3.43
SSD	All ages	24.79	100	100	99.99	43.36	0.24
SSD	< 20 yr	24.71	100	100	99.99	42.53	0.25
SSD	20-29 yr	24.79	100	100	99.99	43.16	0.22
SSD	30-34 yr	24.82	100	100	99.99	43.65	0.26
SSD	> 34 yr	24.86	100	100	99.99	44.54	0.25
STP	All ages	19.56	100	100	100	10.16	0
STP	< 20 yr	19.67	100	100	100	10.49	0
STP	20-29 yr	19.53	100	100	100	10.13	0
STP	30-34 yr	19.54	100	100	100	10.06	0
STP	> 34 yr	19.59	100	100	100	10.12	0
SUR	All ages	19.93	100	100	99.93	0	0
SUR	< 20 yr	19.93	100	100	99.96	0	0
SUR	20-29 yr	19.95	100	100	99.94	0	0
SUR	30-34 yr	19.86	100	100	99.91	0	0
SUR	> 34 yr	19.91	100	100	99.92	0	0
SWZ	All ages	18.1	100	100	90.67	0.02	0
SWZ	< 20 yr	17.97	100	100	90.44	0.02	0

ISO	Age group	$PM_{2.5}$ average ($\mu g/m^3$)	Percentage of pregnancies exposed to $PM_{2.5} > C_0$ $\mu g/m^3$				
			$C_0 = 5$	$C_0 = 10$	$C_0 = 15$	$C_0 = 25$	$C_0 = 35$
SWZ	20-29 yr	18.13	100	100	90.74	0.02	0
SWZ	30-34 yr	18.13	100	100	90.71	0.02	0
SWZ	> 34 yr	18.08	100	100	90.56	0.02	0
SYC	All ages	9.13	100	2.71	0	0	0
SYC	< 20 yr	9.13	100	2.71	0	0	0
SYC	20-29 yr	9.13	100	2.71	0	0	0
SYC	30-34 yr	9.13	100	2.71	0	0	0
SYC	> 34 yr	9.13	100	2.71	0	0	0
SYR	All ages	35.21	100	100	100	98.79	47.71
SYR	< 20 yr	35.21	100	100	100	98.79	47.71
SYR	20-29 yr	35.21	100	100	100	98.79	47.72
SYR	30-34 yr	35.21	100	100	100	98.79	47.71
SYR	> 34 yr	35.21	100	100	100	98.79	47.71
TCD	All ages	43.16	100	100	100	99.95	66.2
TCD	< 20 yr	44.19	100	100	100	99.96	68.35
TCD	20-29 yr	42.91	100	100	100	99.95	65.95
TCD	30-34 yr	42.72	100	100	100	99.95	64.89
TCD	> 34 yr	43.17	100	100	100	99.94	65.75
TGO	All ages	39	100	100	100	100	57
TGO	< 20 yr	39.33	100	100	100	100	59.01
TGO	20-29 yr	38.87	100	100	100	100	56.07
TGO	30-34 yr	38.96	100	100	100	100	56.82
TGO	> 34 yr	39.24	100	100	100	100	58.96
THA	All ages	23.22	100	100	99.96	30.88	0.29
THA	< 20 yr	23.47	100	100	99.97	32.89	0.31
THA	20-29 yr	23.06	100	100	99.96	29.42	0.26
THA	30-34 yr	23.28	100	100	99.96	31.45	0.3
THA	> 34 yr	23.54	100	100	99.97	33.99	0.36
TJK	All ages	32.42	100	99.41	97.46	86.13	34.33
TJK	< 20 yr	32.54	100	99.5	97.73	86.38	34.5
TJK	20-29 yr	32.43	100	99.43	97.5	86.11	34.38
TJK	30-34 yr	32.32	100	99.33	97.2	86.02	34.1
TJK	> 34 yr	32.38	100	99.35	97.28	86.33	34.19
TKM	All ages	32.17	100	100	100	88.55	27.55
TKM	< 20 yr	32.18	100	100	100	87.84	28.38
TKM	20-29 yr	32.23	100	100	100	88.83	27.86
TKM	30-34 yr	32.02	100	100	100	87.72	26.84
TKM	> 34 yr	32.12	100	100	100	88.62	26.53
TLS	All ages	12.65	100	99.64	0.81	0	0
TLS	< 20 yr	12.62	100	99.69	0.74	0	0
TLS	20-29 yr	12.7	100	99.62	0.89	0	0
TLS	30-34 yr	12.59	100	99.62	0.7	0	0

ISO	Age group	$PM_{2.5}$ average ($\mu g/m^3$)	Percentage of pregnancies exposed to $PM_{2.5}$ > C_0 $\mu g/m^3$				
			$C_0 = 5$	$C_0 = 10$	$C_0 = 15$	$C_0 = 25$	$C_0 = 35$
TLS	> 34 yr	12.59	100	99.69	0.71	0	0
TTO	All ages	17.5	100	100	94.13	0	0
TTO	< 20 yr	17.48	100	100	94.13	0	0
TTO	20-29 yr	17.51	100	100	94.27	0	0
TTO	30-34 yr	17.48	100	100	93.91	0	0
TTO	> 34 yr	17.48	100	100	94.04	0	0
TUN	All ages	19.61	100	99.99	97.23	2.42	0.1
TUN	< 20 yr	19.53	100	99.99	97.25	2.35	0.1
TUN	20-29 yr	19.63	100	99.99	97.33	2.46	0.1
TUN	30-34 yr	19.62	100	99.99	97.16	2.39	0.1
TUN	> 34 yr	19.58	100	99.99	97.15	2.37	0.1
TUR	All ages	31.08	100	100	100	81.08	28.4
TUR	< 20 yr	31.75	100	100	100	83.1	32.92
TUR	20-29 yr	31.2	100	100	100	81.41	29.25
TUR	30-34 yr	30.83	100	100	100	80.32	26.72
TUR	> 34 yr	30.66	100	100	100	79.95	25.39
TZA	All ages	22.01	100	99.98	95.29	21.15	0.89
TZA	< 20 yr	22.17	100	99.98	95.15	22.19	0.91
TZA	20-29 yr	22.01	100	99.98	95.47	21.08	0.87
TZA	30-34 yr	21.93	100	99.98	95.35	20.76	0.89
TZA	> 34 yr	21.88	100	99.98	94.81	20.66	0.91
UGA	All ages	31.18	100	100	99.97	75.62	41.09
UGA	< 20 yr	31.32	100	100	99.97	75.99	42.17
UGA	20-29 yr	31.24	100	100	99.97	75.73	41.59
UGA	30-34 yr	30.95	100	100	99.97	74.99	39.24
UGA	> 34 yr	31.03	100	100	99.98	75.35	39.58
URY	All ages	11.91	100	87.18	0	0	0
URY	< 20 yr	11.74	100	84.59	0	0	0
URY	20-29 yr	11.97	100	88.02	0	0	0
URY	30-34 yr	11.92	100	87.53	0	0	0
URY	> 34 yr	11.88	100	86.88	0	0	0
UZB	All ages	34.43	100	100	100	92.36	41.93
UZB	< 20 yr	34.53	100	100	100	92.3	42.67
UZB	20-29 yr	34.39	100	100	100	92.31	41.73
UZB	30-34 yr	34.46	100	100	100	92.49	42.13
UZB	> 34 yr	34.65	100	100	100	92.75	43
VCT	All ages	16.26	100	100	92.74	0	0
VCT	< 20 yr	16.26	100	100	92.43	0	0
VCT	20-29 yr	16.26	100	100	92.78	0	0
VCT	30-34 yr	16.26	100	100	92.86	0	0
VCT	> 34 yr	16.26	100	100	92.73	0	0
VEN	All ages	21.61	100	99.94	94.85	11.19	0

ISO	Age group	$PM_{2.5}$ average ($\mu g/m^3$)	Percentage of pregnancies exposed to $PM_{2.5} > C_0$ $\mu g/m^3$				
			$C_0 = 5$	$C_0 = 10$	$C_0 = 15$	$C_0 = 25$	$C_0 = 35$
VEN	< 20 yr	21.56	100	99.94	94.77	11.62	0
VEN	20-29 yr	21.61	100	99.94	94.88	11.11	0
VEN	30-34 yr	21.64	100	99.94	94.88	10.91	0
VEN	> 34 yr	21.65	100	99.94	94.88	10.97	0
VNM	All ages	27.93	100	100	99.86	54.67	26
VNM	< 20 yr	28	100	100	99.85	54.95	26.2
VNM	20-29 yr	28.01	100	100	99.86	55.13	26.37
VNM	30-34 yr	27.79	100	100	99.85	53.88	25.43
VNM	> 34 yr	27.61	100	100	99.84	53	24.54
YEM	All ages	45.8	100	100	100	99.86	97.82
YEM	< 20 yr	45.65	100	100	100	99.87	97.86
YEM	20-29 yr	45.76	100	100	100	99.87	97.88
YEM	30-34 yr	45.93	100	100	100	99.84	97.64
YEM	> 34 yr	45.92	100	100	100	99.86	97.71
ZAF	All ages	25.93	99.91	88	74.32	36.21	24.63
ZAF	< 20 yr	23.7	99.93	89.76	73.44	29.54	17.84
ZAF	20-29 yr	26.17	99.91	88.06	74.64	36.75	25.22
ZAF	30-34 yr	26.63	99.91	87.52	74.66	38.35	26.8
ZAF	> 34 yr	25.92	99.91	86.74	73.15	36.85	25.12
ZMB	All ages	23.48	100	100	100	37.48	0
ZMB	< 20 yr	23.55	100	100	100	37.92	0
ZMB	20-29 yr	23.42	100	100	100	36.96	0
ZMB	30-34 yr	23.51	100	100	100	37.9	0
ZMB	> 34 yr	23.61	100	100	100	38.37	0
ZWE	All ages	18.82	100	100	92.22	0.16	0
ZWE	< 20 yr	18.75	100	100	91.89	0.17	0
ZWE	20-29 yr	18.86	100	100	92.43	0.15	0
ZWE	30-34 yr	18.81	100	100	92.15	0.16	0
ZWE	> 34 yr	18.76	100	100	91.64	0.18	0

Supplementary Table 4 Country-level estimates on fraction or number of stillbirths attributable to $PM_{2.5}$ in 2015 referring to different exposure levels of minimum risk and using different exposure-response methods.

ISO	Total number of stillbirths	Method	Fraction of stillbirths attributable to $PM_{2.5} > C_0$ μg/m³					Number of stillbirths attributable to $PM_{2.5} > C_0$ μg/m³				
			C_0 = 5	C_0 = 10	C_0 = 15	C_0 = 25	C_0 = 35	C_0 = 5	C_0 = 10	C_0 = 15	C_0 = 25	C_0 = 35
ALL	2,089,918 (1,982,393, 2,200,882)	Age-specific curves	45.51 (29.24, 58.07)	39.66 (26.07, 50.85)	33.50 (22.77, 42.70)	22.05 (15.55, 27.71)	13.45 (8.60, 17.68)	951,060 (610,754, 1,234,209)	828,952 (543,961, 1,075,969)	700,224 (481,018, 899,490)	460,790 (317,459, 582,504)	281,112 (179,375, 367,804)
ALL	2,089,918 (1,982,393, 2,200,882)	All-ages curve	44.45 (26.36, 58.01)	38.59 (19.14, 53.53)	32.54 (13.21, 48.62)	21.77 (5.87, 38.41)	13.88 (2.60, 29.31)	928,901 (567,714, 1,220,350)	806,574 (413,107, 1,123,792)	680,031 (278,718, 1,021,294)	454,903 (121,082, 808,733)	290,087 (54,168, 620,742)
ALL	2,089,918 (1,982,393, 2,200,882)	Zhang et al. 2021	29.39 (21.81, 35.57)	25.93 (18.04, 32.40)	22.44 (14.50, 29.13)	16.18 (8.98, 22.79)	11.39 (5.40, 17.41)	614,306 (454,617, 750,733)	541,848 (376,134, 682,720)	469,028 (302,353, 614,199)	338,153 (187,357, 481,508)	237,965 (112,800, 368,306)
ALL	2,089,918 (1,982,393, 2,200,882)	Xie et al. 2021	38.15 (19.29, 52.98)	33.69 (14.13, 49.55)	29.22 (10.08, 45.91)	21.27 (5.02, 38.48)	15.26 (2.42, 31.62)	797,205 (403,038, 1,092,148)	704,081 (296,335, 1,023,507)	610,631 (211,441, 949,540)	444,582 (105,226, 794,868)	319,002 (50,827, 650,300)
AFG	37,252 (20,065, 54,486)	Age-specific curves	47.50 (29.36, 61.79)	41.84 (25.68, 55.15)	35.56 (21.79, 47.41)	21.69 (14.37, 29.04)	8.76 (5.85, 11.47)	17,693 (8,181, 27,782)	15,585 (7,211, 24,508)	13,248 (6,192, 20,953)	8,081 (3,889, 12,909)	3,262 (1,571, 5,322)
AFG	37,252 (20,065, 54,486)	All-ages curve	46.12 (27.00, 62.23)	40.23 (19.38, 58.19)	33.71 (11.73, 53.68)	20.06 (2.93, 43.43)	10.23 (0.82, 32.59)	17,181 (7,439, 27,604)	14,987 (5,803, 25,137)	12,557 (3,603, 22,768)	7,474 (903, 17,085)	3,810 (265, 12,113)
AFG	37,252 (20,065, 54,486)	Zhang et al. 2021	28.25 (20.58, 34.69)	24.68 (16.66, 31.48)	20.94 (12.59, 28.11)	13.15 (5.10, 20.92)	6.36 (1.36, 13.52)	10,523 (5,567, 16,151)	9,195 (4,668, 14,400)	7,801 (3,547, 12,832)	4,899 (1,636, 9,330)	2,370 (481, 5,859)
AFG	37,252 (20,065, 54,486)	Xie et al. 2021	37.44 (17.94, 53.60)	32.84 (11.99, 50.20)	27.91 (6.67, 46.54)	17.63 (1.67, 38.38)	9.72 (0.50, 29.45)	13,945 (5,010, 23,646)	12,235 (3,791, 21,678)	10,398 (2,214, 19,991)	6,569 (530, 15,768)	3,620 (174, 11,988)
AGO	25,458 (15,565, 35,943)	Age-specific curves	28.40 (15.50, 40.77)	20.61 (11.11, 30.19)	12.46 (6.65, 18.53)	1.88 (1.11, 2.71)	0.14 (0.09, 0.20)	7,229 (3,010, 12,400)	5,247 (2,181, 9,173)	3,173 (1,328, 5,627)	478 (233, 818)	36 (18, 61)
AGO	25,458 (15,565, 35,943)	All-ages curve	27.31 (11.80, 41.02)	19.38 (4.31, 34.78)	11.60 (1.25, 27.92)	3.39 (0.14, 14.98)	0.95 (0.02, 7.18)	6,952 (2,589, 12,240)	4,933 (913, 10,142)	2,954 (278, 7,899)	862 (38, 3,967)	243 (6, 1,778)
AGO	25,458 (15,565, 35,943)	Zhang et al. 2021	14.91 (10.02, 19.31)	10.67 (5.67, 15.36)	6.48 (2.12, 11.26)	1.27 (0.16, 4.09)	0.19 (0.01, 0.97)	3,797 (1,844, 5,748)	2,718 (1,109, 4,540)	1,649 (460, 3,200)	325 (38, 1,072)	47 (2, 251)

ISO	Total number of stillbirths	Method	Fraction of stillbirths attributable to $PM_{2.5} > C_0$ μg/m^3					Number of stillbirths attributable to $PM_{2.5} > C_0$ μg/m^3				
			$C_0 = 5$	$C_0 = 10$	$C_0 = 15$	$C_0 = 25$	$C_0 = 35$	$C_0 = 5$	$C_0 = 10$	$C_0 = 15$	$C_0 = 25$	$C_0 = 35$
AGO	25,458 (15,565, 35,943)	Xie et al. 2021	20.38 (7.46, 32.77)	14.53 (2.33, 27.90)	8.81 (0.65, 22.70)	2.59 (0.11, 12.48)	0.82 (0.04, 5.57)	5,188 (1,845, 9,186)	3,700 (622, 7,652)	2,244 (183, 5,824)	659 (28, 3,116)	210 (11, 1,345)
ARE	525 (488, 561)	Age-specific curves	64.63 (46.47, 76.45)	60.90 (44.22, 72.76)	56.77 (42.38, 67.86)	47.19 (34.14, 57.29)	35.90 (24.00, 45.39)	339 (246, 407)	320 (235, 385)	298 (221, 360)	248 (181, 304)	188 (124, 237)
ARE	525 (488, 561)	All-ages curve	60.06 (39.51, 73.11)	55.72 (33.00, 70.26)	50.88 (25.75, 67.08)	39.73 (11.47, 59.78)	27.55 (3.73, 51.53)	315 (208, 390)	293 (173, 374)	267 (134, 357)	209 (59, 317)	145 (19, 271)
ARE	525 (488, 561)	Zhang et al. 2021	42.77 (32.66, 50.78)	39.93 (29.34, 48.35)	36.95 (25.87, 45.80)	30.52 (18.37, 40.30)	23.43 (10.32, 34.24)	225 (169, 268)	210 (152, 255)	194 (135, 241)	160 (96, 212)	123 (54, 180)
ARE	525 (488, 561)	Xie et al. 2021	54.71 (30.07, 72.09)	51.40 (24.98, 70.05)	47.83 (19.50, 67.85)	39.83 (8.98, 62.91)	30.59 (3.25, 57.14)	287 (158, 376)	270 (132, 365)	251 (103, 352)	209 (48, 327)	161 (17, 297)
ARG	3,871 (3,760, 3,994)	Age-specific curves	17.38 (9.06, 26.20)	8.42 (4.34, 12.92)	2.42 (1.28, 3.74)	0.06 (0.04, 0.12)	0.00 (0.00, 0.00)	673 (345, 1,029)	326 (165, 507)	94 (49, 147)	2 (1, 5)	0 (0, 0)
ARG	3,871 (3,760, 3,994)	All-ages curve	17.06 (4.83, 27.87)	9.15 (0.78, 20.30)	3.67 (0.14, 12.80)	0.78 (0.01, 4.02)	0.29 (0.00, 1.44)	661 (186, 1,088)	354 (30, 789)	142 (5, 496)	30 (0, 156)	11 (0, 56)
ARG	3,871 (3,760, 3,994)	Zhang et al. 2021	8.82 (5.31, 11.94)	4.39 (1.48, 7.57)	1.45 (0.22, 3.65)	0.07 (0.00, 0.46)	0.00 (0.00, 0.04)	342 (200, 464)	170 (56, 295)	56 (8, 142)	3 (0, 18)	0 (0, 2)
ARG	3,871 (3,760, 3,994)	Xie et al. 2021	12.22 (3.00, 21.50)	6.33 (0.48, 15.70)	2.65 (0.10, 9.93)	0.58 (0.03, 3.20)	0.22 (0.01, 1.08)	473 (116, 823)	245 (19, 604)	103 (4, 389)	22 (1, 124)	8 (0, 42)
ARM	608 (562, 654)	Age-specific curves	41.87 (23.29, 56.85)	35.53 (19.68, 49.24)	28.50 (15.77, 40.27)	12.65 (7.03, 18.34)	1.11 (0.66, 1.60)	255 (138, 353)	216 (115, 306)	173 (93, 250)	77 (43, 113)	7 (4, 10)
ARM	608 (562, 654)	All-ages curve	40.92 (21.81, 56.60)	34.73 (13.45, 52.01)	27.85 (6.31, 46.88)	13.85 (0.55, 34.95)	5.52 (0.06, 22.88)	249 (133, 350)	211 (82, 320)	169 (39, 284)	84 (3, 214)	34 (0, 144)
ARM	608 (562, 654)	Zhang et al. 2021	23.48 (16.70, 29.31)	19.67 (12.59, 25.83)	15.67 (8.27, 22.17)	7.28 (1.44, 14.30)	1.48 (0.03, 6.66)	143 (99, 179)	120 (74, 158)	95 (49, 136)	44 (9, 85)	9 (0, 39)

ISO	Total number of stillbirths	Method	Fraction of stillbirths attributable to $PM_{2.5} > C_0$ μg/m^3					Number of stillbirths attributable to $PM_{2.5} > C_0$ μg/m^3				
			$C_0 = 5$	$C_0 = 10$	$C_0 = 15$	$C_0 = 25$	$C_0 = 35$	$C_0 = 5$	$C_0 = 10$	$C_0 = 15$	$C_0 = 25$	$C_0 = 35$
ARM	608 (562, 654)	Xie et al. 2021	31.58 (14.07, 46.97)	26.54 (7.85, 43.06)	21.10 (3.28, 38.79)	10.36 (0.35, 29.17)	3.70 (0.08, 18.93)	192 (84, 285)	161 (47, 261)	128 (20, 235)	63 (2, 179)	23 (0, 117)
ATG	9 (5, 12)	Age-specific curves	15.68 (7.80, 23.89)	6.64 (3.23, 10.41)	0.00 (0.00, 0.04)	0.00 (0.00, 0.06)	0.00 (0.00, 0.00)	1 (1, 2)	1 (0, 1)	0 (0, 0)	0 (0, 0)	0 (0, 0)
ATG	9 (5, 12)	All-ages curve	15.03 (3.54, 26.47)	6.24 (0.27, 18.85)	2.29 (0.00, 11.03)	0.01 (0.00, 3.11)	0.00 (0.00, 1.42)	1 (0, 3)	1 (0, 2)	0 (0, 1)	0 (0, 0)	0 (0, 0)
ATG	9 (5, 12)	Zhang et al. 2021	7.87 (4.64, 10.94)	3.24 (0.46, 6.60)	0.31 (0.00, 2.22)	0.00 (0.00, 0.01)	0.00 (0.00, 0.00)	1 (0, 1)	0 (0, 1)	0 (0, 0)	0 (0, 0)	0 (0, 0)
ATG	9 (5, 12)	Xie et al. 2021	10.61 (2.37, 19.91)	4.15 (0.19, 14.15)	0.97 (0.00, 8.34)	0.03 (0.00, 1.92)	0.01 (0.00, 0.84)	1 (0, 2)	0 (0, 1)	0 (0, 1)	0 (0, 0)	0 (0, 0)
AZE	1,833 (1,189, 2,464)	Age-specific curves	34.35 (17.86, 48.02)	27.19 (13.95, 38.88)	19.26 (9.83, 28.14)	2.91 (1.54, 4.38)	0.00 (0.00, 0.00)	630 (320, 1,027)	498 (250, 825)	353 (175, 592)	53 (26, 90)	0 (0, 0)
AZE	1,833 (1,189, 2,464)	All-ages curve	33.44 (15.65, 48.19)	26.32 (7.07, 42.68)	18.44 (1.90, 36.56)	5.92 (0.16, 23.06)	1.26 (0.00, 11.71)	613 (253, 1,018)	482 (119, 891)	338 (33, 759)	108 (3, 455)	23 (0, 218)
AZE	1,833 (1,189, 2,464)	Zhang et al. 2021	18.45 (12.69, 23.41)	14.44 (8.36, 19.62)	10.23 (3.93, 15.65)	2.29 (0.09, 7.27)	0.13 (0.00, 1.44)	338 (181, 501)	265 (117, 405)	188 (59, 304)	42 (2, 138)	2 (0, 26)
AZE	1,833 (1,189, 2,464)	Xie et al. 2021	25.10 (10.10, 38.88)	19.64 (3.88, 34.39)	13.81 (0.98, 29.54)	4.43 (0.14, 18.81)	0.96 (0.04, 9.26)	460 (161, 779)	360 (69, 678)	253 (18, 569)	81 (2, 360)	18 (1, 179)
BDI	11,040 (9,749, 12,453)	Age-specific curves	47.36 (27.66, 62.82)	41.64 (23.99, 56.12)	35.28 (20.17, 48.20)	20.62 (12.07, 28.91)	4.19 (2.62, 5.85)	5,229 (3,045, 6,832)	4,597 (2,646, 6,096)	3,894 (2,246, 5,230)	2,276 (1,345, 3,150)	463 (294, 634)
BDI	11,040 (9,749, 12,453)	All-ages curve	45.61 (25.89, 61.84)	39.74 (17.69, 57.73)	33.19 (9.09, 53.16)	18.76 (1.38, 42.70)	8.50 (0.23, 31.10)	5,035 (2,696, 7,083)	4,387 (1,883, 6,688)	3,664 (1,017, 6,194)	2,071 (152, 4,988)	938 (24, 3,661)
BDI	11,040 (9,749, 12,453)	Zhang et al. 2021	26.99 (19.53, 33.39)	23.30 (15.56, 30.10)	19.43 (11.39, 26.64)	11.07 (3.02, 19.24)	3.22 (0.15, 11.12)	2,979 (2,047, 3,759)	2,573 (1,643, 3,376)	2,145 (1,217, 2,977)	1,222 (335, 2,120)	356 (17, 1,228)

ISO	Total number of stillbirths	Method	Fraction of stillbirths attributable to $PM_{2.5} > C_0$ μg/m^3					Number of stillbirths attributable to $PM_{2.5} > C_0$ μg/m^3				
			$C_0 = 5$	$C_0 = 10$	$C_0 = 15$	$C_0 = 25$	$C_0 = 35$	$C_0 = 5$	$C_0 = 10$	$C_0 = 15$	$C_0 = 25$	$C_0 = 35$
BDI	11,040 (9,749, 12,453)	Xie et al. 2021	36.18 (17.02, 52.40)	31.58 (11.03, 48.90)	26.62 (5.42, 45.12)	15.58 (0.89, 36.77)	6.74 (0.22, 27.06)	3,994 (1,806, 5,902)	3,486 (1,170, 5,425)	2,939 (575, 4,943)	1,720 (100, 3,998)	744 (23, 2,859)
BEN	8,629 (8,196, 9,072)	Age-specific curves	50.58 (31.64, 64.80)	45.25 (28.14, 58.74)	39.34 (25.08, 51.62)	25.79 (17.28, 34.06)	10.90 (7.01, 14.02)	4,364 (2,766, 5,616)	3,905 (2,466, 5,087)	3,395 (2,141, 4,446)	2,226 (1,488, 2,943)	940 (609, 1,222)
BEN	8,629 (8,196, 9,072)	All-ages curve	49.20 (29.24, 64.70)	43.81 (21.87, 60.88)	37.82 (13.92, 56.63)	24.31 (3.76, 46.87)	12.59 (1.11, 35.89)	4,245 (2,525, 5,598)	3,780 (1,880, 5,256)	3,263 (1,196, 4,881)	2,098 (338, 4,019)	1,086 (100, 3,112)
BEN	8,629 (8,196, 9,072)	Zhang et al. 2021	30.79 (22.62, 37.54)	27.35 (18.80, 34.44)	23.74 (14.79, 31.18)	15.94 (6.60, 24.21)	8.51 (2.46, 16.55)	2,657 (1,966, 3,292)	2,360 (1,639, 3,029)	2,048 (1,295, 2,740)	1,375 (580, 2,125)	735 (217, 1,443)
BEN	8,629 (8,196, 9,072)	Xie et al. 2021	40.56 (20.13, 57.17)	36.18 (14.36, 54.01)	31.44 (8.59, 50.60)	21.19 (2.55, 42.94)	12.76 (0.79, 34.02)	3,500 (1,698, 4,895)	3,122 (1,221, 4,619)	2,713 (738, 4,313)	1,828 (216, 3,655)	1,101 (68, 2,918)
BFA	14,744 (14,033, 15,410)	Age-specific curves	53.03 (33.37, 67.30)	47.95 (30.31, 61.67)	42.31 (27.39, 55.04)	29.37 (19.66, 38.70)	14.80 (9.93, 19.30)	7,818 (4,916, 9,870)	7,069 (4,494, 8,989)	6,238 (4,061, 8,016)	4,330 (2,906, 5,694)	2,182 (1,444, 2,895)
BFA	14,744 (14,033, 15,410)	All-ages curve	51.24 (30.56, 66.72)	46.03 (23.13, 63.14)	40.23 (15.02, 59.14)	27.09 (3.97, 49.98)	15.46 (1.04, 39.71)	7,555 (4,523, 9,792)	6,787 (3,431, 9,266)	5,931 (2,229, 8,680)	3,994 (589, 7,332)	2,279 (154, 5,840)
BFA	14,744 (14,033, 15,410)	Zhang et al. 2021	32.26 (23.79, 39.24)	28.92 (20.03, 36.23)	25.40 (16.07, 33.06)	17.83 (7.77, 26.25)	9.86 (1.90, 18.74)	4,757 (3,477, 5,819)	4,263 (2,928, 5,377)	3,745 (2,349, 4,922)	2,628 (1,134, 3,931)	1,454 (277, 2,808)
BFA	14,744 (14,033, 15,410)	Xie et al. 2021	42.45 (21.16, 59.47)	38.24 (15.36, 56.50)	33.70 (9.39, 53.29)	23.58 (2.21, 46.08)	13.69 (0.60, 37.67)	6,259 (3,129, 8,794)	5,638 (2,275, 8,350)	4,969 (1,391, 7,853)	3,476 (328, 6,780)	2,018 (87, 5,535)
BGD	82,980 (72,235, 92,339)	Age-specific curves	62.81 (45.63, 75.08)	58.77 (43.14, 70.56)	54.29 (39.89, 65.92)	44.11 (28.47, 55.94)	32.77 (19.15, 45.31)	52,116 (37,874, 64,287)	48,765 (35,430, 60,640)	45,052 (31,353, 56,173)	36,606 (24,443, 47,290)	27,193 (16,380, 38,385)
BGD	82,980 (72,235, 92,339)	All-ages curve	62.37 (42.41, 75.12)	58.35 (36.20, 72.45)	53.87 (29.33, 69.46)	43.58 (15.22, 62.61)	32.17 (6.30, 54.89)	51,755 (35,835, 65,266)	48,418 (30,457, 62,410)	44,701 (24,623, 59,212)	36,165 (12,947, 52,100)	26,696 (5,402, 45,930)
BGD	82,980 (72,235, 92,339)	Zhang et al. 2021	46.62 (36.01, 54.84)	43.99 (32.85, 52.61)	41.22 (29.53, 50.26)	35.25 (22.38, 45.21)	28.68 (14.75, 39.64)	38,689 (28,707, 48,483)	36,501 (26,298, 46,289)	34,203 (23,741, 43,751)	29,254 (17,901, 38,933)	23,795 (11,806, 34,205)

ISO	Total number of stillbirths	Method	Fraction of stillbirths attributable to $PM_{2.5} > C_0$ μg/m^3					Number of stillbirths attributable to $PM_{2.5} > C_0$ μg/m^3				
			$C_0 = 5$	$C_0 = 10$	$C_0 = 15$	$C_0 = 25$	$C_0 = 35$	$C_0 = 5$	$C_0 = 10$	$C_0 = 15$	$C_0 = 25$	$C_0 = 35$
BGD	82,980 (72,235, 92,339)	Xie et al. 2021	58.93 (33.38, 75.94)	55.94 (28.48, 74.18)	52.71 (23.21, 72.28)	45.46 (12.46, 68.02)	37.14 (5.13, 63.05)	48,902 (26,428, 64,087)	46,418 (22,540, 62,330)	43,739 (18,330, 60,453)	37,726 (9,855, 56,821)	30,820 (4,060, 52,875)
BHR	127 (117, 137)	Age-specific curves	61.50 (41.98, 73.95)	57.44 (39.26, 69.85)	52.93 (36.86, 65.21)	42.54 (30.67, 52.69)	30.34 (19.68, 38.45)	78 (53, 95)	73 (50, 90)	67 (46, 83)	54 (37, 68)	39 (26, 49)
BHR	127 (117, 137)	All-ages curve	58.23 (36.22, 71.48)	53.94 (29.27, 68.41)	49.16 (21.62, 64.99)	38.23 (8.37, 57.15)	26.15 (2.43, 48.38)	74 (46, 91)	68 (37, 87)	62 (27, 83)	49 (11, 73)	33 (3, 62)
BHR	127 (117, 137)	Zhang et al. 2021	39.63 (29.93, 47.44)	36.61 (26.46, 44.85)	33.45 (22.81, 42.14)	26.62 (14.94, 36.31)	19.08 (6.62, 29.93)	50 (37, 62)	46 (33, 58)	42 (29, 55)	34 (19, 47)	24 (9, 38)
BHR	127 (117, 137)	Xie et al. 2021	51.16 (27.36, 68.79)	47.52 (22.08, 66.50)	43.59 (16.39, 64.02)	34.73 (5.84, 58.45)	24.69 (1.52, 51.94)	65 (35, 89)	60 (28, 86)	55 (21, 82)	44 (7, 74)	31 (2, 66)
BHS	65 (59, 72)	Age-specific curves	1.34 (0.67, 2.14)	0.00 (0.00, 0.11)	0.00 (0.00, 0.23)	0.00 (0.00, 0.29)	0.00 (0.00, 0.00)	1 (0, 1)	0 (0, 0)	0 (0, 0)	0 (0, 0)	0 (0, 0)
BHS	65 (59, 72)	All-ages curve	2.33 (0.02, 6.94)	0.02 (0.00, 1.24)	0.00 (0.00, 0.40)	0.00 (0.00, 0.04)	0.00 (0.00, 0.00)	2 (0, 5)	0 (0, 1)	0 (0, 0)	0 (0, 0)	0 (0, 0)
BHS	65 (59, 72)	Zhang et al. 2021	1.04 (0.05, 2.08)	0.00 (0.00, 0.03)	0.00 (0.00, 0.00)	0.00 (0.00, 0.00)	0.00 (0.00, 0.00)	1 (0, 1)	0 (0, 0)	0 (0, 0)	0 (0, 0)	0 (0, 0)
BHS	65 (59, 72)	Xie et al. 2021	1.51 (0.02, 5.02)	0.03 (0.00, 1.01)	0.00 (0.00, 0.38)	0.00 (0.00, 0.13)	0.00 (0.00, 0.02)	1 (0, 3)	0 (0, 1)	0 (0, 0)	0 (0, 0)	0 (0, 0)
BLZ	62 (56, 68)	Age-specific curves	22.19 (11.40, 32.74)	13.78 (6.95, 20.83)	4.95 (2.47, 7.63)	0.00 (0.00, 0.01)	0.00 (0.00, 0.00)	14 (7, 21)	9 (4, 13)	3 (2, 5)	0 (0, 0)	0 (0, 0)
BLZ	62 (56, 68)	All-ages curve	21.65 (7.42, 34.21)	13.31 (1.28, 27.42)	5.90 (0.18, 20.18)	0.76 (0.00, 7.64)	0.07 (0.00, 2.52)	13 (5, 22)	8 (1, 18)	4 (0, 13)	0 (0, 5)	0 (0, 2)
BLZ	62 (56, 68)	Zhang et al. 2021	11.38 (7.29, 15.07)	6.97 (2.79, 10.88)	2.67 (0.27, 6.57)	0.04 (0.00, 0.69)	0.00 (0.00, 0.02)	7 (4, 10)	4 (2, 7)	2 (0, 4)	0 (0, 0)	0 (0, 0)

ISO	Total number of stillbirths	Method	Fraction of stillbirths attributable to $PM_{2.5} > C_0$ μg/m^3					Number of stillbirths attributable to $PM_{2.5} > C_0$ μg/m^3				
			$C_0 = 5$	$C_0 = 10$	$C_0 = 15$	$C_0 = 25$	$C_0 = 35$	$C_0 = 5$	$C_0 = 10$	$C_0 = 15$	$C_0 = 25$	$C_0 = 35$
BLZ	62 (56, 68)	Xie et al. 2021	15.65 (4.73, 26.43)	9.44 (0.72, 21.00)	4.26 (0.07, 15.28)	0.47 (0.01, 5.67)	0.24 (0.00, 1.78)	10 (3, 17)	6 (0, 13)	3 (0, 10)	0 (0, 4)	0 (0, 1)
BOL	2,525 (1,822, 3,212)	Age-specific curves	36.11 (20.05, 50.56)	29.19 (16.11, 41.63)	21.53 (11.80, 31.08)	6.92 (3.89, 9.92)	0.40 (0.25, 0.57)	912 (420, 1,381)	737 (337, 1,138)	544 (249, 850)	175 (87, 275)	10 (5, 16)
BOL	2,525 (1,822, 3,212)	All-ages curve	35.06 (17.33, 49.89)	28.10 (9.16, 44.50)	20.59 (3.83, 38.56)	8.12 (0.50, 25.67)	2.22 (0.08, 15.01)	885 (360, 1,425)	710 (204, 1,267)	520 (85, 1,079)	205 (13, 734)	56 (2, 426)
BOL	2,525 (1,822, 3,212)	Zhang et al. 2021	19.68 (13.68, 24.86)	15.70 (9.40, 21.16)	11.53 (5.23, 17.27)	4.18 (0.61, 9.31)	0.82 (0.01, 3.47)	497 (301, 690)	396 (208, 575)	291 (118, 463)	106 (14, 250)	21 (0, 93)
BOL	2,525 (1,822, 3,212)	Xie et al. 2021	26.66 (11.20, 40.75)	21.30 (5.39, 36.39)	15.64 (1.88, 31.68)	6.28 (0.25, 21.56)	1.96 (0.08, 12.49)	673 (213, 1,141)	538 (103, 1,005)	395 (40, 858)	158 (6, 554)	49 (2, 314)
BRA	24,389 (22,345, 26,404)	Age-specific curves	17.15 (8.60, 25.92)	8.88 (4.47, 13.56)	3.63 (1.94, 5.52)	0.55 (0.34, 0.85)	0.02 (0.01, 0.02)	4,182 (2,048, 6,369)	2,165 (1,060, 3,329)	886 (450, 1,355)	134 (84, 205)	4 (2, 6)
BRA	24,389 (22,345, 26,404)	All-ages curve	17.03 (5.29, 27.98)	9.51 (1.35, 20.54)	4.45 (0.37, 13.54)	1.08 (0.05, 5.26)	0.32 (0.00, 2.24)	4,154 (1,303, 6,906)	2,320 (332, 5,048)	1,084 (90, 3,392)	264 (11, 1,289)	77 (1, 532)
BRA	24,389 (22,345, 26,404)	Zhang et al. 2021	8.98 (5.46, 12.13)	4.78 (1.94, 7.85)	2.11 (0.56, 4.31)	0.37 (0.04, 1.03)	0.05 (0.00, 0.28)	2,190 (1,280, 2,977)	1,165 (455, 1,940)	515 (134, 1,081)	90 (9, 263)	12 (0, 69)
BRA	24,389 (22,345, 26,404)	Xie et al. 2021	12.45 (3.43, 21.69)	6.79 (0.80, 16.01)	3.32 (0.21, 10.86)	0.72 (0.03, 4.45)	0.27 (0.01, 1.77)	3,036 (797, 5,134)	1,655 (189, 3,800)	811 (52, 2,597)	175 (7, 1,038)	65 (1, 412)
BRB	25 (15, 36)	Age-specific curves	20.32 (10.48, 30.63)	11.72 (5.93, 18.15)	2.45 (1.23, 3.88)	0.00 (0.00, 0.01)	0.00 (0.00, 0.00)	5 (2, 8)	3 (1, 5)	1 (0, 1)	0 (0, 0)	0 (0, 0)
BRB	25 (15, 36)	All-ages curve	19.67 (6.04, 32.42)	10.97 (0.61, 25.54)	3.72 (0.04, 17.75)	0.30 (0.00, 5.26)	0.00 (0.00, 1.36)	5 (1, 10)	3 (0, 8)	1 (0, 5)	0 (0, 1)	0 (0, 0)
BRB	25 (15, 36)	Zhang et al. 2021	10.34 (6.46, 13.81)	5.88 (1.92, 9.59)	1.66 (0.06, 5.18)	0.00 (0.00, 0.25)	0.00 (0.00, 0.00)	3 (1, 4)	1 (0, 3)	0 (0, 1)	0 (0, 0)	0 (0, 0)

ISO	Total number of stillbirths	Method	Fraction of stillbirths attributable to $PM_{2.5} > C_0$ μg/m^3					Number of stillbirths attributable to $PM_{2.5} > C_0$ μg/m^3				
			$C_0 = 5$	$C_0 = 10$	$C_0 = 15$	$C_0 = 25$	$C_0 = 35$	$C_0 = 5$	$C_0 = 10$	$C_0 = 15$	$C_0 = 25$	$C_0 = 35$
BRB	25 (15, 36)	Xie et al. 2021	14.59 (3.96, 24.72)	8.64 (0.40, 19.33)	3.74 (0.04, 13.52)	0.24 (0.00, 4.07)	0.11 (0.00, 1.12)	4 (1, 7)	2 (0, 5)	1 (0, 4)	0 (0, 1)	0 (0, 0)
BRN	30 (26, 34)	Age-specific curves	14.01 (6.98, 22.18)	4.71 (2.30, 7.69)	0.00 (0.00, 0.07)	0.00 (0.00, 0.06)	0.00 (0.00, 0.00)	4 (2, 7)	1 (1, 2)	0 (0, 0)	0 (0, 0)	0 (0, 0)
BRN	30 (26, 34)	All-ages curve	13.59 (2.81, 24.19)	5.07 (0.10, 16.66)	1.18 (0.00, 8.87)	0.07 (0.00, 1.84)	0.00 (0.00, 0.84)	4 (1, 7)	2 (0, 5)	0 (0, 3)	0 (0, 1)	0 (0, 0)
BRN	30 (26, 34)	Zhang et al. 2021	7.21 (3.91, 9.75)	2.89 (0.21, 5.28)	0.32 (0.00, 1.22)	0.00 (0.00, 0.00)	0.00 (0.00, 0.00)	2 (1, 3)	1 (0, 2)	0 (0, 0)	0 (0, 0)	0 (0, 0)
BRN	30 (26, 34)	Xie et al. 2021	9.96 (1.86, 18.26)	3.94 (0.04, 12.64)	0.69 (0.00, 6.82)	0.08 (0.00, 1.42)	0.00 (0.00, 0.67)	3 (1, 5)	1 (0, 4)	0 (0, 2)	0 (0, 0)	0 (0, 0)
BTN	135 (113, 157)	Age-specific curves	36.13 (21.08, 49.32)	29.23 (17.06, 40.35)	21.65 (12.66, 30.07)	9.36 (6.00, 12.67)	3.49 (2.25, 4.56)	49 (26, 68)	39 (22, 56)	29 (16, 41)	13 (8, 17)	5 (3, 7)
BTN	135 (113, 157)	All-ages curve	35.32 (18.37, 49.84)	28.51 (10.70, 44.48)	21.45 (5.54, 38.53)	10.62 (1.24, 26.47)	5.07 (0.28, 16.92)	48 (23, 70)	38 (14, 62)	29 (7, 53)	14 (2, 36)	7 (0, 23)
BTN	135 (113, 157)	Zhang et al. 2021	20.43 (14.33, 25.60)	16.52 (10.11, 21.93)	12.48 (6.30, 18.08)	6.04 (2.11, 10.75)	2.74 (0.64, 5.82)	28 (18, 36)	22 (13, 31)	17 (8, 25)	8 (3, 14)	4 (1, 8)
BTN	135 (113, 157)	Xie et al. 2021	27.48 (11.78, 41.31)	22.25 (6.29, 37.00)	16.95 (3.16, 32.37)	8.99 (0.80, 22.80)	4.66 (0.24, 14.98)	37 (15, 58)	30 (8, 51)	23 (4, 44)	12 (1, 31)	6 (0, 20)
BWA	902 (833, 982)	Age-specific curves	22.71 (11.75, 33.58)	14.37 (7.34, 21.73)	5.30 (2.75, 8.09)	0.29 (0.17, 0.44)	0.00 (0.00, 0.00)	205 (108, 303)	130 (67, 196)	48 (25, 73)	3 (2, 4)	0 (0, 0)
BWA	902 (833, 982)	All-ages curve	21.83 (8.05, 34.75)	13.25 (1.86, 28.02)	6.21 (0.40, 20.67)	1.06 (0.03, 8.49)	0.22 (0.00, 2.89)	197 (71, 323)	120 (16, 260)	56 (4, 190)	10 (0, 80)	2 (0, 27)
BWA	902 (833, 982)	Zhang et al. 2021	11.73 (7.52, 15.53)	7.37 (3.04, 11.37)	3.10 (0.66, 6.94)	0.26 (0.01, 1.23)	0.02 (0.00, 0.15)	106 (67, 139)	66 (28, 101)	28 (6, 62)	2 (0, 11)	0 (0, 1)

ISO	Total number of stillbirths	Method	Fraction of stillbirths attributable to $PM_{2.5} > C_0$ μg/m^3					Number of stillbirths attributable to $PM_{2.5} > C_0$ μg/m^3				
			$C_0 = 5$	$C_0 = 10$	$C_0 = 15$	$C_0 = 25$	$C_0 = 35$	$C_0 = 5$	$C_0 = 10$	$C_0 = 15$	$C_0 = 25$	$C_0 = 35$
BWA	902 (833, 982)	Xie et al. 2021	16.10 (5.01, 26.95)	9.94 (1.06, 21.51)	4.94 (0.18, 15.79)	0.96 (0.03, 6.41)	0.39 (0.01, 2.29)	145 (45, 242)	90 (10, 194)	45 (2, 143)	9 (0, 58)	4 (0, 21)
CAF	5,374 (3,216, 7,599)	Age-specific curves	44.62 (25.85, 59.95)	38.66 (22.12, 52.79)	32.05 (18.26, 44.35)	16.88 (10.07, 23.72)	2.35 (1.54, 3.28)	2,398 (1,160, 3,711)	2,078 (1,006, 3,222)	1,722 (843, 2,683)	907 (463, 1,418)	126 (69, 200)
CAF	5,374 (3,216, 7,599)	All-ages curve	43.53 (24.08, 59.65)	37.53 (15.76, 55.37)	30.85 (7.70, 50.60)	16.44 (1.15, 39.65)	6.52 (0.19, 27.74)	2,339 (1,018, 3,828)	2,017 (682, 3,470)	1,658 (370, 3,123)	883 (58, 2,321)	350 (11, 1,533)
CAF	5,374 (3,216, 7,599)	Zhang et al. 2021	25.44 (18.22, 31.52)	21.77 (14.17, 28.14)	17.92 (9.93, 24.58)	9.63 (2.07, 16.92)	2.37 (0.09, 8.71)	1,367 (785, 2,091)	1,170 (621, 1,853)	963 (449, 1,583)	518 (94, 1,074)	127 (4, 488)
CAF	5,374 (3,216, 7,599)	Xie et al. 2021	34.10 (15.67, 49.99)	29.32 (9.54, 46.34)	24.16 (4.11, 42.40)	13.10 (0.54, 33.56)	5.52 (0.14, 23.42)	1,833 (743, 3,176)	1,576 (490, 2,955)	1,298 (229, 2,631)	704 (29, 2,090)	297 (8, 1,361)
CHL	776 (744, 806)	Age-specific curves	30.06 (16.16, 43.32)	22.55 (12.01, 33.14)	14.62 (7.84, 21.77)	2.69 (1.50, 4.02)	0.05 (0.03, 0.07)	233 (126, 334)	175 (93, 255)	113 (61, 167)	21 (12, 31)	0 (0, 1)
CHL	776 (744, 806)	All-ages curve	29.57 (13.00, 43.26)	22.33 (5.33, 37.29)	15.18 (1.58, 30.75)	5.37 (0.16, 18.13)	1.78 (0.02, 8.97)	229 (101, 340)	173 (42, 294)	118 (12, 242)	42 (1, 142)	14 (0, 69)
CHL	776 (744, 806)	Zhang et al. 2021	16.07 (10.87, 20.57)	11.92 (6.53, 16.60)	7.85 (2.98, 12.52)	1.81 (0.13, 5.43)	0.11 (0.00, 1.24)	125 (83, 161)	92 (50, 131)	61 (23, 99)	14 (1, 43)	1 (0, 10)
CHL	776 (744, 806)	Xie et al. 2021	21.80 (8.32, 34.58)	15.98 (2.93, 29.72)	10.53 (0.70, 24.50)	3.29 (0.10, 14.58)	0.95 (0.04, 7.37)	169 (63, 268)	124 (22, 231)	82 (5, 191)	25 (1, 114)	7 (0, 56)
CHN	130,583 (127,001, 134,144)	Age-specific curves	53.85 (35.31, 67.14)	48.88 (32.79, 61.38)	43.38 (29.16, 54.58)	31.04 (22.04, 39.31)	19.00 (11.89, 24.81)	70,314 (46,070, 87,987)	63,830 (42,315, 80,717)	56,648 (37,606, 71,794)	40,533 (28,461, 51,434)	24,810 (15,419, 32,548)
CHN	130,583 (127,001, 134,144)	All-ages curve	52.41 (31.90, 66.02)	47.31 (24.72, 62.41)	41.66 (17.57, 58.41)	29.47 (7.44, 49.28)	18.88 (2.78, 39.40)	68,438 (41,127, 86,537)	61,781 (31,870, 81,662)	54,395 (22,654, 76,478)	38,488 (9,550, 64,219)	24,651 (3,611, 51,306)
CHN	130,583 (127,001, 134,144)	Zhang et al. 2021	35.36 (26.55, 42.46)	32.18 (22.94, 39.62)	28.85 (19.17, 36.65)	21.82 (11.85, 30.29)	15.16 (6.47, 23.62)	46,179 (34,354, 55,761)	42,025 (29,717, 52,061)	37,668 (24,807, 48,154)	28,488 (15,335, 39,692)	19,802 (8,378, 30,973)

ISO	Total number of stillbirths	Method	Fraction of stillbirths attributable to $PM_{2.5} > C_0$ μg/m^3					Number of stillbirths attributable to $PM_{2.5} > C_0$ μg/m^3				
			C_0 = 5	C_0 = 10	C_0 = 15	C_0 = 25	C_0 = 35	C_0 = 5	C_0 = 10	C_0 = 15	C_0 = 25	C_0 = 35
CHN	130,583 (127,001, 134,144)	Xie et al. 2021	45.72 (23.98, 62.17)	41.77 (18.52, 59.41)	37.51 (13.29, 56.44)	28.46 (5.96, 49.81)	20.19 (2.51, 42.41)	59,705 (31,295, 80,600)	54,539 (24,171, 76,986)	48,987 (17,344, 73,091)	37,164 (7,776, 64,391)	26,367 (3,271, 54,705)
CIV	21,547 (12,883, 31,098)	Age-specific curves	35.71 (19.84, 50.08)	28.74 (15.83, 41.20)	21.00 (11.43, 30.76)	3.72 (2.08, 5.51)	0.02 (0.01, 0.03)	7,695 (3,398, 12,771)	6,193 (2,726, 10,467)	4,525 (1,983, 7,775)	801 (348, 1,379)	5 (2, 8)
CIV	21,547 (12,883, 31,098)	All-ages curve	34.55 (16.79, 49.74)	27.40 (8.01, 44.49)	19.45 (2.60, 38.67)	5.51 (0.28, 25.42)	1.04 (0.02, 13.65)	7,444 (2,666, 12,739)	5,904 (1,505, 11,332)	4,192 (537, 9,826)	1,188 (53, 5,770)	223 (4, 2,824)
CIV	21,547 (12,883, 31,098)	Zhang et al. 2021	19.22 (13.30, 24.33)	15.22 (9.00, 20.58)	11.02 (4.49, 16.64)	2.99 (0.16, 8.22)	0.31 (0.00, 1.79)	4,142 (2,000, 6,434)	3,280 (1,389, 5,227)	2,374 (783, 4,046)	644 (30, 1,888)	66 (0, 362)
CIV	21,547 (12,883, 31,098)	Xie et al. 2021	25.88 (10.82, 40.19)	20.20 (4.68, 35.74)	14.05 (1.27, 30.94)	4.42 (0.12, 20.18)	1.36 (0.04, 10.37)	5,577 (1,960, 10,731)	4,352 (1,004, 9,347)	3,027 (264, 7,970)	953 (24, 5,036)	293 (7, 2,463)
CMR	18,096 (10,512, 26,364)	Age-specific curves	58.74 (42.38, 71.44)	54.33 (39.23, 66.66)	49.44 (35.72, 60.74)	38.25 (27.38, 47.34)	25.67 (16.49, 33.18)	10,630 (5,691, 16,068)	9,832 (5,336, 14,914)	8,947 (5,009, 13,504)	6,922 (4,025, 10,417)	4,645 (2,598, 7,296)
CMR	18,096 (10,512, 26,364)	All-ages curve	57.11 (36.21, 70.44)	52.50 (29.35, 67.27)	47.35 (22.28, 63.74)	35.62 (9.92, 55.64)	24.07 (4.17, 46.51)	10,335 (5,111, 15,297)	9,500 (4,491, 14,442)	8,568 (3,484, 13,393)	6,447 (1,531, 11,345)	4,355 (590, 9,623)
CMR	18,096 (10,512, 26,364)	Zhang et al. 2021	40.07 (30.48, 47.68)	37.11 (27.05, 45.09)	34.00 (23.45, 42.38)	27.29 (15.82, 36.52)	20.13 (9.10, 30.07)	7,251 (3,891, 10,294)	6,715 (3,609, 9,626)	6,152 (3,192, 8,900)	4,939 (2,221, 7,517)	3,643 (1,408, 5,913)
CMR	18,096 (10,512, 26,364)	Xie et al. 2021	51.35 (27.89, 68.25)	47.81 (22.64, 65.93)	43.98 (17.12, 63.42)	35.44 (8.14, 57.79)	26.66 (3.49, 51.25)	9,293 (3,882, 15,085)	8,651 (3,119, 14,409)	7,959 (2,462, 13,651)	6,414 (1,251, 12,123)	4,825 (533, 10,263)
COD	95,939 (80,216, 111,260)	Age-specific curves	48.65 (29.04, 63.61)	43.11 (25.57, 57.08)	36.96 (21.91, 49.41)	22.84 (14.48, 30.83)	8.86 (5.94, 11.70)	46,678 (27,302, 62,092)	41,360 (24,226, 55,524)	35,458 (21,138, 48,000)	21,909 (13,449, 29,873)	8,502 (5,437, 11,267)
COD	95,939 (80,216, 111,260)	All-ages curve	47.06 (27.37, 63.27)	41.41 (19.64, 59.33)	35.13 (11.56, 54.95)	21.41 (2.43, 44.89)	10.27 (0.51, 33.91)	45,147 (24,889, 64,003)	39,733 (18,124, 58,787)	33,706 (10,684, 53,268)	20,544 (2,229, 42,586)	9,854 (465, 30,591)
COD	95,939 (80,216, 111,260)	Zhang et al. 2021	28.50 (20.73, 35.01)	24.95 (16.81, 31.81)	21.22 (12.70, 28.44)	13.24 (4.74, 21.18)	5.83 (0.71, 13.49)	27,338 (18,681, 34,490)	23,934 (15,082, 30,771)	20,357 (11,182, 27,272)	12,700 (4,269, 20,279)	5,594 (695, 12,825)

ISO	Total number of stillbirths	Method	Fraction of stillbirths attributable to $PM_{2.5} > C_0$ μg/m^3					Number of stillbirths attributable to $PM_{2.5} > C_0$ μg/m^3				
			$C_0 = 5$	$C_0 = 10$	$C_0 = 15$	$C_0 = 25$	$C_0 = 35$	$C_0 = 5$	$C_0 = 10$	$C_0 = 15$	$C_0 = 25$	$C_0 = 35$
COD	95,939 (80,216, 111,260)	Xie et al. 2021	37.87 (18.16, 54.27)	33.35 (12.22, 50.92)	28.48 (6.67, 47.30)	18.07 (1.41, 39.19)	9.22 (0.34, 29.99)	36,335 (16,922, 52,607)	31,999 (11,178, 49,004)	27,322 (6,014, 45,420)	17,336 (1,270, 37,628)	8,849 (317, 28,026)
COG	2,653 (1,564, 3,750)	Age-specific curves	44.17 (26.15, 59.15)	38.13 (22.38, 51.74)	31.43 (18.47, 42.96)	17.25 (10.82, 23.59)	6.09 (4.02, 8.28)	1,172 (585, 1,865)	1,012 (502, 1,624)	834 (415, 1,342)	458 (247, 738)	162 (90, 261)
COG	2,653 (1,564, 3,750)	All-ages curve	42.75 (24.01, 58.47)	36.74 (16.13, 54.05)	30.09 (9.04, 49.15)	16.71 (1.74, 38.13)	7.66 (0.23, 27.51)	1,134 (498, 1,821)	975 (350, 1,656)	798 (196, 1,505)	443 (41, 1,078)	203 (6, 724)
COG	2,653 (1,564, 3,750)	Zhang et al. 2021	25.21 (18.08, 31.25)	21.48 (14.03, 27.86)	17.57 (9.84, 24.29)	9.78 (3.13, 16.66)	3.73 (0.24, 9.73)	669 (378, 997)	570 (311, 873)	466 (219, 748)	260 (79, 490)	99 (6, 273)
COG	2,653 (1,564, 3,750)	Xie et al. 2021	33.63 (15.48, 49.32)	28.68 (9.46, 45.63)	23.34 (4.63, 41.64)	13.14 (0.76, 32.80)	5.72 (0.15, 23.69)	892 (340, 1,592)	761 (226, 1,448)	619 (108, 1,303)	349 (17, 957)	152 (4, 667)
COL	5,718 (4,340, 7,020)	Age-specific curves	24.35 (12.60, 35.87)	16.22 (8.26, 24.47)	7.44 (3.77, 11.43)	0.09 (0.05, 0.14)	0.00 (0.00, 0.00)	1,393 (733, 2,168)	928 (486, 1,475)	425 (222, 686)	5 (3, 9)	0 (0, 0)
COL	5,718 (4,340, 7,020)	All-ages curve	23.57 (9.12, 36.83)	15.13 (2.29, 30.19)	7.42 (0.41, 22.88)	1.30 (0.02, 9.85)	0.27 (0.00, 3.56)	1,348 (528, 2,268)	865 (138, 1,835)	424 (26, 1,385)	74 (1, 561)	15 (0, 183)
COL	5,718 (4,340, 7,020)	Zhang et al. 2021	12.65 (8.27, 16.62)	8.32 (3.80, 12.52)	3.97 (0.76, 8.19)	0.23 (0.00, 1.51)	0.01 (0.00, 0.10)	724 (422, 1,000)	476 (205, 734)	227 (41, 462)	13 (0, 79)	0 (0, 6)
COL	5,718 (4,340, 7,020)	Xie et al. 2021	17.41 (5.73, 28.77)	11.39 (1.25, 23.52)	6.02 (0.23, 17.91)	0.93 (0.03, 7.76)	0.37 (0.01, 2.82)	996 (319, 1,720)	651 (68, 1,376)	344 (13, 1,062)	53 (2, 452)	21 (0, 157)
COM	692 (409, 991)	Age-specific curves	11.79 (6.03, 18.42)	2.19 (1.10, 3.53)	0.00 (0.00, 0.10)	0.00 (0.00, 0.15)	0.00 (0.00, 0.00)	82 (32, 136)	15 (6, 26)	0 (0, 1)	0 (0, 1)	0 (0, 0)
COM	692 (409, 991)	All-ages curve	11.50 (1.66, 21.17)	3.22 (0.03, 12.76)	0.52 (0.00, 5.97)	0.00 (0.00, 1.30)	0.00 (0.00, 0.41)	80 (13, 162)	22 (0, 90)	4 (0, 39)	0 (0, 8)	0 (0, 3)
COM	692 (409, 991)	Zhang et al. 2021	5.96 (3.05, 8.50)	1.50 (0.05, 4.06)	0.01 (0.00, 0.56)	0.00 (0.00, 0.00)	0.00 (0.00, 0.00)	41 (18, 70)	10 (0, 31)	0 (0, 5)	0 (0, 0)	0 (0, 0)

ISO	Total number of stillbirths	Method	Fraction of stillbirths attributable to $PM_{2.5} > C_0$ μg/m^3					Number of stillbirths attributable to $PM_{2.5} > C_0$ μg/m^3				
			$C_0 = 5$	$C_0 = 10$	$C_0 = 15$	$C_0 = 25$	$C_0 = 35$	$C_0 = 5$	$C_0 = 10$	$C_0 = 15$	$C_0 = 25$	$C_0 = 35$
COM	692 (409, 991)	Xie et al. 2021	7.72 (0.93, 15.87)	1.82 (0.03, 9.97)	0.13 (0.00, 4.37)	0.04 (0.00, 0.80)	0.03 (0.00, 0.42)	53 (6, 128)	13 (0, 72)	1 (0, 32)	0 (0, 6)	0 (0, 3)
CPV	138 (83, 192)	Age-specific curves	31.34 (16.93, 44.94)	23.84 (12.68, 34.97)	15.50 (8.17, 23.16)	1.61 (0.88, 2.38)	0.00 (0.00, 0.01)	43 (19, 70)	33 (15, 54)	21 (10, 36)	2 (1, 4)	0 (0, 0)
CPV	138 (83, 192)	All-ages curve	30.42 (13.26, 44.60)	23.11 (4.97, 38.77)	15.27 (1.28, 32.28)	4.63 (0.08, 18.36)	1.19 (0.00, 8.20)	42 (14, 73)	32 (6, 63)	21 (2, 51)	6 (0, 27)	2 (0, 12)
CPV	138 (83, 192)	Zhang et al. 2021	16.49 (11.18, 21.08)	12.35 (6.79, 17.13)	7.99 (2.58, 12.99)	1.34 (0.08, 4.81)	0.08 (0.00, 0.84)	23 (12, 34)	17 (7, 27)	11 (3, 20)	2 (0, 7)	0 (0, 1)
CPV	138 (83, 192)	Xie et al. 2021	22.60 (8.58, 35.62)	17.01 (2.93, 30.96)	11.20 (0.81, 25.88)	3.35 (0.11, 15.05)	0.78 (0.05, 6.85)	31 (10, 55)	23 (3, 47)	15 (1, 38)	5 (0, 21)	1 (0, 10)
CRI	336 (317, 356)	Age-specific curves	25.76 (13.30, 37.88)	17.82 (9.04, 26.87)	9.06 (4.55, 13.97)	0.00 (0.00, 0.00)	0.00 (0.00, 0.00)	87 (44, 127)	60 (30, 90)	30 (15, 47)	0 (0, 0)	0 (0, 0)
CRI	336 (317, 356)	All-ages curve	25.29 (9.77, 38.68)	17.26 (2.30, 32.35)	9.15 (0.41, 25.35)	0.97 (0.00, 11.39)	0.05 (0.00, 4.03)	85 (32, 130)	58 (8, 108)	31 (1, 85)	3 (0, 37)	0 (0, 13)
CRI	336 (317, 356)	Zhang et al. 2021	13.50 (8.90, 17.62)	9.22 (4.43, 13.59)	4.79 (0.85, 9.33)	0.13 (0.00, 1.81)	0.00 (0.00, 0.11)	45 (30, 59)	31 (15, 46)	16 (3, 31)	0 (0, 6)	0 (0, 0)
CRI	336 (317, 356)	Xie et al. 2021	18.58 (6.32, 30.27)	12.65 (1.20, 25.08)	6.91 (0.19, 19.50)	1.74 (0.01, 8.51)	0.77 (0.00, 3.08)	62 (21, 104)	43 (4, 86)	23 (1, 67)	6 (0, 29)	3 (0, 10)
CUB	765 (735, 798)	Age-specific curves	10.71 (5.32, 16.37)	2.52 (1.25, 3.93)	0.00 (0.00, 0.10)	0.00 (0.00, 0.12)	0.00 (0.00, 0.00)	82 (41, 125)	19 (10, 30)	0 (0, 1)	0 (0, 1)	0 (0, 0)
CUB	765 (735, 798)	All-ages curve	10.42 (1.81, 19.71)	3.13 (0.06, 11.41)	0.56 (0.00, 5.19)	0.00 (0.00, 0.87)	0.00 (0.00, 0.37)	80 (14, 150)	24 (0, 88)	4 (0, 39)	0 (0, 7)	0 (0, 3)
CUB	765 (735, 798)	Zhang et al. 2021	5.33 (2.64, 7.79)	1.39 (0.13, 3.38)	0.03 (0.00, 0.78)	0.00 (0.00, 0.01)	0.00 (0.00, 0.00)	41 (20, 60)	11 (1, 26)	0 (0, 6)	0 (0, 0)	0 (0, 0)

ISO	Total number of stillbirths	Method	Fraction of stillbirths attributable to $PM_{2.5} > C_0$ μg/m^3					Number of stillbirths attributable to $PM_{2.5} > C_0$ μg/m^3				
			$C_0 = 5$	$C_0 = 10$	$C_0 = 15$	$C_0 = 25$	$C_0 = 35$	$C_0 = 5$	$C_0 = 10$	$C_0 = 15$	$C_0 = 25$	$C_0 = 35$
CUB	765 (735, 798)	Xie et al. 2021	7.46 (1.05, 14.67)	2.36 (0.10, 8.57)	0.50 (0.00, 3.91)	0.13 (0.00, 0.71)	0.06 (0.00, 0.30)	57 (8, 112)	18 (1, 65)	4 (0, 30)	1 (0, 5)	0 (0, 2)
CYP	36 (31, 41)	Age-specific curves	23.32 (10.85, 36.10)	15.30 (7.04, 24.39)	6.39 (2.94, 10.45)	0.00 (0.00, 0.00)	0.00 (0.00, 0.00)	8 (4, 13)	6 (2, 9)	2 (1, 4)	0 (0, 0)	0 (0, 0)
CYP	36 (31, 41)	All-ages curve	23.86 (8.28, 36.44)	16.37 (1.67, 29.97)	8.62 (0.24, 22.72)	1.69 (0.00, 9.01)	0.86 (0.00, 3.14)	9 (3, 13)	6 (1, 11)	3 (0, 8)	1 (0, 3)	0 (0, 1)
CYP	36 (31, 41)	Zhang et al. 2021	12.33 (7.93, 16.18)	8.02 (3.36, 12.15)	3.53 (0.35, 7.85)	0.03 (0.00, 0.88)	0.00 (0.00, 0.03)	4 (3, 6)	3 (1, 4)	1 (0, 3)	0 (0, 0)	0 (0, 0)
CYP	36 (31, 41)	Xie et al. 2021	16.94 (5.51, 28.32)	10.87 (0.85, 23.15)	5.24 (0.11, 17.53)	1.20 (0.00, 6.94)	0.53 (0.00, 2.13)	6 (2, 10)	4 (0, 8)	2 (0, 6)	0 (0, 2)	0 (0, 1)
DJI	650 (536, 756)	Age-specific curves	51.86 (31.21, 66.79)	46.67 (27.96, 60.84)	40.92 (24.90, 53.99)	27.68 (17.45, 37.00)	12.45 (8.41, 16.41)	337 (203, 448)	303 (183, 406)	266 (162, 358)	180 (115, 246)	81 (53, 112)
DJI	650 (536, 756)	All-ages curve	50.07 (29.69, 66.21)	44.80 (22.12, 62.61)	38.92 (14.03, 58.55)	25.67 (2.89, 49.42)	14.63 (0.72, 39.12)	325 (173, 454)	291 (130, 430)	253 (87, 404)	167 (17, 335)	95 (4, 260)
DJI	650 (536, 756)	Zhang et al. 2021	30.84 (22.64, 37.72)	27.37 (18.82, 34.65)	23.74 (14.76, 31.44)	15.89 (6.17, 24.49)	7.59 (0.91, 16.83)	200 (136, 259)	178 (115, 236)	154 (90, 213)	103 (39, 165)	49 (6, 111)
DJI	650 (536, 756)	Xie et al. 2021	40.79 (19.96, 57.68)	36.45 (14.00, 54.51)	31.75 (8.04, 51.14)	21.25 (1.40, 43.66)	11.26 (0.28, 34.72)	265 (124, 392)	237 (88, 368)	206 (51, 341)	138 (9, 290)	73 (2, 231)
DMA	12 (7, 18)	Age-specific curves	16.60 (8.39, 25.63)	7.69 (3.81, 12.25)	0.01 (0.01, 0.04)	0.00 (0.00, 0.06)	0.00 (0.00, 0.00)	2 (1, 4)	1 (0, 2)	0 (0, 0)	0 (0, 0)	0 (0, 0)
DMA	12 (7, 18)	All-ages curve	17.00 (4.06, 27.60)	9.13 (0.20, 20.30)	3.87 (0.00, 12.06)	0.88 (0.00, 3.16)	0.45 (0.00, 1.39)	2 (0, 4)	1 (0, 3)	0 (0, 2)	0 (0, 0)	0 (0, 0)
DMA	12 (7, 18)	Zhang et al. 2021	8.62 (4.99, 11.55)	4.26 (0.63, 7.17)	0.66 (0.00, 2.77)	0.00 (0.00, 0.04)	0.00 (0.00, 0.00)	1 (0, 2)	1 (0, 1)	0 (0, 0)	0 (0, 0)	0 (0, 0)

ISO	Total number of stillbirths	Method	Fraction of stillbirths attributable to $PM_{2.5} > C_0$ μg/m^3					Number of stillbirths attributable to $PM_{2.5} > C_0$ μg/m^3				
			$C_0 = 5$	$C_0 = 10$	$C_0 = 15$	$C_0 = 25$	$C_0 = 35$	$C_0 = 5$	$C_0 = 10$	$C_0 = 15$	$C_0 = 25$	$C_0 = 35$
DMA	12 (7, 18)	Xie et al. 2021	11.84 (2.37, 20.79)	5.81 (0.09, 14.91)	2.02 (0.00, 9.10)	0.13 (0.00, 2.25)	0.00 (0.00, 1.00)	1 (0, 3)	1 (0, 2)	0 (0, 1)	0 (0, 0)	0 (0, 0)
DOM	2,441 (1,457, 3,532)	Age-specific curves	15.44 (7.53, 23.26)	6.63 (3.17, 10.24)	0.42 (0.22, 0.66)	0.00 (0.00, 0.04)	0.00 (0.00, 0.00)	377 (161, 683)	162 (68, 301)	10 (5, 19)	0 (0, 1)	0 (0, 0)
DOM	2,441 (1,457, 3,532)	All-ages curve	14.99 (3.89, 25.81)	6.56 (0.33, 18.15)	2.04 (0.02, 10.71)	0.35 (0.00, 2.56)	0.08 (0.00, 0.93)	366 (81, 729)	160 (8, 496)	50 (0, 277)	9 (0, 65)	2 (0, 26)
DOM	2,441 (1,457, 3,532)	Zhang et al. 2021	7.88 (4.59, 10.77)	3.53 (0.80, 6.39)	0.68 (0.01, 2.39)	0.00 (0.00, 0.06)	0.00 (0.00, 0.00)	192 (86, 306)	86 (16, 169)	16 (0, 61)	0 (0, 1)	0 (0, 0)
DOM	2,441 (1,457, 3,532)	Xie et al. 2021	10.96 (2.39, 19.83)	5.14 (0.22, 14.12)	1.51 (0.02, 8.52)	0.28 (0.00, 1.87)	0.13 (0.00, 0.58)	268 (51, 527)	125 (5, 362)	37 (1, 214)	7 (0, 49)	3 (0, 14)
DZA	9,898 (8,829, 10,992)	Age-specific curves	20.80 (10.66, 31.60)	12.14 (6.29, 18.73)	4.37 (2.38, 6.54)	1.05 (0.71, 1.46)	0.38 (0.27, 0.52)	2,059 (1,035, 3,168)	1,202 (604, 1,876)	433 (225, 650)	103 (68, 146)	38 (26, 51)
DZA	9,898 (8,829, 10,992)	All-ages curve	20.19 (6.74, 31.86)	12.07 (1.85, 24.87)	5.72 (0.71, 17.32)	1.61 (0.15, 6.63)	0.71 (0.03, 2.99)	1,999 (641, 3,310)	1,194 (175, 2,561)	566 (69, 1,756)	159 (15, 665)	71 (3, 289)
DZA	9,898 (8,829, 10,992)	Zhang et al. 2021	10.66 (6.73, 14.14)	6.25 (2.48, 9.90)	2.60 (0.82, 5.68)	0.64 (0.20, 1.48)	0.25 (0.04, 0.61)	1,055 (650, 1,423)	618 (242, 983)	257 (80, 547)	64 (20, 143)	25 (4, 59)
DZA	9,898 (8,829, 10,992)	Xie et al. 2021	14.59 (4.31, 24.78)	8.50 (1.08, 19.18)	4.23 (0.38, 13.46)	1.40 (0.09, 5.42)	0.62 (0.03, 2.63)	1,444 (421, 2,486)	841 (108, 1,922)	419 (38, 1,352)	138 (8, 543)	61 (3, 254)
ECU	3,182 (2,358, 3,947)	Age-specific curves	23.44 (12.24, 34.49)	15.22 (7.83, 22.92)	6.54 (3.35, 10.02)	0.07 (0.04, 0.12)	0.00 (0.00, 0.00)	746 (376, 1,224)	484 (240, 814)	208 (103, 355)	2 (1, 4)	0 (0, 0)
ECU	3,182 (2,358, 3,947)	All-ages curve	22.65 (8.64, 35.91)	14.05 (2.02, 29.39)	6.88 (0.24, 21.93)	1.42 (0.01, 9.35)	0.23 (0.00, 3.46)	721 (243, 1,271)	447 (59, 1,002)	219 (8, 749)	45 (0, 318)	7 (0, 117)
ECU	3,182 (2,358, 3,947)	Zhang et al. 2021	12.13 (7.83, 16.00)	7.74 (3.35, 11.83)	3.53 (0.58, 7.48)	0.15 (0.00, 1.34)	0.01 (0.00, 0.09)	386 (226, 531)	246 (100, 394)	112 (18, 242)	5 (0, 44)	0 (0, 3)

ISO	Total number of stillbirths	Method	Fraction of stillbirths attributable to $PM_{2.5} > C_0$ μg/m^3					Number of stillbirths attributable to $PM_{2.5} > C_0$ μg/m^3				
			$C_0 = 5$	$C_0 = 10$	$C_0 = 15$	$C_0 = 25$	$C_0 = 35$	$C_0 = 5$	$C_0 = 10$	$C_0 = 15$	$C_0 = 25$	$C_0 = 35$
ECU	3,182 (2,358, 3,947)	Xie et al. 2021	16.90 (5.50, 27.79)	11.01 (1.12, 22.43)	5.62 (0.24, 16.68)	0.68 (0.02, 6.51)	0.28 (0.00, 2.10)	538 (177, 917)	350 (38, 738)	179 (8, 545)	22 (1, 211)	9 (0, 70)
EGY	26,474 (15,925, 38,258)	Age-specific curves	51.96 (32.81, 66.66)	46.80 (29.25, 60.93)	41.09 (25.49, 54.17)	28.04 (18.21, 37.38)	14.25 (9.63, 18.78)	13,755 (7,397, 21,466)	12,390 (6,633, 19,451)	10,878 (5,784, 17,124)	7,423 (4,077, 11,657)	3,772 (2,142, 5,844)
EGY	26,474 (15,925, 38,258)	All-ages curve	50.79 (30.39, 66.35)	45.48 (23.07, 62.72)	39.57 (15.20, 58.68)	26.32 (4.18, 49.40)	14.38 (1.02, 39.08)	13,446 (6,666, 21,866)	12,040 (5,500, 20,684)	10,475 (3,602, 19,097)	6,969 (1,164, 15,048)	3,806 (279, 11,234)
EGY	26,474 (15,925, 38,258)	Zhang et al. 2021	31.93 (23.53, 38.89)	28.57 (19.74, 35.89)	25.03 (15.77, 32.73)	17.43 (7.69, 25.95)	9.73 (1.80, 18.56)	8,454 (4,888, 13,347)	7,563 (4,349, 12,167)	6,626 (3,547, 10,980)	4,615 (1,846, 8,359)	2,576 (445, 5,749)
EGY	26,474 (15,925, 38,258)	Xie et al. 2021	42.01 (20.91, 59.00)	37.73 (15.12, 56.01)	33.12 (9.30, 52.78)	22.89 (2.16, 45.53)	12.90 (0.49, 37.23)	11,121 (5,205, 18,638)	9,989 (3,847, 17,218)	8,767 (2,384, 16,005)	6,059 (585, 13,193)	3,414 (147, 10,807)
ERI	2,110 (1,237, 2,984)	Age-specific curves	43.79 (25.38, 58.95)	37.71 (21.70, 51.53)	30.96 (17.75, 42.75)	15.39 (9.35, 21.13)	4.47 (3.03, 5.77)	924 (410, 1,389)	796 (352, 1,197)	653 (290, 985)	325 (154, 500)	94 (47, 146)
ERI	2,110 (1,237, 2,984)	All-ages curve	42.54 (23.57, 58.14)	36.65 (15.29, 53.60)	30.11 (7.91, 48.58)	16.30 (1.79, 37.06)	7.27 (0.48, 25.07)	898 (386, 1,388)	773 (251, 1,296)	635 (135, 1,171)	344 (33, 874)	153 (9, 565)
ERI	2,110 (1,237, 2,984)	Zhang et al. 2021	24.93 (17.85, 30.89)	21.24 (13.78, 27.48)	17.36 (9.50, 23.89)	9.06 (2.69, 16.21)	3.35 (0.67, 8.29)	526 (271, 759)	448 (222, 671)	366 (159, 578)	191 (48, 374)	71 (13, 182)
ERI	2,110 (1,237, 2,984)	Xie et al. 2021	33.33 (15.23, 48.90)	28.45 (9.03, 45.16)	23.19 (4.19, 41.13)	12.69 (0.91, 32.06)	6.38 (0.24, 22.23)	703 (260, 1,198)	600 (160, 1,087)	489 (83, 972)	268 (18, 719)	135 (5, 489)
ETH	94,731 (54,842, 134,031)	Age-specific curves	33.11 (18.22, 46.95)	25.82 (14.17, 37.39)	17.74 (9.68, 26.14)	3.32 (1.91, 4.76)	0.39 (0.26, 0.53)	31,364 (14,604, 49,939)	24,461 (11,333, 39,435)	16,805 (7,789, 27,370)	3,143 (1,556, 5,081)	369 (196, 595)
ETH	94,731 (54,842, 134,031)	All-ages curve	31.91 (15.03, 46.43)	24.50 (6.97, 40.80)	16.50 (2.35, 34.59)	5.45 (0.29, 21.27)	1.61 (0.05, 10.76)	30,231 (9,564, 54,188)	23,213 (4,117, 46,162)	15,634 (1,561, 38,507)	5,166 (237, 22,320)	1,528 (45, 10,395)
ETH	94,731 (54,842, 134,031)	Zhang et al. 2021	17.69 (12.12, 22.48)	13.64 (7.81, 18.64)	9.41 (3.67, 14.61)	2.50 (0.32, 6.48)	0.42 (0.02, 1.75)	16,756 (8,812, 24,901)	12,920 (5,605, 19,998)	8,913 (2,708, 15,090)	2,370 (258, 6,190)	399 (22, 1,642)

ISO	Total number of stillbirths	Method	Fraction of stillbirths attributable to $PM_{2.5} > C_0$ μg/m^3					Number of stillbirths attributable to $PM_{2.5} > C_0$ μg/m^3				
			$C_0 = 5$	$C_0 = 10$	$C_0 = 15$	$C_0 = 25$	$C_0 = 35$	$C_0 = 5$	$C_0 = 10$	$C_0 = 15$	$C_0 = 25$	$C_0 = 35$
ETH	94,731 (54,842, 134,031)	Xie et al. 2021	24.09 (9.62, 37.49)	18.59 (3.91, 32.95)	12.85 (1.11, 28.05)	4.18 (0.17, 17.57)	1.36 (0.06, 8.99)	22,819 (7,720, 38,957)	17,614 (3,593, 33,253)	12,169 (1,068, 27,026)	3,964 (149, 15,541)	1,286 (58, 7,203)
GAB	945 (542, 1,333)	Age-specific curves	38.03 (21.06, 52.87)	31.37 (17.28, 44.51)	23.99 (13.13, 34.58)	8.26 (4.66, 11.96)	0.24 (0.15, 0.34)	359 (148, 577)	296 (121, 482)	227 (94, 373)	78 (34, 131)	2 (1, 4)
GAB	945 (542, 1,333)	All-ages curve	36.74 (18.69, 52.39)	29.49 (9.97, 47.27)	21.60 (3.64, 41.72)	7.83 (0.34, 29.25)	2.02 (0.01, 17.24)	347 (117, 572)	279 (73, 501)	204 (27, 429)	74 (3, 298)	19 (0, 169)
GAB	945 (542, 1,333)	Zhang et al. 2021	20.92 (14.74, 26.45)	16.96 (10.57, 22.85)	12.80 (6.26, 19.06)	4.65 (0.58, 11.19)	0.58 (0.01, 4.03)	198 (94, 300)	160 (70, 254)	121 (43, 208)	44 (4, 113)	6 (0, 41)
GAB	945 (542, 1,333)	Xie et al. 2021	28.50 (11.99, 43.03)	23.42 (5.86, 38.91)	17.99 (2.09, 34.48)	7.73 (0.26, 24.70)	2.32 (0.07, 14.93)	269 (95, 440)	221 (47, 383)	170 (16, 327)	73 (2, 227)	22 (1, 135)
GEO	376 (356, 397)	Age-specific curves	30.19 (16.28, 43.22)	22.62 (12.01, 33.10)	14.22 (7.49, 21.17)	1.13 (0.61, 1.72)	0.00 (0.00, 0.00)	114 (60, 164)	85 (44, 126)	53 (27, 81)	4 (2, 7)	0 (0, 0)
GEO	376 (356, 397)	All-ages curve	29.76 (12.58, 43.64)	22.66 (4.45, 37.82)	14.96 (0.95, 31.37)	4.24 (0.07, 17.65)	1.39 (0.00, 7.51)	112 (46, 165)	85 (16, 142)	56 (4, 118)	16 (0, 66)	5 (0, 28)
GEO	376 (356, 397)	Zhang et al. 2021	16.00 (10.72, 20.47)	11.91 (6.27, 16.54)	7.61 (2.20, 12.45)	1.26 (0.03, 4.39)	0.01 (0.00, 0.62)	60 (40, 77)	45 (23, 62)	29 (8, 47)	5 (0, 17)	0 (0, 2)
GEO	376 (356, 397)	Xie et al. 2021	21.92 (8.24, 34.66)	16.34 (2.65, 29.91)	10.64 (0.67, 24.79)	3.19 (0.07, 14.09)	0.87 (0.03, 6.24)	82 (31, 130)	61 (10, 112)	40 (3, 93)	12 (0, 53)	3 (0, 24)
GHA	20,577 (16,942, 24,113)	Age-specific curves	41.89 (24.02, 57.08)	35.59 (20.22, 49.31)	28.60 (16.16, 40.12)	12.52 (7.49, 17.52)	3.04 (2.06, 4.05)	8,619 (4,832, 11,766)	7,323 (4,078, 10,168)	5,884 (3,257, 8,277)	2,576 (1,428, 3,612)	626 (403, 848)
GHA	20,577 (16,942, 24,113)	All-ages curve	40.47 (21.83, 56.21)	34.08 (13.43, 51.59)	26.98 (6.64, 46.45)	12.81 (1.31, 34.70)	5.11 (0.29, 22.64)	8,328 (4,136, 11,923)	7,013 (2,734, 10,947)	5,551 (1,403, 9,695)	2,636 (277, 6,790)	1,051 (62, 4,446)
GHA	20,577 (16,942, 24,113)	Zhang et al. 2021	23.44 (16.65, 29.20)	19.66 (12.52, 25.70)	15.68 (8.20, 22.02)	7.46 (1.88, 14.11)	2.31 (0.22, 6.62)	4,824 (3,132, 6,403)	4,045 (2,404, 5,608)	3,226 (1,576, 4,708)	1,535 (366, 2,940)	475 (44, 1,381)

ISO	Total number of stillbirths	Method	Fraction of stillbirths attributable to $PM_{2.5} > C_0$ μg/m^3					Number of stillbirths attributable to $PM_{2.5} > C_0$ μg/m^3				
			$C_0 = 5$	$C_0 = 10$	$C_0 = 15$	$C_0 = 25$	$C_0 = 35$	$C_0 = 5$	$C_0 = 10$	$C_0 = 15$	$C_0 = 25$	$C_0 = 35$
GHA	20,577 (16,942, 24,113)	Xie et al. 2021	31.41 (14.06, 46.69)	26.30 (7.85, 42.71)	20.79 (3.32, 38.42)	10.50 (0.56, 28.77)	4.63 (0.19, 18.89)	6,463 (2,947, 9,624)	5,412 (1,697, 8,714)	4,278 (721, 7,843)	2,160 (119, 5,897)	953 (41, 3,830)
GIN	11,359 (7,163, 16,376)	Age-specific curves	40.83 (23.13, 56.05)	34.44 (19.31, 48.26)	27.35 (15.16, 38.98)	11.07 (6.30, 16.04)	0.19 (0.13, 0.27)	4,638 (2,375, 7,464)	3,912 (1,987, 6,384)	3,107 (1,573, 5,122)	1,258 (643, 2,106)	22 (12, 36)
GIN	11,359 (7,163, 16,376)	All-ages curve	39.86 (20.49, 55.49)	33.55 (11.72, 50.78)	26.55 (4.64, 45.55)	12.02 (0.55, 33.79)	3.30 (0.04, 21.43)	4,528 (1,645, 7,012)	3,811 (963, 6,386)	3,016 (401, 5,694)	1,366 (50, 4,170)	375 (4, 2,543)
GIN	11,359 (7,163, 16,376)	Zhang et al. 2021	22.63 (16.00, 28.33)	18.80 (11.84, 24.80)	14.77 (7.47, 21.12)	6.15 (0.72, 13.22)	0.82 (0.01, 5.05)	2,571 (1,446, 3,802)	2,135 (1,099, 3,254)	1,678 (729, 2,741)	699 (80, 1,616)	93 (1, 613)
GIN	11,359 (7,163, 16,376)	Xie et al. 2021	30.45 (13.41, 45.67)	25.27 (7.14, 41.61)	19.67 (2.58, 37.21)	8.71 (0.27, 27.48)	3.07 (0.08, 16.79)	3,459 (1,384, 5,944)	2,871 (746, 5,272)	2,235 (269, 4,705)	990 (33, 3,421)	349 (9, 2,027)
GMB	1,972 (1,342, 2,649)	Age-specific curves	52.22 (31.69, 67.08)	47.06 (28.38, 61.18)	41.34 (25.31, 54.50)	28.18 (17.94, 37.76)	12.98 (8.63, 17.26)	1,030 (549, 1,571)	928 (489, 1,430)	815 (425, 1,266)	556 (296, 876)	256 (134, 403)
GMB	1,972 (1,342, 2,649)	All-ages curve	50.29 (29.88, 66.54)	44.89 (22.22, 62.93)	38.88 (13.93, 58.93)	25.13 (3.30, 49.74)	13.03 (0.69, 39.46)	992 (524, 1,522)	885 (387, 1,425)	767 (243, 1,319)	496 (60, 1,068)	257 (14, 799)
GMB	1,972 (1,342, 2,649)	Zhang et al. 2021	31.16 (22.80, 37.99)	27.78 (18.97, 34.92)	24.24 (14.96, 31.69)	16.62 (6.29, 24.73)	8.27 (0.81, 17.08)	614 (385, 898)	548 (328, 820)	478 (273, 735)	328 (125, 560)	163 (18, 369)
GMB	1,972 (1,342, 2,649)	Xie et al. 2021	41.16 (20.31, 58.01)	36.89 (14.57, 54.77)	32.27 (8.52, 51.26)	21.94 (1.61, 43.47)	11.71 (0.37, 34.44)	812 (378, 1,341)	727 (276, 1,259)	636 (159, 1,170)	433 (33, 996)	231 (7, 768)
GNB	2,287 (1,665, 2,974)	Age-specific curves	44.92 (26.01, 60.51)	38.97 (22.33, 53.40)	32.36 (18.38, 44.99)	17.15 (9.92, 24.24)	1.72 (1.08, 2.41)	1,027 (566, 1,497)	891 (489, 1,321)	740 (404, 1,113)	392 (211, 603)	39 (24, 61)
GNB	2,287 (1,665, 2,974)	All-ages curve	43.22 (24.23, 59.61)	36.80 (16.15, 55.38)	29.63 (8.05, 50.69)	14.12 (1.06, 39.97)	4.97 (0.12, 28.01)	988 (482, 1,577)	842 (299, 1,434)	678 (154, 1,314)	323 (25, 992)	114 (3, 664)
GNB	2,287 (1,665, 2,974)	Zhang et al. 2021	25.40 (18.21, 31.51)	21.70 (14.18, 28.13)	17.82 (9.99, 24.59)	9.47 (1.96, 16.95)	2.38 (0.07, 8.63)	581 (360, 825)	496 (276, 730)	408 (202, 614)	217 (42, 397)	54 (2, 199)

ISO	Total number of stillbirths	Method	Fraction of stillbirths attributable to $PM_{2.5} > C_0$ μg/m^3					Number of stillbirths attributable to $PM_{2.5} > C_0$ μg/m^3				
			$C_0 = 5$	$C_0 = 10$	$C_0 = 15$	$C_0 = 25$	$C_0 = 35$	$C_0 = 5$	$C_0 = 10$	$C_0 = 15$	$C_0 = 25$	$C_0 = 35$
GNB	2,287 (1,665, 2,974)	Xie et al. 2021	34.11 (15.67, 49.99)	29.36 (9.57, 46.35)	24.24 (4.09, 42.43)	13.24 (0.53, 33.63)	4.86 (0.10, 23.59)	780 (319, 1,294)	671 (203, 1,192)	554 (87, 1,073)	303 (11, 830)	111 (3, 575)
GNQ	627 (334, 909)	Age-specific curves	46.10 (26.87, 61.34)	40.42 (23.44, 54.47)	34.22 (19.83, 46.54)	20.22 (12.47, 27.88)	5.82 (3.93, 7.62)	289 (141, 465)	253 (123, 414)	215 (104, 354)	127 (64, 209)	36 (19, 58)
GNQ	627 (334, 909)	All-ages curve	45.33 (25.87, 61.25)	39.63 (17.82, 57.04)	33.39 (9.76, 52.36)	19.76 (1.90, 41.80)	9.37 (0.45, 30.41)	284 (120, 458)	248 (81, 421)	209 (47, 375)	124 (10, 288)	59 (3, 194)
GNQ	627 (334, 909)	Zhang et al. 2021	27.08 (19.59, 33.38)	23.47 (15.62, 30.08)	19.71 (11.51, 26.62)	11.73 (3.56, 19.30)	4.46 (0.66, 11.45)	170 (83, 252)	147 (71, 226)	124 (55, 200)	74 (20, 137)	28 (4, 82)
GNQ	627 (334, 909)	Xie et al. 2021	36.08 (16.99, 52.20)	31.42 (11.02, 48.74)	26.45 (5.68, 45.00)	15.83 (1.17, 36.72)	7.41 (0.33, 27.27)	226 (86, 364)	197 (56, 340)	166 (30, 315)	99 (7, 246)	46 (2, 180)
GRD	19 (11, 27)	Age-specific curves	19.68 (10.02, 30.01)	11.06 (5.54, 17.38)	1.54 (0.76, 2.48)	0.00 (0.00, 0.02)	0.00 (0.00, 0.00)	4 (2, 7)	2 (1, 4)	0 (0, 1)	0 (0, 0)	0 (0, 0)
GRD	19 (11, 27)	All-ages curve	19.45 (5.69, 31.71)	11.05 (0.56, 24.68)	3.45 (0.01, 16.80)	0.24 (0.00, 6.42)	0.16 (0.00, 2.33)	4 (1, 7)	2 (0, 5)	1 (0, 3)	0 (0, 1)	0 (0, 0)
GRD	19 (11, 27)	Zhang et al. 2021	10.12 (6.26, 13.56)	5.74 (1.47, 9.33)	1.31 (0.01, 4.96)	0.00 (0.00, 0.19)	0.00 (0.00, 0.00)	2 (1, 3)	1 (0, 2)	0 (0, 1)	0 (0, 0)	0 (0, 0)
GRD	19 (11, 27)	Xie et al. 2021	13.84 (3.19, 24.23)	7.46 (0.14, 18.74)	3.47 (0.01, 12.98)	1.05 (0.00, 3.90)	0.78 (0.00, 1.74)	3 (0, 5)	1 (0, 4)	1 (0, 3)	0 (0, 1)	0 (0, 0)
GTM	5,951 (5,091, 6,782)	Age-specific curves	37.93 (21.60, 52.08)	31.25 (17.76, 43.54)	23.85 (13.68, 33.48)	9.31 (5.77, 12.92)	2.45 (1.65, 3.23)	2,257 (1,278, 3,181)	1,860 (1,036, 2,666)	1,419 (780, 2,059)	554 (333, 788)	146 (93, 198)
GTM	5,951 (5,091, 6,782)	All-ages curve	36.99 (19.49, 52.16)	30.14 (11.44, 47.11)	22.68 (5.46, 41.52)	10.20 (1.10, 29.27)	4.07 (0.30, 18.42)	2,201 (1,077, 3,258)	1,793 (643, 2,935)	1,349 (318, 2,590)	607 (65, 1,777)	242 (18, 1,086)
GTM	5,951 (5,091, 6,782)	Zhang et al. 2021	21.27 (14.96, 26.65)	17.39 (10.76, 23.04)	13.32 (6.59, 19.24)	5.90 (1.57, 11.36)	1.97 (0.26, 5.20)	1,266 (834, 1,636)	1,035 (604, 1,416)	793 (367, 1,185)	351 (88, 689)	117 (14, 319)

ISO	Total number of stillbirths	Method	Fraction of stillbirths attributable to $PM_{2.5} > C_0$ μg/m^3					Number of stillbirths attributable to $PM_{2.5} > C_0$ μg/m^3				
			$C_0 = 5$	$C_0 = 10$	$C_0 = 15$	$C_0 = 25$	$C_0 = 35$	$C_0 = 5$	$C_0 = 10$	$C_0 = 15$	$C_0 = 25$	$C_0 = 35$
GTM	5,951 (5,091, 6,782)	Xie et al. 2021	28.65 (12.45, 43.09)	23.44 (6.59, 38.92)	17.91 (2.83, 34.42)	8.44 (0.57, 24.63)	3.65 (0.19, 15.64)	1,705 (697, 2,573)	1,395 (366, 2,309)	1,066 (161, 2,007)	502 (32, 1,367)	217 (11, 847)
GUY	236 (152, 315)	Age-specific curves	27.04 (14.22, 39.16)	19.16 (9.89, 28.48)	10.42 (5.29, 15.90)	0.00 (0.00, 0.00)	0.00 (0.00, 0.00)	64 (29, 101)	45 (20, 73)	25 (11, 40)	0 (0, 0)	0 (0, 0)
GUY	236 (152, 315)	All-ages curve	26.44 (10.24, 39.77)	18.56 (2.74, 33.30)	10.43 (0.24, 26.09)	3.04 (0.00, 12.59)	0.17 (0.00, 4.36)	62 (22, 108)	44 (6, 90)	25 (1, 72)	7 (0, 29)	0 (0, 10)
GUY	236 (152, 315)	Zhang et al. 2021	14.11 (9.34, 18.30)	9.86 (4.82, 14.29)	5.40 (0.79, 10.14)	0.23 (0.00, 2.01)	0.02 (0.00, 0.08)	33 (17, 48)	23 (9, 36)	13 (2, 24)	1 (0, 4)	0 (0, 0)
GUY	236 (152, 315)	Xie et al. 2021	19.65 (6.41, 31.51)	14.08 (1.28, 26.55)	8.20 (0.23, 20.92)	1.48 (0.00, 8.80)	0.26 (0.00, 2.97)	46 (15, 81)	33 (2, 66)	19 (0, 50)	4 (0, 22)	1 (0, 8)
HND	1,873 (1,776, 1,969)	Age-specific curves	39.22 (21.83, 54.03)	32.70 (18.08, 45.87)	25.48 (14.10, 36.26)	9.77 (5.53, 14.07)	0.56 (0.37, 0.79)	734 (417, 1,008)	612 (344, 856)	477 (265, 679)	183 (104, 266)	11 (7, 15)
HND	1,873 (1,776, 1,969)	All-ages curve	38.42 (19.91, 53.92)	31.80 (11.40, 49.00)	24.49 (4.68, 43.51)	10.70 (0.62, 31.15)	3.59 (0.10, 19.18)	720 (364, 1,023)	596 (209, 926)	459 (86, 820)	200 (11, 591)	67 (2, 360)
HND	1,873 (1,776, 1,969)	Zhang et al. 2021	21.87 (15.38, 27.40)	18.02 (11.20, 23.83)	13.99 (6.89, 20.07)	5.81 (0.82, 12.09)	0.96 (0.02, 4.70)	410 (286, 517)	338 (209, 450)	262 (130, 379)	109 (16, 231)	18 (0, 89)
HND	1,873 (1,776, 1,969)	Xie et al. 2021	29.50 (12.83, 44.41)	24.35 (6.66, 40.36)	18.83 (2.28, 35.99)	8.28 (0.30, 26.28)	2.88 (0.11, 16.30)	553 (239, 838)	456 (126, 758)	353 (43, 671)	155 (6, 486)	54 (2, 303)
HTI	5,802 (3,488, 8,126)	Age-specific curves	18.48 (9.70, 27.80)	9.53 (4.91, 14.69)	2.01 (1.06, 3.14)	0.00 (0.00, 0.04)	0.00 (0.00, 0.00)	1,072 (490, 1,844)	553 (250, 972)	117 (53, 207)	0 (0, 2)	0 (0, 0)
HTI	5,802 (3,488, 8,126)	All-ages curve	17.53 (5.18, 29.28)	8.63 (0.61, 21.92)	2.95 (0.09, 13.95)	0.33 (0.00, 3.96)	0.07 (0.00, 1.36)	1,017 (243, 1,934)	501 (30, 1,384)	171 (5, 857)	19 (0, 235)	4 (0, 68)
HTI	5,802 (3,488, 8,126)	Zhang et al. 2021	9.27 (5.65, 12.53)	4.79 (1.47, 8.22)	1.34 (0.11, 3.97)	0.01 (0.00, 0.22)	0.00 (0.00, 0.01)	538 (248, 825)	278 (71, 523)	78 (5, 238)	1 (0, 14)	0 (0, 0)

ISO	Total number of stillbirths	Method	Fraction of stillbirths attributable to $PM_{2.5} > C_0$ μg/m^3					Number of stillbirths attributable to $PM_{2.5} > C_0$ μg/m^3				
			$C_0 = 5$	$C_0 = 10$	$C_0 = 15$	$C_0 = 25$	$C_0 = 35$	$C_0 = 5$	$C_0 = 10$	$C_0 = 15$	$C_0 = 25$	$C_0 = 35$
HTI	5,802 (3,488, 8,126)	Xie et al. 2021	12.89 (3.43, 22.50)	6.79 (0.44, 16.86)	2.53 (0.06, 10.99)	0.24 (0.01, 3.10)	0.14 (0.00, 0.94)	748 (197, 1,378)	394 (28, 1,005)	147 (3, 613)	14 (0, 168)	8 (0, 52)
IDN	51,944 (41,914, 62,655)	Age-specific curves	33.17 (18.43, 46.23)	25.95 (14.38, 36.53)	18.53 (10.31, 26.05)	7.43 (4.61, 10.14)	2.32 (1.57, 3.01)	17,231 (9,687, 24,775)	13,482 (7,568, 19,486)	9,628 (5,461, 13,863)	3,857 (2,440, 5,449)	1,205 (757, 1,634)
IDN	51,944 (41,914, 62,655)	All-ages curve	32.29 (15.98, 46.27)	25.20 (8.88, 40.53)	18.22 (4.29, 34.30)	8.46 (0.91, 22.42)	3.61 (0.23, 13.92)	16,773 (7,489, 25,371)	13,091 (4,173, 21,862)	9,465 (2,070, 18,405)	4,392 (456, 11,850)	1,875 (111, 7,138)
IDN	51,944 (41,914, 62,655)	Zhang et al. 2021	18.37 (12.74, 23.22)	14.34 (8.56, 19.42)	10.42 (5.11, 15.52)	4.66 (1.45, 8.74)	1.87 (0.38, 4.32)	9,542 (6,098, 13,385)	7,450 (4,195, 11,110)	5,412 (2,541, 8,808)	2,419 (705, 4,870)	973 (191, 2,411)
IDN	51,944 (41,914, 62,655)	Xie et al. 2021	24.79 (10.23, 37.98)	19.38 (5.17, 33.42)	14.31 (2.45, 28.55)	6.95 (0.55, 19.36)	3.14 (0.18, 12.22)	12,878 (5,209, 20,331)	10,066 (2,698, 17,700)	7,435 (1,311, 14,896)	3,613 (302, 10,059)	1,629 (96, 6,393)
IND	421,034 (368,518, 470,823)	Age-specific curves	56.27 (40.10, 68.57)	51.50 (36.87, 63.49)	46.23 (32.90, 57.39)	34.67 (23.88, 43.41)	23.90 (14.63, 32.07)	236,922 (161,625, 302,679)	216,838 (150,653, 279,132)	194,625 (137,520, 250,658)	145,962 (99,074, 189,390)	100,634 (62,376, 140,146)
IND	421,034 (368,518, 470,823)	All-ages curve	55.37 (35.99, 68.22)	50.59 (29.53, 64.82)	45.31 (22.57, 61.04)	34.14 (11.68, 52.48)	24.07 (5.49, 43.44)	233,127 (156,665, 302,441)	212,987 (129,705, 287,483)	190,751 (100,932, 271,295)	143,723 (51,863, 230,341)	101,348 (24,337, 188,570)
IND	421,034 (368,518, 470,823)	Zhang et al. 2021	40.03 (30.65, 47.39)	37.07 (27.22, 44.79)	33.96 (23.69, 42.06)	27.54 (16.95, 36.27)	21.53 (11.39, 30.38)	168,538 (126,633, 206,809)	156,059 (111,988, 195,274)	142,986 (97,018, 183,199)	115,938 (70,792, 157,502)	90,653 (47,537, 131,593)
IND	421,034 (368,518, 470,823)	Xie et al. 2021	50.83 (28.08, 66.86)	47.24 (22.95, 64.43)	43.40 (18.08, 61.81)	35.39 (10.28, 56.01)	27.88 (5.23, 49.77)	214,023 (118,727, 288,799)	198,886 (97,616, 276,843)	182,734 (77,374, 263,955)	148,999 (44,400, 238,421)	117,404 (22,598, 211,861)
IRN	11,120 (6,436, 15,839)	Age-specific curves	47.93 (28.71, 62.42)	42.36 (25.30, 55.78)	36.17 (21.66, 48.09)	22.01 (14.02, 29.19)	8.98 (5.80, 11.52)	5,330 (2,324, 8,042)	4,710 (2,038, 7,197)	4,023 (1,814, 6,192)	2,448 (1,175, 3,695)	998 (516, 1,475)
IRN	11,120 (6,436, 15,839)	All-ages curve	46.79 (27.33, 62.61)	41.19 (19.46, 58.60)	34.95 (11.54, 54.13)	21.59 (3.23, 43.87)	11.34 (1.08, 32.76)	5,203 (2,341, 8,098)	4,580 (1,741, 7,432)	3,887 (1,061, 6,750)	2,400 (348, 5,249)	1,260 (112, 3,722)
IRN	11,120 (6,436, 15,839)	Zhang et al. 2021	28.92 (21.15, 35.38)	25.41 (17.25, 32.19)	21.72 (13.16, 28.84)	13.83 (5.62, 21.62)	7.01 (2.23, 14.01)	3,216 (1,587, 4,650)	2,826 (1,297, 4,174)	2,415 (989, 3,696)	1,538 (475, 2,714)	780 (196, 1,695)

ISO	Total number of stillbirths	Method	Fraction of stillbirths attributable to $PM_{2.5} > C_0$ μg/m^3					Number of stillbirths attributable to $PM_{2.5} > C_0$ μg/m^3				
			$C_0 = 5$	$C_0 = 10$	$C_0 = 15$	$C_0 = 25$	$C_0 = 35$	$C_0 = 5$	$C_0 = 10$	$C_0 = 15$	$C_0 = 25$	$C_0 = 35$
IRN	11,120 (6,436, 15,839)	Xie et al. 2021	38.20 (18.54, 54.32)	33.69 (12.60, 50.96)	28.83 (7.32, 47.33)	18.64 (2.45, 39.18)	10.70 (0.90, 30.09)	4,248 (1,437, 7,688)	3,747 (972, 7,190)	3,206 (538, 6,613)	2,073 (172, 5,367)	1,190 (66, 4,111)
IRQ	14,408 (8,531, 20,194)	Age-specific curves	56.91 (38.29, 70.55)	52.30 (35.43, 65.30)	47.20 (32.05, 59.47)	35.48 (25.03, 44.77)	22.11 (14.23, 28.17)	8,199 (4,688, 12,270)	7,536 (4,300, 11,386)	6,800 (3,932, 10,373)	5,112 (2,926, 7,710)	3,186 (1,716, 4,911)
IRQ	14,408 (8,531, 20,194)	All-ages curve	54.98 (33.51, 68.83)	50.13 (26.43, 65.49)	44.73 (18.74, 61.77)	32.34 (6.63, 53.22)	20.37 (2.14, 43.60)	7,922 (3,997, 11,617)	7,223 (3,120, 10,894)	6,444 (2,399, 10,047)	4,660 (1,049, 8,557)	2,935 (315, 6,790)
IRQ	14,408 (8,531, 20,194)	Zhang et al. 2021	36.59 (27.42, 44.00)	33.46 (23.83, 41.23)	30.17 (20.07, 38.33)	23.09 (12.04, 32.06)	15.42 (5.20, 25.15)	5,272 (3,086, 7,447)	4,821 (2,703, 6,858)	4,347 (2,289, 6,320)	3,326 (1,404, 5,077)	2,221 (625, 3,974)
IRQ	14,408 (8,531, 20,194)	Xie et al. 2021	47.56 (24.78, 64.77)	43.76 (19.24, 62.20)	39.66 (13.43, 59.43)	30.50 (4.88, 53.22)	20.83 (1.59, 45.98)	6,852 (2,540, 10,904)	6,304 (2,118, 10,363)	5,714 (1,631, 9,830)	4,394 (584, 8,595)	3,001 (198, 7,328)
ISR	467 (442, 490)	Age-specific curves	30.21 (15.69, 44.64)	22.71 (11.72, 34.41)	14.36 (7.41, 22.25)	0.42 (0.23, 0.65)	0.00 (0.00, 0.00)	141 (73, 210)	106 (54, 162)	67 (35, 105)	2 (1, 3)	0 (0, 0)
ISR	467 (442, 490)	All-ages curve	29.24 (13.03, 43.66)	21.43 (4.55, 38.03)	12.98 (0.96, 31.93)	3.14 (0.04, 17.63)	0.82 (0.00, 7.61)	137 (60, 207)	100 (21, 179)	61 (4, 149)	15 (0, 81)	4 (0, 35)
ISR	467 (442, 490)	Zhang et al. 2021	15.98 (10.81, 20.45)	11.86 (6.40, 16.54)	7.54 (2.11, 12.45)	0.91 (0.02, 3.97)	0.03 (0.00, 0.39)	75 (50, 96)	55 (30, 78)	35 (10, 59)	4 (0, 19)	0 (0, 2)
ISR	467 (442, 490)	Xie et al. 2021	21.85 (8.28, 34.70)	16.19 (2.63, 29.95)	10.23 (0.50, 24.84)	1.97 (0.04, 13.40)	0.58 (0.01, 5.33)	102 (38, 163)	76 (12, 140)	48 (2, 114)	9 (0, 62)	3 (0, 25)
JAM	673 (531, 815)	Age-specific curves	27.81 (14.73, 40.21)	19.99 (10.47, 29.51)	11.39 (6.03, 17.01)	1.40 (0.79, 2.09)	0.00 (0.00, 0.00)	187 (104, 265)	135 (74, 194)	77 (42, 111)	9 (5, 14)	0 (0, 0)
JAM	673 (531, 815)	All-ages curve	27.15 (11.27, 40.55)	19.49 (3.85, 34.34)	12.17 (0.84, 27.46)	3.17 (0.04, 15.49)	0.58 (0.00, 7.04)	183 (72, 291)	131 (25, 240)	82 (6, 193)	21 (0, 109)	4 (0, 49)
JAM	673 (531, 815)	Zhang et al. 2021	14.68 (9.71, 18.92)	10.51 (5.28, 14.93)	6.30 (1.94, 10.75)	1.35 (0.03, 3.90)	0.01 (0.00, 0.67)	99 (59, 137)	71 (32, 107)	42 (12, 75)	9 (0, 26)	0 (0, 5)

ISO	Total number of stillbirths	Method	Fraction of stillbirths attributable to $PM_{2.5} > C_0$ μg/m^3					Number of stillbirths attributable to $PM_{2.5} > C_0$ μg/m^3				
			$C_0 = 5$	$C_0 = 10$	$C_0 = 15$	$C_0 = 25$	$C_0 = 35$	$C_0 = 5$	$C_0 = 10$	$C_0 = 15$	$C_0 = 25$	$C_0 = 35$
JAM	673 (531, 815)	Xie et al. 2021	20.16 (7.24, 32.20)	14.52 (2.34, 27.19)	9.08 (0.52, 21.81)	2.40 (0.06, 11.64)	0.36 (0.02, 5.34)	136 (46, 232)	98 (15, 195)	61 (4, 154)	16 (0, 81)	2 (0, 38)
JOR	2,066 (1,431, 2,679)	Age-specific curves	43.17 (24.58, 58.14)	37.09 (20.90, 50.71)	30.34 (16.95, 41.93)	15.15 (8.96, 20.83)	4.77 (3.27, 6.33)	892 (483, 1,343)	766 (416, 1,169)	627 (343, 965)	313 (174, 479)	98 (59, 145)
JOR	2,066 (1,431, 2,679)	All-ages curve	42.07 (23.42, 57.83)	35.95 (15.26, 53.29)	29.14 (8.08, 48.22)	15.45 (1.56, 36.67)	6.67 (0.30, 24.96)	869 (402, 1,288)	743 (258, 1,174)	602 (161, 1,068)	319 (38, 792)	138 (8, 534)
JOR	2,066 (1,431, 2,679)	Zhang et al. 2021	24.71 (17.68, 30.65)	21.00 (13.59, 27.24)	17.11 (9.33, 23.65)	8.99 (2.73, 15.98)	3.42 (0.48, 8.63)	511 (320, 687)	434 (253, 598)	353 (178, 510)	186 (57, 331)	71 (10, 171)
JOR	2,066 (1,431, 2,679)	Xie et al. 2021	33.05 (15.10, 48.56)	28.15 (9.01, 44.81)	22.87 (4.28, 40.77)	12.65 (0.79, 31.73)	6.09 (0.19, 22.07)	683 (308, 1,065)	582 (176, 961)	472 (91, 853)	261 (17, 628)	126 (4, 419)
JPN	1,826 (1,766, 1,889)	Age-specific curves	15.16 (6.90, 24.07)	6.22 (2.79, 10.12)	0.52 (0.25, 0.87)	0.00 (0.00, 0.04)	0.00 (0.00, 0.00)	277 (123, 442)	113 (50, 186)	10 (5, 16)	0 (0, 1)	0 (0, 0)
JPN	1,826 (1,766, 1,889)	All-ages curve	15.28 (3.61, 25.48)	7.25 (0.34, 17.71)	2.46 (0.03, 10.23)	0.74 (0.00, 2.44)	0.32 (0.00, 0.90)	279 (66, 476)	132 (6, 331)	45 (0, 189)	14 (0, 45)	6 (0, 16)
JPN	1,826 (1,766, 1,889)	Zhang et al. 2021	7.72 (4.45, 10.63)	3.29 (0.74, 6.27)	0.53 (0.02, 2.32)	0.00 (0.00, 0.06)	0.00 (0.00, 0.00)	141 (81, 195)	60 (13, 115)	10 (0, 42)	0 (0, 1)	0 (0, 0)
JPN	1,826 (1,766, 1,889)	Xie et al. 2021	10.69 (2.16, 19.46)	4.91 (0.18, 13.59)	1.79 (0.03, 7.98)	0.33 (0.00, 1.89)	0.10 (0.00, 0.53)	195 (40, 362)	90 (3, 253)	33 (1, 149)	6 (0, 35)	2 (0, 10)
KAZ	2,362 (2,169, 2,562)	Age-specific curves	29.50 (15.91, 41.49)	21.81 (11.77, 30.99)	15.02 (8.20, 21.45)	5.15 (2.93, 7.46)	0.55 (0.36, 0.76)	697 (365, 994)	515 (270, 744)	355 (188, 516)	122 (69, 177)	13 (8, 18)
KAZ	2,362 (2,169, 2,562)	All-ages curve	28.64 (13.04, 42.01)	21.31 (6.44, 35.93)	15.14 (2.66, 29.50)	6.51 (0.38, 18.87)	2.30 (0.06, 11.02)	676 (301, 1,008)	503 (151, 857)	358 (63, 704)	154 (9, 452)	54 (1, 262)
KAZ	2,362 (2,169, 2,562)	Zhang et al. 2021	15.93 (10.78, 20.33)	11.83 (6.72, 16.38)	8.23 (3.77, 12.49)	3.10 (0.52, 6.81)	0.62 (0.04, 2.58)	376 (254, 481)	279 (160, 384)	194 (90, 291)	73 (13, 158)	15 (1, 61)

ISO	Total number of stillbirths	Method	Fraction of stillbirths attributable to $PM_{2.5} > C_0$ μg/m^3					Number of stillbirths attributable to $PM_{2.5} > C_0$ μg/m^3				
			C_0 = 5	C_0 = 10	C_0 = 15	C_0 = 25	C_0 = 35	C_0 = 5	C_0 = 10	C_0 = 15	C_0 = 25	C_0 = 35
KAZ	2,362 (2,169, 2,562)	Xie et al. 2021	21.61 (8.35, 34.00)	16.04 (3.77, 29.21)	11.22 (1.29, 24.19)	4.70 (0.20, 15.71)	1.81 (0.06, 8.98)	510 (191, 816)	379 (85, 697)	265 (30, 579)	111 (5, 380)	43 (1, 220)
KEN	29,221 (25,724, 32,519)	Age-specific curves	27.37 (14.50, 39.78)	19.56 (10.26, 29.02)	11.20 (5.90, 16.85)	0.93 (0.52, 1.39)	0.00 (0.00, 0.00)	7,998 (4,148, 11,718)	5,714 (2,928, 8,547)	3,274 (1,675, 4,974)	271 (155, 415)	0 (0, 0)
KEN	29,221 (25,724, 32,519)	All-ages curve	27.01 (10.92, 40.37)	19.50 (3.61, 34.12)	12.05 (0.84, 27.21)	3.32 (0.07, 14.03)	1.00 (0.00, 5.99)	7,893 (3,080, 12,535)	5,697 (1,018, 10,590)	3,520 (237, 8,282)	969 (19, 4,140)	293 (0, 1,786)
KEN	29,221 (25,724, 32,519)	Zhang et al. 2021	14.41 (9.61, 18.66)	10.16 (5.22, 14.64)	5.88 (1.76, 10.44)	0.76 (0.03, 3.23)	0.01 (0.00, 0.47)	4,212 (2,676, 5,591)	2,968 (1,475, 4,395)	1,720 (497, 3,155)	221 (10, 987)	3 (0, 140)
KEN	29,221 (25,724, 32,519)	Xie et al. 2021	19.77 (7.06, 31.91)	13.96 (2.11, 26.93)	8.53 (0.54, 21.58)	2.37 (0.08, 11.36)	0.60 (0.04, 4.75)	5,777 (2,075, 9,382)	4,079 (622, 7,851)	2,492 (159, 6,316)	693 (25, 3,327)	175 (12, 1,431)
KGZ	1,226 (1,130, 1,319)	Age-specific curves	31.76 (17.02, 45.03)	24.37 (12.86, 35.27)	16.24 (8.51, 23.94)	1.83 (1.00, 2.75)	0.01 (0.01, 0.02)	389 (203, 557)	299 (154, 436)	199 (102, 296)	22 (12, 34)	0 (0, 0)
KGZ	1,226 (1,130, 1,319)	All-ages curve	31.22 (13.77, 45.39)	24.16 (5.46, 39.71)	16.69 (1.51, 33.41)	5.56 (0.12, 20.00)	2.36 (0.01, 9.59)	383 (164, 573)	296 (66, 506)	205 (18, 424)	68 (1, 247)	29 (0, 118)
KGZ	1,226 (1,130, 1,319)	Zhang et al. 2021	16.91 (11.50, 21.61)	12.80 (7.12, 17.73)	8.53 (3.00, 13.70)	1.52 (0.07, 5.72)	0.06 (0.00, 0.93)	207 (140, 269)	157 (88, 221)	105 (38, 168)	19 (1, 69)	1 (0, 11)
KGZ	1,226 (1,130, 1,319)	Xie et al. 2021	23.12 (8.93, 36.25)	17.56 (3.15, 31.58)	11.82 (0.77, 26.53)	3.73 (0.10, 16.07)	1.27 (0.03, 7.79)	283 (111, 448)	215 (40, 391)	145 (10, 330)	46 (1, 201)	16 (0, 98)
KHM	5,120 (2,849, 7,266)	Age-specific curves	22.62 (11.65, 33.60)	14.30 (7.22, 21.80)	5.09 (2.54, 7.93)	0.00 (0.00, 0.00)	0.00 (0.00, 0.00)	1,158 (475, 1,997)	732 (295, 1,283)	261 (103, 467)	0 (0, 0)	0 (0, 0)
KHM	5,120 (2,849, 7,266)	All-ages curve	22.23 (7.66, 34.76)	13.99 (1.01, 28.00)	6.84 (0.13, 20.53)	0.70 (0.00, 6.98)	0.01 (0.00, 1.84)	1,138 (276, 1,866)	716 (44, 1,456)	350 (6, 1,040)	36 (0, 348)	0 (0, 107)
KHM	5,120 (2,849, 7,266)	Zhang et al. 2021	11.61 (7.48, 15.35)	7.19 (2.93, 11.19)	2.69 (0.23, 6.82)	0.05 (0.00, 0.61)	0.00 (0.00, 0.02)	595 (293, 943)	368 (124, 665)	138 (10, 376)	3 (0, 29)	0 (0, 1)

ISO	Total number of stillbirths	Method	Fraction of stillbirths attributable to $PM_{2.5} > C_0$ μg/m^3					Number of stillbirths attributable to $PM_{2.5} > C_0$ μg/m^3				
			$C_0 = 5$	$C_0 = 10$	$C_0 = 15$	$C_0 = 25$	$C_0 = 35$	$C_0 = 5$	$C_0 = 10$	$C_0 = 15$	$C_0 = 25$	$C_0 = 35$
KHM	5,120 (2,849, 7,266)	Xie et al. 2021	15.97 (4.75, 26.99)	9.70 (0.73, 21.52)	4.26 (0.12, 15.53)	0.39 (0.00, 5.62)	0.05 (0.00, 1.67)	817 (188, 1,546)	497 (28, 1,190)	218 (6, 828)	20 (0, 269)	3 (0, 83)
KNA	6 (3, 9)	Age-specific curves	14.59 (7.12, 23.02)	5.53 (2.65, 8.99)	0.00 (0.00, 0.08)	0.00 (0.00, 0.10)	0.00 (0.00, 0.00)	1 (0, 2)	0 (0, 1)	0 (0, 0)	0 (0, 0)	0 (0, 0)
KNA	6 (3, 9)	All-ages curve	14.06 (3.06, 24.89)	5.56 (0.05, 16.97)	2.30 (0.00, 9.25)	0.32 (0.00, 2.12)	0.02 (0.00, 0.98)	1 (0, 2)	0 (0, 1)	0 (0, 1)	0 (0, 0)	0 (0, 0)
KNA	6 (3, 9)	Zhang et al. 2021	7.33 (4.30, 10.31)	2.64 (0.37, 5.86)	0.23 (0.00, 1.65)	0.00 (0.00, 0.01)	0.00 (0.00, 0.00)	0 (0, 1)	0 (0, 0)	0 (0, 0)	0 (0, 0)	0 (0, 0)
KNA	6 (3, 9)	Xie et al. 2021	10.69 (1.92, 19.01)	4.74 (0.14, 13.23)	1.36 (0.00, 7.38)	0.16 (0.00, 1.38)	0.00 (0.00, 0.60)	1 (0, 1)	0 (0, 1)	0 (0, 0)	0 (0, 0)	0 (0, 0)
KOR	792 (760, 826)	Age-specific curves	32.52 (16.46, 46.57)	25.36 (12.68, 37.14)	17.43 (8.66, 26.03)	1.98 (1.03, 2.98)	0.00 (0.00, 0.00)	258 (132, 370)	201 (102, 295)	138 (69, 207)	16 (8, 24)	0 (0, 0)
KOR	792 (760, 826)	All-ages curve	32.00 (15.12, 46.72)	24.56 (6.32, 41.26)	16.39 (1.73, 35.23)	4.60 (0.12, 21.63)	1.05 (0.00, 10.25)	253 (117, 375)	195 (50, 329)	130 (14, 279)	36 (1, 168)	8 (0, 81)
KOR	792 (760, 826)	Zhang et al. 2021	17.64 (12.09, 22.44)	13.59 (7.78, 18.58)	9.34 (3.36, 14.57)	1.92 (0.07, 6.19)	0.14 (0.00, 1.01)	140 (95, 182)	108 (61, 151)	74 (26, 119)	15 (1, 50)	1 (0, 8)
KOR	792 (760, 826)	Xie et al. 2021	24.08 (9.56, 37.55)	18.61 (3.70, 33.01)	12.76 (0.84, 28.12)	3.57 (0.09, 17.23)	1.15 (0.03, 8.06)	191 (75, 296)	147 (29, 261)	101 (7, 223)	28 (1, 139)	9 (0, 65)
KWT	368 (210, 525)	Age-specific curves	66.08 (48.76, 77.87)	62.49 (47.27, 73.86)	58.50 (44.24, 69.42)	49.29 (34.91, 59.49)	38.49 (25.13, 49.25)	243 (114, 355)	230 (107, 337)	215 (101, 315)	181 (86, 267)	142 (60, 215)
KWT	368 (210, 525)	All-ages curve	61.34 (40.88, 74.36)	57.19 (34.43, 71.69)	52.56 (27.24, 68.73)	41.89 (12.32, 61.93)	29.87 (4.47, 54.22)	226 (106, 324)	210 (90, 309)	193 (74, 293)	154 (43, 253)	110 (16, 212)
KWT	368 (210, 525)	Zhang et al. 2021	44.38 (33.97, 52.50)	41.65 (30.72, 50.16)	38.78 (27.30, 47.69)	32.61 (19.96, 42.38)	25.80 (11.89, 36.51)	163 (79, 234)	153 (74, 221)	143 (68, 207)	120 (53, 182)	95 (35, 153)

ISO	Total number of stillbirths	Method	Fraction of stillbirths attributable to $PM_{2.5} > C_0$ μg/m^3					Number of stillbirths attributable to $PM_{2.5} > C_0$ μg/m^3				
			$C_0 = 5$	$C_0 = 10$	$C_0 = 15$	$C_0 = 25$	$C_0 = 35$	$C_0 = 5$	$C_0 = 10$	$C_0 = 15$	$C_0 = 25$	$C_0 = 35$
KWT	368 (210, 525)	Xie et al. 2021	56.58 (31.37, 73.99)	53.43 (26.35, 72.10)	50.04 (20.93, 70.05)	42.43 (9.89, 65.48)	33.61 (3.42, 60.14)	208 (75, 319)	197 (64, 310)	184 (51, 300)	156 (30, 278)	124 (10, 254)
LAO	3,124 (2,004, 4,202)	Age-specific curves	35.22 (19.48, 49.20)	28.21 (15.51, 40.16)	20.43 (11.28, 29.51)	5.69 (3.16, 8.33)	0.07 (0.05, 0.10)	1,100 (548, 1,733)	881 (436, 1,414)	638 (319, 1,039)	178 (95, 289)	2 (1, 4)
LAO	3,124 (2,004, 4,202)	All-ages curve	34.36 (16.57, 49.11)	27.32 (8.24, 43.63)	19.64 (2.94, 37.66)	7.61 (0.30, 24.66)	2.54 (0.01, 13.63)	1,073 (421, 1,787)	853 (204, 1,582)	613 (77, 1,334)	238 (9, 849)	79 (0, 437)
LAO	3,124 (2,004, 4,202)	Zhang et al. 2021	19.13 (13.27, 24.23)	15.12 (9.00, 20.51)	10.91 (4.78, 16.59)	3.41 (0.40, 8.42)	0.44 (0.00, 2.61)	598 (329, 866)	472 (238, 697)	341 (126, 527)	106 (11, 245)	14 (0, 78)
LAO	3,124 (2,004, 4,202)	Xie et al. 2021	26.01 (10.69, 39.91)	20.65 (4.86, 35.50)	15.00 (1.66, 30.73)	5.88 (0.18, 20.34)	1.72 (0.07, 11.22)	813 (278, 1,397)	645 (126, 1,242)	469 (41, 1,040)	184 (6, 692)	54 (2, 380)
LBN	768 (451, 1,089)	Age-specific curves	39.86 (22.53, 54.86)	33.49 (18.90, 46.91)	26.44 (14.92, 37.58)	10.67 (6.04, 15.40)	0.43 (0.27, 0.62)	306 (149, 500)	257 (124, 426)	203 (99, 341)	82 (41, 139)	3 (2, 6)
LBN	768 (451, 1,089)	All-ages curve	39.51 (20.55, 54.91)	33.16 (12.00, 50.01)	26.11 (4.99, 44.69)	12.34 (0.51, 32.34)	4.48 (0.04, 20.53)	303 (128, 496)	255 (79, 449)	201 (38, 394)	95 (4, 273)	34 (0, 175)
LBN	768 (451, 1,089)	Zhang et al. 2021	22.46 (15.86, 28.09)	18.60 (11.72, 24.49)	14.54 (7.40, 20.74)	6.16 (0.87, 12.76)	1.06 (0.01, 5.28)	172 (93, 259)	143 (73, 220)	112 (50, 184)	47 (7, 109)	8 (0, 40)
LBN	768 (451, 1,089)	Xie et al. 2021	30.25 (13.27, 45.36)	25.09 (7.08, 41.33)	19.54 (2.68, 36.97)	8.65 (0.33, 27.19)	2.65 (0.07, 17.09)	232 (93, 407)	193 (57, 367)	150 (24, 330)	66 (3, 241)	20 (1, 144)
LBR	3,900 (2,352, 5,505)	Age-specific curves	34.45 (18.70, 48.78)	27.37 (14.69, 39.65)	19.51 (10.42, 28.89)	2.54 (1.39, 3.81)	0.00 (0.00, 0.00)	1,343 (619, 2,252)	1,067 (491, 1,826)	761 (350, 1,323)	99 (46, 174)	0 (0, 0)
LBR	3,900 (2,352, 5,505)	All-ages curve	33.43 (15.87, 48.33)	26.19 (7.27, 42.83)	18.14 (2.04, 36.71)	4.78 (0.16, 22.84)	1.04 (0.00, 11.59)	1,304 (462, 2,303)	1,021 (229, 2,017)	707 (74, 1,707)	187 (6, 1,059)	40 (0, 505)
LBR	3,900 (2,352, 5,505)	Zhang et al. 2021	18.50 (12.71, 23.47)	14.48 (8.39, 19.68)	10.26 (3.89, 15.70)	2.31 (0.07, 7.19)	0.14 (0.00, 1.31)	722 (396, 1,083)	565 (261, 878)	400 (122, 664)	90 (2, 277)	6 (0, 47)

ISO	Total number of stillbirths	Method	Fraction of stillbirths attributable to $PM_{2.5} > C_0$ μg/m^3					Number of stillbirths attributable to $PM_{2.5} > C_0$ μg/m^3				
			$C_0 = 5$	$C_0 = 10$	$C_0 = 15$	$C_0 = 25$	$C_0 = 35$	$C_0 = 5$	$C_0 = 10$	$C_0 = 15$	$C_0 = 25$	$C_0 = 35$
LBR	3,900 (2,352, 5,505)	Xie et al. 2021	25.15 (10.25, 39.00)	19.66 (4.04, 34.52)	13.78 (0.98, 29.68)	4.49 (0.14, 18.90)	1.19 (0.03, 9.22)	981 (375, 1,737)	767 (162, 1,493)	537 (41, 1,247)	175 (6, 817)	47 (1, 397)
LBY	1,249 (736, 1,796)	Age-specific curves	27.86 (14.66, 40.57)	20.15 (10.50, 29.93)	11.61 (6.09, 17.33)	2.50 (1.50, 3.52)	0.55 (0.36, 0.75)	348 (162, 578)	252 (117, 421)	145 (69, 244)	31 (17, 50)	7 (4, 11)
LBY	1,249 (736, 1,796)	All-ages curve	27.58 (11.42, 40.96)	20.08 (4.07, 34.79)	12.82 (1.63, 27.96)	3.87 (0.26, 14.18)	1.20 (0.05, 6.17)	344 (115, 587)	251 (45, 486)	160 (17, 379)	48 (3, 191)	15 (1, 88)
LBY	1,249 (736, 1,796)	Zhang et al. 2021	14.89 (9.95, 19.20)	10.69 (5.47, 15.23)	6.32 (2.03, 11.05)	1.54 (0.33, 3.73)	0.46 (0.03, 1.36)	186 (101, 279)	134 (60, 214)	79 (22, 153)	19 (4, 48)	6 (0, 18)
LBY	1,249 (736, 1,796)	Xie et al. 2021	20.35 (7.50, 32.55)	14.57 (2.52, 27.61)	8.75 (0.86, 22.32)	2.78 (0.14, 11.81)	0.79 (0.05, 5.67)	254 (76, 430)	182 (27, 367)	109 (9, 297)	35 (2, 146)	10 (1, 67)
LCA	26 (23, 29)	Age-specific curves	18.65 (9.51, 28.17)	9.84 (4.93, 15.28)	0.46 (0.23, 0.73)	0.00 (0.00, 0.03)	0.00 (0.00, 0.00)	5 (2, 7)	3 (1, 4)	0 (0, 0)	0 (0, 0)	0 (0, 0)
LCA	26 (23, 29)	All-ages curve	17.77 (5.26, 30.15)	8.77 (0.25, 23.19)	3.27 (0.00, 15.20)	0.79 (0.00, 4.91)	0.11 (0.00, 1.68)	5 (1, 8)	2 (0, 6)	1 (0, 4)	0 (0, 1)	0 (0, 0)
LCA	26 (23, 29)	Zhang et al. 2021	9.49 (5.78, 12.79)	5.07 (1.11, 8.54)	0.98 (0.00, 4.18)	0.00 (0.00, 0.10)	0.00 (0.00, 0.00)	2 (1, 3)	1 (0, 2)	0 (0, 1)	0 (0, 0)	0 (0, 0)
LCA	26 (23, 29)	Xie et al. 2021	13.44 (3.31, 22.86)	7.59 (0.30, 17.39)	2.60 (0.00, 11.22)	0.76 (0.00, 3.16)	0.63 (0.00, 1.23)	3 (1, 6)	2 (0, 4)	1 (0, 3)	0 (0, 1)	0 (0, 0)
LKA	2,212 (2,096, 2,329)	Age-specific curves	33.45 (17.72, 48.24)	26.35 (13.81, 38.89)	18.48 (9.63, 27.76)	3.08 (1.65, 4.62)	0.00 (0.00, 0.00)	740 (384, 1,068)	583 (300, 860)	409 (211, 615)	68 (37, 103)	0 (0, 0)
LKA	2,212 (2,096, 2,329)	All-ages curve	32.94 (15.10, 47.43)	25.91 (6.49, 41.93)	18.19 (1.83, 35.88)	6.01 (0.17, 22.40)	1.94 (0.01, 11.13)	729 (330, 1,078)	573 (144, 950)	402 (40, 806)	133 (4, 499)	43 (0, 248)
LKA	2,212 (2,096, 2,329)	Zhang et al. 2021	18.06 (12.43, 22.96)	14.01 (8.13, 19.14)	9.75 (3.80, 15.17)	2.18 (0.15, 6.87)	0.13 (0.00, 1.46)	399 (272, 508)	310 (179, 424)	216 (83, 334)	48 (3, 153)	3 (0, 32)

ISO	Total number of stillbirths	Method	Fraction of stillbirths attributable to $PM_{2.5} > C_0$ μg/m^3					Number of stillbirths attributable to $PM_{2.5} > C_0$ μg/m^3				
			$C_0 = 5$	$C_0 = 10$	$C_0 = 15$	$C_0 = 25$	$C_0 = 35$	$C_0 = 5$	$C_0 = 10$	$C_0 = 15$	$C_0 = 25$	$C_0 = 35$
LKA	2,212 (2,096, 2,329)	Xie et al. 2021	24.53 (9.82, 38.17)	18.94 (3.84, 33.58)	12.98 (1.00, 28.61)	4.26 (0.14, 17.88)	1.37 (0.05, 8.94)	543 (218, 843)	419 (85, 743)	287 (23, 635)	94 (3, 400)	30 (1, 192)
LSO	1,701 (1,231, 2,132)	Age-specific curves	35.27 (19.17, 49.83)	28.35 (15.31, 40.90)	20.69 (11.14, 30.38)	4.53 (2.46, 6.71)	0.00 (0.00, 0.00)	600 (287, 925)	482 (228, 757)	352 (166, 561)	77 (38, 123)	0 (0, 0)
LSO	1,701 (1,231, 2,132)	All-ages curve	34.49 (16.94, 49.59)	27.31 (8.14, 44.34)	19.38 (2.63, 38.53)	6.34 (0.29, 25.44)	1.45 (0.01, 13.74)	587 (259, 915)	465 (117, 826)	330 (37, 716)	108 (4, 449)	25 (0, 237)
LSO	1,701 (1,231, 2,132)	Zhang et al. 2021	19.25 (13.32, 24.35)	15.28 (9.05, 20.61)	11.10 (4.65, 16.68)	3.17 (0.20, 8.33)	0.26 (0.00, 2.03)	327 (195, 470)	260 (135, 389)	189 (71, 306)	54 (3, 151)	4 (0, 36)
LSO	1,701 (1,231, 2,132)	Xie et al. 2021	26.09 (10.80, 40.22)	20.65 (4.73, 35.86)	14.82 (1.29, 31.15)	4.94 (0.15, 20.70)	1.47 (0.06, 11.08)	444 (165, 749)	351 (74, 671)	252 (20, 577)	84 (2, 379)	25 (1, 192)
MAR	10,999 (8,737, 13,350)	Age-specific curves	27.45 (14.81, 40.41)	19.61 (10.45, 29.61)	11.07 (5.82, 17.05)	0.31 (0.18, 0.49)	0.00 (0.00, 0.00)	3,019 (1,510, 4,555)	2,157 (1,066, 3,309)	1,218 (597, 1,892)	34 (19, 54)	0 (0, 0)
MAR	10,999 (8,737, 13,350)	All-ages curve	26.45 (11.02, 40.23)	18.35 (3.59, 33.99)	10.22 (0.76, 27.06)	1.99 (0.03, 13.64)	0.39 (0.00, 5.53)	2,910 (1,098, 4,979)	2,018 (358, 4,167)	1,124 (79, 3,298)	218 (3, 1,628)	42 (0, 657)
MAR	10,999 (8,737, 13,350)	Zhang et al. 2021	14.34 (9.53, 18.57)	10.10 (5.12, 14.53)	5.78 (1.54, 10.31)	0.64 (0.01, 2.88)	0.02 (0.00, 0.28)	1,577 (1,031, 2,116)	1,111 (562, 1,646)	636 (157, 1,148)	71 (1, 323)	3 (0, 31)
MAR	10,999 (8,737, 13,350)	Xie et al. 2021	19.69 (7.05, 31.77)	13.85 (2.04, 26.77)	8.14 (0.42, 21.39)	1.50 (0.07, 10.80)	0.44 (0.02, 4.29)	2,165 (714, 3,715)	1,523 (202, 3,088)	895 (43, 2,463)	165 (7, 1,234)	48 (2, 459)
MDG	13,999 (9,395, 18,294)	Age-specific curves	18.38 (9.72, 27.18)	10.11 (5.30, 15.23)	3.72 (1.96, 5.69)	0.00 (0.00, 0.05)	0.00 (0.00, 0.00)	2,574 (1,253, 4,038)	1,416 (684, 2,258)	521 (251, 837)	0 (0, 8)	0 (0, 0)
MDG	13,999 (9,395, 18,294)	All-ages curve	17.67 (5.81, 29.09)	9.71 (1.22, 21.76)	4.34 (0.22, 14.80)	0.52 (0.00, 5.42)	0.08 (0.00, 1.83)	2,473 (677, 4,616)	1,360 (131, 3,435)	607 (24, 2,240)	72 (0, 777)	11 (0, 281)
MDG	13,999 (9,395, 18,294)	Zhang et al. 2021	9.38 (5.76, 12.62)	5.22 (2.03, 8.40)	2.13 (0.32, 4.71)	0.15 (0.00, 0.70)	0.01 (0.00, 0.04)	1,313 (752, 2,038)	731 (265, 1,329)	298 (42, 723)	22 (0, 97)	1 (0, 7)

ISO	Total number of stillbirths	Method	Fraction of stillbirths attributable to $PM_{2.5} > C_0$ μg/m^3					Number of stillbirths attributable to $PM_{2.5} > C_0$ μg/m^3				
			$C_0 = 5$	$C_0 = 10$	$C_0 = 15$	$C_0 = 25$	$C_0 = 35$	$C_0 = 5$	$C_0 = 10$	$C_0 = 15$	$C_0 = 25$	$C_0 = 35$
MDG	13,999 (9,395, 18,294)	Xie et al. 2021	12.96 (3.68, 22.48)	7.27 (0.64, 16.85)	3.20 (0.08, 11.47)	0.46 (0.02, 4.09)	0.21 (0.00, 1.18)	1,814 (480, 3,565)	1,018 (90, 2,656)	448 (13, 1,758)	65 (3, 600)	29 (0, 188)
MDV	48 (40, 56)	Age-specific curves	25.98 (13.53, 37.69)	18.01 (9.28, 26.57)	11.17 (5.72, 16.73)	0.73 (0.41, 1.11)	0.00 (0.00, 0.00)	12 (7, 19)	9 (5, 13)	5 (3, 8)	0 (0, 1)	0 (0, 0)
MDV	48 (40, 56)	All-ages curve	24.63 (10.37, 38.30)	16.93 (3.68, 31.99)	11.12 (0.76, 25.37)	2.35 (0.00, 14.13)	0.35 (0.00, 6.88)	12 (5, 19)	8 (2, 15)	5 (0, 12)	1 (0, 7)	0 (0, 3)
MDV	48 (40, 56)	Zhang et al. 2021	13.51 (8.99, 17.80)	9.20 (4.88, 13.81)	5.63 (1.83, 9.88)	0.34 (0.00, 3.83)	0.00 (0.00, 0.60)	6 (4, 9)	4 (2, 7)	3 (1, 5)	0 (0, 2)	0 (0, 0)
MDV	48 (40, 56)	Xie et al. 2021	18.74 (6.52, 30.08)	13.11 (2.21, 25.01)	8.78 (0.24, 20.09)	2.29 (0.00, 11.36)	0.53 (0.00, 5.41)	9 (3, 15)	6 (1, 12)	4 (0, 10)	1 (0, 5)	0 (0, 3)
MEX	15,333 (12,827, 17,931)	Age-specific curves	24.51 (12.82, 35.68)	16.85 (8.75, 24.99)	9.73 (5.10, 14.66)	0.64 (0.38, 0.98)	0.00 (0.00, 0.00)	3,759 (2,038, 5,685)	2,583 (1,386, 3,970)	1,491 (799, 2,328)	98 (56, 156)	0 (0, 0)
MEX	15,333 (12,827, 17,931)	All-ages curve	23.91 (9.84, 36.61)	16.26 (3.44, 29.99)	9.54 (0.87, 23.39)	2.16 (0.08, 11.97)	0.47 (0.00, 5.15)	3,666 (1,410, 5,815)	2,494 (499, 4,705)	1,462 (125, 3,633)	330 (11, 1,803)	73 (0, 772)
MEX	15,333 (12,827, 17,931)	Zhang et al. 2021	12.92 (8.48, 16.86)	8.83 (4.51, 12.85)	5.19 (1.54, 9.04)	0.76 (0.02, 2.88)	0.04 (0.00, 0.42)	1,982 (1,248, 2,718)	1,354 (664, 2,041)	796 (228, 1,413)	116 (3, 452)	5 (0, 64)
MEX	15,333 (12,827, 17,931)	Xie et al. 2021	17.71 (6.22, 28.98)	12.09 (1.82, 23.87)	7.13 (0.39, 18.77)	1.75 (0.08, 9.77)	0.52 (0.03, 3.98)	2,716 (961, 4,506)	1,853 (291, 3,705)	1,093 (59, 2,892)	268 (11, 1,478)	80 (4, 615)
MLI	16,163 (14,357, 18,095)	Age-specific curves	49.31 (30.27, 64.08)	43.84 (26.69, 57.66)	37.77 (23.12, 50.21)	23.84 (15.67, 32.13)	8.87 (6.01, 11.54)	7,970 (4,908, 10,506)	7,085 (4,352, 9,494)	6,105 (3,714, 8,288)	3,853 (2,438, 5,234)	1,433 (949, 1,943)
MLI	16,163 (14,357, 18,095)	All-ages curve	47.71 (27.94, 64.10)	42.02 (20.21, 60.24)	35.65 (12.06, 55.93)	21.64 (2.63, 46.03)	11.00 (0.59, 34.94)	7,712 (4,149, 10,397)	6,791 (3,052, 9,685)	5,762 (1,844, 8,983)	3,498 (424, 7,264)	1,778 (98, 5,359)
MLI	16,163 (14,357, 18,095)	Zhang et al. 2021	29.11 (21.25, 35.70)	25.60 (17.39, 32.51)	21.91 (13.34, 29.17)	13.96 (5.10, 21.97)	6.35 (0.93, 14.12)	4,705 (3,330, 5,956)	4,137 (2,719, 5,409)	3,541 (2,099, 4,862)	2,256 (807, 3,688)	1,026 (144, 2,349)

ISO	Total number of stillbirths	Method	Fraction of stillbirths attributable to $PM_{2.5} > C_0$ μg/m^3					Number of stillbirths attributable to $PM_{2.5} > C_0$ μg/m^3				
			$C_0 = 5$	$C_0 = 10$	$C_0 = 15$	$C_0 = 25$	$C_0 = 35$	$C_0 = 5$	$C_0 = 10$	$C_0 = 15$	$C_0 = 25$	$C_0 = 35$
MLI	16,163 (14,357, 18,095)	Xie et al. 2021	38.63 (18.64, 55.14)	34.18 (12.70, 51.83)	29.37 (7.11, 48.25)	18.68 (1.59, 40.20)	9.54 (0.41, 30.94)	6,245 (2,850, 9,005)	5,524 (1,921, 8,483)	4,746 (1,061, 7,884)	3,020 (237, 6,492)	1,541 (64, 4,889)
MMR	14,461 (8,373, 20,212)	Age-specific curves	44.48 (25.72, 59.65)	38.48 (22.01, 52.39)	31.82 (18.10, 43.81)	16.87 (10.16, 23.48)	3.71 (2.53, 4.87)	6,432 (3,167, 9,840)	5,565 (2,708, 8,560)	4,601 (2,214, 7,126)	2,440 (1,187, 3,842)	536 (275, 821)
MMR	14,461 (8,373, 20,212)	All-ages curve	43.09 (24.11, 58.97)	37.03 (15.96, 54.52)	30.30 (8.33, 49.55)	16.30 (1.57, 38.22)	7.14 (0.31, 26.48)	6,231 (2,321, 10,057)	5,354 (1,649, 9,004)	4,381 (925, 7,964)	2,357 (181, 5,874)	1,032 (36, 3,924)
MMR	14,461 (8,373, 20,212)	Zhang et al. 2021	25.35 (18.22, 31.42)	21.63 (14.20, 28.04)	17.74 (10.01, 24.48)	9.55 (2.67, 16.89)	3.10 (0.45, 9.13)	3,665 (1,860, 5,154)	3,128 (1,485, 4,520)	2,565 (1,095, 3,841)	1,381 (311, 2,521)	448 (53, 1,268)
MMR	14,461 (8,373, 20,212)	Xie et al. 2021	33.96 (15.64, 49.66)	29.19 (9.60, 45.99)	24.06 (4.64, 42.02)	13.47 (0.90, 33.12)	6.06 (0.23, 23.33)	4,912 (1,927, 8,062)	4,221 (1,194, 7,212)	3,480 (585, 6,577)	1,948 (123, 4,991)	876 (32, 3,381)
MNG	445 (402, 487)	Age-specific curves	42.99 (28.44, 54.66)	36.80 (25.12, 46.51)	30.35 (21.12, 38.08)	21.27 (14.65, 26.36)	15.38 (9.10, 20.61)	191 (127, 245)	164 (111, 209)	135 (95, 170)	95 (64, 121)	68 (41, 95)
MNG	445 (402, 487)	All-ages curve	41.55 (24.05, 54.26)	35.21 (17.86, 49.35)	29.14 (13.12, 43.95)	20.43 (5.80, 33.94)	13.97 (2.07, 26.50)	185 (107, 244)	157 (81, 222)	130 (59, 198)	91 (27, 153)	62 (10, 121)
MNG	445 (402, 487)	Zhang et al. 2021	27.74 (20.47, 33.66)	24.17 (16.62, 30.39)	20.67 (13.44, 27.00)	15.50 (9.25, 21.20)	11.92 (5.51, 17.33)	123 (89, 155)	108 (72, 139)	92 (59, 123)	69 (41, 95)	53 (25, 77)
MNG	445 (402, 487)	Xie et al. 2021	35.98 (17.94, 50.27)	31.29 (13.16, 46.63)	26.78 (9.99, 42.74)	20.52 (4.76, 35.37)	15.77 (1.64, 29.93)	160 (77, 230)	139 (57, 212)	119 (42, 193)	91 (20, 160)	70 (7, 135)
MOZ	25,838 (20,272, 30,912)	Age-specific curves	24.78 (13.48, 35.92)	16.59 (8.91, 24.61)	8.12 (4.31, 12.24)	0.05 (0.03, 0.09)	0.00 (0.00, 0.00)	6,401 (3,063, 9,946)	4,287 (2,027, 6,805)	2,098 (989, 3,370)	14 (8, 25)	0 (0, 0)
MOZ	25,838 (20,272, 30,912)	All-ages curve	24.17 (9.07, 37.04)	16.27 (2.33, 30.42)	9.00 (0.40, 23.24)	2.32 (0.03, 10.24)	0.84 (0.00, 3.95)	6,244 (2,132, 10,240)	4,203 (553, 8,279)	2,326 (92, 6,090)	601 (7, 2,523)	216 (0, 957)
MOZ	25,838 (20,272, 30,912)	Zhang et al. 2021	12.81 (8.36, 16.73)	8.50 (3.98, 12.60)	4.35 (1.01, 8.34)	0.30 (0.00, 1.97)	0.01 (0.00, 0.15)	3,310 (2,021, 4,625)	2,196 (955, 3,426)	1,124 (244, 2,264)	78 (1, 504)	2 (0, 41)

ISO	Total number of stillbirths	Method	Fraction of stillbirths attributable to $PM_{2.5} > C_0$ μg/m^3					Number of stillbirths attributable to $PM_{2.5} > C_0$ μg/m^3				
			C_0 = 5	C_0 = 10	C_0 = 15	C_0 = 25	C_0 = 35	C_0 = 5	C_0 = 10	C_0 = 15	C_0 = 25	C_0 = 35
MOZ	25,838 (20,272, 30,912)	Xie et al. 2021	17.61 (5.81, 28.99)	11.66 (1.41, 23.77)	6.50 (0.26, 18.04)	1.56 (0.04, 8.33)	0.49 (0.02, 3.22)	4,549 (1,258, 7,626)	3,013 (287, 6,052)	1,680 (57, 4,535)	404 (11, 1,997)	127 (4, 771)
MRT	3,374 (1,925, 4,765)	Age-specific curves	58.89 (39.55, 72.40)	54.46 (36.60, 67.50)	49.55 (33.43, 62.07)	38.24 (26.90, 48.08)	25.12 (17.02, 31.99)	1,987 (1,009, 2,972)	1,838 (925, 2,768)	1,672 (845, 2,531)	1,290 (669, 1,962)	847 (421, 1,308)
MRT	3,374 (1,925, 4,765)	All-ages curve	56.26 (34.11, 70.04)	51.75 (26.94, 66.85)	46.73 (18.98, 63.30)	35.23 (6.01, 55.25)	22.91 (1.58, 46.28)	1,898 (945, 2,697)	1,746 (728, 2,512)	1,577 (571, 2,363)	1,189 (190, 2,044)	773 (50, 1,653)
MRT	3,374 (1,925, 4,765)	Zhang et al. 2021	37.19 (27.86, 44.75)	34.08 (24.30, 42.03)	30.82 (20.55, 39.18)	23.79 (12.49, 33.04)	16.03 (4.48, 26.26)	1,255 (673, 1,833)	1,150 (596, 1,703)	1,040 (475, 1,578)	803 (328, 1,309)	541 (133, 1,020)
MRT	3,374 (1,925, 4,765)	Xie et al. 2021	48.37 (25.24, 65.89)	44.61 (19.77, 63.38)	40.54 (13.87, 60.67)	31.41 (4.45, 54.57)	21.22 (1.13, 47.45)	1,632 (547, 2,558)	1,505 (442, 2,426)	1,368 (324, 2,302)	1,060 (145, 1,988)	716 (34, 1,671)
MUS	132 (124, 140)	Age-specific curves	18.03 (9.06, 27.46)	9.35 (4.61, 14.63)	1.13 (0.57, 1.81)	0.00 (0.00, 0.04)	0.00 (0.00, 0.00)	24 (12, 36)	12 (6, 19)	1 (1, 2)	0 (0, 0)	0 (0, 0)
MUS	132 (124, 140)	All-ages curve	17.55 (5.27, 29.08)	9.06 (0.62, 22.11)	2.13 (0.03, 14.24)	0.07 (0.00, 4.02)	0.00 (0.00, 1.29)	23 (7, 40)	12 (1, 30)	3 (0, 19)	0 (0, 5)	0 (0, 2)
MUS	132 (124, 140)	Zhang et al. 2021	9.37 (5.53, 12.41)	5.16 (1.25, 8.12)	1.59 (0.02, 3.80)	0.04 (0.00, 0.12)	0.00 (0.00, 0.00)	12 (7, 17)	7 (2, 11)	2 (0, 5)	0 (0, 0)	0 (0, 0)
MUS	132 (124, 140)	Xie et al. 2021	12.54 (3.34, 22.03)	6.20 (0.33, 16.15)	2.32 (0.01, 10.24)	0.42 (0.00, 2.64)	0.01 (0.00, 0.83)	17 (4, 30)	8 (0, 22)	3 (0, 14)	1 (0, 4)	0 (0, 1)
MWI	10,561 (10,081, 11,066)	Age-specific curves	29.20 (15.63, 42.06)	21.55 (11.37, 31.80)	13.06 (6.83, 19.73)	0.20 (0.11, 0.31)	0.00 (0.00, 0.00)	3,084 (1,617, 4,443)	2,276 (1,171, 3,362)	1,380 (700, 2,086)	21 (12, 32)	0 (0, 0)
MWI	10,561 (10,081, 11,066)	All-ages curve	28.56 (11.81, 42.54)	21.00 (3.62, 36.71)	12.77 (0.55, 30.26)	2.38 (0.02, 15.78)	0.35 (0.00, 5.64)	3,016 (1,219, 4,550)	2,218 (365, 3,896)	1,348 (59, 3,206)	252 (2, 1,653)	37 (0, 582)
MWI	10,561 (10,081, 11,066)	Zhang et al. 2021	15.33 (10.31, 19.75)	11.13 (5.86, 15.80)	6.71 (1.69, 11.68)	0.54 (0.01, 3.38)	0.00 (0.00, 0.25)	1,619 (1,090, 2,100)	1,175 (621, 1,676)	709 (178, 1,222)	57 (1, 359)	0 (0, 26)

ISO	Total number of stillbirths	Method	Fraction of stillbirths attributable to $PM_{2.5} > C_0$ μg/m^3					Number of stillbirths attributable to $PM_{2.5} > C_0$ μg/m^3				
			$C_0 = 5$	$C_0 = 10$	$C_0 = 15$	$C_0 = 25$	$C_0 = 35$	$C_0 = 5$	$C_0 = 10$	$C_0 = 15$	$C_0 = 25$	$C_0 = 35$
MWI	10,561 (10,081, 11,066)	Xie et al. 2021	20.88 (7.69, 33.49)	14.93 (2.03, 28.58)	8.84 (0.41, 23.34)	1.96 (0.03, 12.21)	0.59 (0.00, 4.68)	2,205 (791, 3,564)	1,577 (206, 3,045)	934 (43, 2,486)	207 (3, 1,308)	63 (0, 491)
MYS	2,369 (2,296, 2,446)	Age-specific curves	31.60 (16.59, 45.98)	24.34 (12.67, 36.14)	16.45 (8.55, 24.74)	3.37 (1.82, 5.08)	0.00 (0.00, 0.00)	749 (392, 1,095)	577 (299, 861)	390 (202, 590)	80 (43, 121)	0 (0, 0)
MYS	2,369 (2,296, 2,446)	All-ages curve	30.95 (14.27, 45.32)	23.42 (6.30, 39.56)	15.68 (1.75, 33.18)	4.97 (0.15, 20.15)	1.20 (0.00, 10.10)	733 (335, 1,074)	555 (148, 934)	372 (41, 782)	118 (3, 472)	28 (0, 237)
MYS	2,369 (2,296, 2,446)	Zhang et al. 2021	17.06 (11.67, 21.78)	12.93 (7.37, 17.91)	8.73 (3.45, 13.88)	2.08 (0.14, 6.20)	0.17 (0.00, 1.44)	404 (274, 519)	306 (173, 428)	207 (82, 331)	49 (3, 147)	4 (0, 34)
MYS	2,369 (2,296, 2,446)	Xie et al. 2021	23.13 (9.08, 36.42)	17.37 (3.43, 31.73)	11.51 (0.91, 26.67)	3.32 (0.14, 16.13)	0.91 (0.04, 7.69)	548 (212, 867)	411 (80, 757)	273 (22, 635)	79 (3, 384)	22 (1, 184)
NAM	1,083 (615, 1,526)	Age-specific curves	22.85 (11.89, 33.66)	14.54 (7.48, 21.84)	6.58 (3.42, 9.97)	0.34 (0.20, 0.53)	0.00 (0.00, 0.00)	247 (116, 415)	157 (73, 268)	71 (33, 123)	4 (2, 7)	0 (0, 0)
NAM	1,083 (615, 1,526)	All-ages curve	22.13 (8.25, 34.66)	13.81 (2.27, 27.69)	7.15 (0.46, 20.22)	1.31 (0.03, 8.59)	0.32 (0.00, 3.25)	240 (80, 445)	150 (24, 351)	77 (5, 254)	14 (0, 104)	4 (0, 39)
NAM	1,083 (615, 1,526)	Zhang et al. 2021	11.82 (7.61, 15.60)	7.45 (3.33, 11.45)	3.63 (0.83, 7.21)	0.42 (0.01, 1.65)	0.02 (0.00, 0.21)	128 (58, 194)	81 (27, 138)	39 (8, 81)	5 (0, 18)	0 (0, 2)
NAM	1,083 (615, 1,526)	Xie et al. 2021	16.27 (5.27, 27.14)	10.25 (1.22, 21.77)	5.01 (0.23, 16.12)	1.03 (0.05, 7.07)	0.40 (0.01, 2.57)	176 (46, 346)	111 (12, 274)	54 (3, 201)	11 (1, 78)	4 (0, 29)
NER	19,860 (14,872, 25,676)	Age-specific curves	65.68 (50.15, 76.77)	62.00 (47.25, 72.79)	57.92 (43.55, 68.32)	48.61 (35.06, 58.77)	38.03 (25.37, 49.41)	13,044 (8,803, 17,469)	12,313 (8,404, 16,617)	11,504 (7,760, 15,627)	9,654 (6,326, 13,559)	7,554 (4,603, 10,914)
NER	19,860 (14,872, 25,676)	All-ages curve	63.76 (44.36, 75.76)	59.88 (38.38, 73.17)	55.56 (31.73, 70.28)	45.65 (18.61, 63.65)	34.64 (9.13, 56.16)	12,663 (8,423, 17,680)	11,892 (7,487, 16,965)	11,035 (6,234, 16,072)	9,066 (3,541, 14,249)	6,880 (1,685, 12,421)
NER	19,860 (14,872, 25,676)	Zhang et al. 2021	49.76 (38.95, 57.95)	47.29 (35.94, 55.87)	44.68 (32.78, 53.69)	39.07 (25.97, 48.98)	32.88 (18.64, 43.79)	9,883 (6,867, 13,054)	9,391 (6,322, 12,512)	8,874 (5,764, 11,943)	7,759 (4,619, 10,749)	6,530 (3,426, 9,553)

ISO	Total number of stillbirths	Method	Fraction of stillbirths attributable to $PM_{2.5} > C_0$ μg/m^3					Number of stillbirths attributable to $PM_{2.5} > C_0$ μg/m^3				
			$C_0 = 5$	$C_0 = 10$	$C_0 = 15$	$C_0 = 25$	$C_0 = 35$	$C_0 = 5$	$C_0 = 10$	$C_0 = 15$	$C_0 = 25$	$C_0 = 35$
NER	19,860 (14,872, 25,676)	Xie et al. 2021	62.02 (36.37, 78.23)	59.25 (31.71, 76.62)	56.26 (26.69, 74.89)	49.55 (16.49, 71.01)	41.81 (8.94, 66.47)	12,316 (6,515, 17,789)	11,766 (5,615, 17,309)	11,173 (4,904, 16,794)	9,842 (3,040, 15,765)	8,303 (1,629, 14,555)
NGA	166,992 (99,540, 235,302)	Age-specific curves	59.88 (43.06, 72.06)	55.57 (40.85, 66.92)	50.78 (37.25, 61.26)	39.90 (29.30, 47.78)	28.00 (19.80, 35.26)	99,994 (54,743, 148,284)	92,790 (50,873, 137,375)	84,799 (47,634, 125,598)	66,631 (37,663, 96,570)	46,756 (25,889, 69,960)
NGA	166,992 (99,540, 235,302)	All-ages curve	58.11 (38.51, 70.82)	53.72 (31.99, 67.71)	48.85 (25.68, 64.25)	37.96 (15.69, 56.34)	27.45 (8.07, 47.56)	97,033 (54,340, 145,650)	89,701 (48,113, 137,165)	81,578 (38,758, 126,879)	63,385 (24,915, 106,519)	45,846 (14,162, 86,991)
NGA	166,992 (99,540, 235,302)	Zhang et al. 2021	44.00 (34.31, 51.41)	41.23 (31.06, 49.01)	38.33 (27.66, 46.48)	32.12 (20.70, 41.06)	25.75 (15.43, 35.19)	73,481 (42,751, 104,110)	68,858 (39,330, 98,763)	64,003 (36,155, 92,846)	53,640 (28,355, 79,592)	43,003 (21,490, 64,953)
NGA	166,992 (99,540, 235,302)	Xie et al. 2021	54.91 (31.73, 70.32)	51.61 (26.71, 68.15)	48.06 (21.72, 65.81)	40.33 (14.36, 60.57)	32.93 (9.31, 54.57)	91,690 (43,120, 139,801)	86,188 (39,290, 134,415)	80,263 (32,192, 129,730)	67,343 (21,665, 118,567)	54,985 (14,249, 105,416)
NIC	1,588 (1,179, 2,038)	Age-specific curves	27.27 (14.34, 39.40)	19.50 (10.09, 28.80)	10.91 (5.61, 16.37)	1.11 (0.63, 1.65)	0.00 (0.00, 0.00)	433 (220, 674)	310 (156, 492)	173 (86, 279)	18 (10, 28)	0 (0, 0)
NIC	1,588 (1,179, 2,038)	All-ages curve	26.87 (11.04, 40.41)	19.04 (3.55, 34.17)	11.53 (0.96, 27.26)	3.06 (0.03, 13.55)	0.94 (0.00, 5.53)	427 (170, 763)	302 (58, 629)	183 (16, 492)	49 (0, 226)	15 (0, 93)
NIC	1,588 (1,179, 2,038)	Zhang et al. 2021	14.45 (9.66, 18.73)	10.20 (5.24, 14.73)	5.80 (1.71, 10.52)	0.87 (0.03, 3.17)	0.03 (0.00, 0.48)	230 (140, 335)	162 (77, 260)	92 (25, 185)	14 (0, 52)	0 (0, 9)
NIC	1,588 (1,179, 2,038)	Xie et al. 2021	19.98 (7.11, 31.96)	14.31 (2.12, 26.98)	8.60 (0.55, 21.71)	2.39 (0.09, 11.22)	0.50 (0.03, 4.77)	317 (108, 561)	227 (30, 466)	137 (8, 366)	38 (1, 179)	8 (1, 80)
NPL	11,217 (8,709, 13,806)	Age-specific curves	52.79 (34.81, 66.42)	47.71 (31.91, 60.47)	42.07 (28.12, 53.95)	29.34 (20.48, 37.37)	17.61 (10.41, 23.63)	5,922 (3,586, 7,945)	5,351 (3,252, 7,269)	4,720 (2,916, 6,485)	3,291 (2,044, 4,527)	1,975 (1,119, 2,924)
NPL	11,217 (8,709, 13,806)	All-ages curve	52.36 (31.78, 66.01)	47.19 (24.58, 62.38)	41.44 (17.52, 58.34)	29.14 (7.36, 49.08)	18.54 (2.52, 39.10)	5,873 (3,390, 8,240)	5,294 (2,696, 7,695)	4,649 (2,066, 7,140)	3,269 (847, 5,917)	2,080 (294, 4,603)
NPL	11,217 (8,709, 13,806)	Zhang et al. 2021	35.14 (26.34, 42.26)	31.92 (22.71, 39.40)	28.54 (18.89, 36.40)	21.37 (11.58, 29.94)	14.78 (6.28, 23.17)	3,942 (2,613, 5,035)	3,580 (2,251, 4,634)	3,201 (1,934, 4,230)	2,397 (1,177, 3,387)	1,657 (644, 2,525)

ISO	Total number of stillbirths	Method	Fraction of stillbirths attributable to $PM_{2.5} > C_0$ μg/m^3					Number of stillbirths attributable to $PM_{2.5} > C_0$ μg/m^3				
			$C_0 = 5$	$C_0 = 10$	$C_0 = 15$	$C_0 = 25$	$C_0 = 35$	$C_0 = 5$	$C_0 = 10$	$C_0 = 15$	$C_0 = 25$	$C_0 = 35$
NPL	11,217 (8,709, 13,806)	Xie et al. 2021	45.54 (23.76, 62.10)	41.54 (18.26, 59.32)	37.23 (13.07, 56.33)	27.96 (5.67, 49.60)	19.74 (2.08, 42.16)	5,108 (2,445, 7,635)	4,659 (1,887, 7,226)	4,176 (1,368, 6,773)	3,136 (612, 5,915)	2,214 (228, 4,898)
OMN	476 (444, 507)	Age-specific curves	54.95 (34.85, 69.21)	50.20 (31.46, 63.78)	44.93 (28.08, 57.40)	32.80 (21.65, 42.78)	18.79 (12.51, 24.63)	262 (166, 333)	239 (152, 308)	214 (135, 278)	156 (103, 202)	89 (60, 115)
OMN	476 (444, 507)	All-ages curve	53.00 (31.84, 67.89)	47.98 (24.58, 64.43)	42.39 (16.60, 60.58)	29.65 (4.74, 51.75)	17.32 (1.21, 41.84)	252 (153, 324)	228 (119, 307)	202 (80, 289)	141 (23, 246)	82 (6, 199)
OMN	476 (444, 507)	Zhang et al. 2021	34.00 (25.20, 41.17)	30.75 (21.49, 38.27)	27.34 (17.61, 35.22)	19.99 (9.34, 28.65)	12.08 (2.65, 21.41)	162 (118, 197)	146 (101, 183)	130 (83, 169)	95 (45, 138)	58 (13, 103)
OMN	476 (444, 507)	Xie et al. 2021	44.49 (22.60, 61.74)	40.40 (16.94, 58.94)	35.99 (11.01, 55.91)	26.09 (2.97, 49.12)	15.91 (0.80, 41.21)	212 (105, 296)	192 (79, 282)	171 (51, 268)	124 (14, 234)	76 (4, 196)
PAK	197,845 (174,271, 222,807)	Age-specific curves	59.77 (43.82, 72.05)	55.45 (40.04, 67.30)	50.70 (36.70, 61.87)	40.01 (27.64, 50.00)	28.53 (17.92, 38.56)	118,251 (82,448, 151,782)	109,696 (76,666, 141,738)	100,300 (71,998, 129,452)	79,150 (54,824, 103,524)	56,436 (34,023, 78,325)
PAK	197,845 (174,271, 222,807)	All-ages curve	58.78 (38.69, 71.50)	54.35 (32.19, 68.47)	49.45 (25.19, 65.12)	38.49 (12.37, 57.52)	27.26 (5.10, 49.20)	116,284 (77,533, 147,195)	107,526 (64,343, 140,224)	97,841 (50,150, 132,804)	76,153 (23,960, 115,834)	53,936 (9,803, 97,312)
PAK	197,845 (174,271, 222,807)	Zhang et al. 2021	42.65 (32.68, 50.46)	39.82 (29.37, 48.01)	36.87 (25.92, 45.45)	30.57 (18.72, 39.99)	23.93 (11.82, 34.09)	84,384 (63,453, 104,477)	78,784 (57,252, 99,226)	72,938 (50,628, 93,733)	60,487 (36,534, 82,561)	47,335 (23,227, 69,971)
PAK	197,845 (174,271, 222,807)	Xie et al. 2021	54.26 (30.11, 71.01)	50.94 (25.08, 68.89)	47.38 (19.86, 66.61)	39.60 (10.42, 61.55)	31.23 (4.42, 55.78)	107,346 (57,315, 141,092)	100,778 (47,351, 136,086)	93,747 (37,610, 131,179)	78,346 (19,889, 120,401)	61,787 (8,525, 108,512)
PAN	669 (375, 957)	Age-specific curves	22.09 (11.42, 32.75)	13.71 (6.96, 20.85)	4.53 (2.28, 7.06)	0.00 (0.00, 0.01)	0.00 (0.00, 0.00)	148 (65, 250)	92 (40, 157)	30 (13, 52)	0 (0, 0)	0 (0, 0)
PAN	669 (375, 957)	All-ages curve	21.40 (7.56, 34.21)	12.77 (1.18, 27.47)	5.68 (0.07, 20.16)	0.96 (0.00, 7.22)	0.35 (0.00, 2.29)	143 (41, 277)	85 (7, 213)	38 (0, 147)	6 (0, 50)	2 (0, 17)
PAN	669 (375, 957)	Zhang et al. 2021	11.43 (7.26, 15.12)	7.13 (2.70, 10.84)	2.88 (0.15, 6.39)	0.01 (0.00, 0.44)	0.00 (0.00, 0.02)	76 (38, 116)	48 (15, 80)	19 (1, 49)	0 (0, 3)	0 (0, 0)

ISO	Total number of stillbirths	Method	Fraction of stillbirths attributable to $PM_{2.5} > C_0$ μg/m^3					Number of stillbirths attributable to $PM_{2.5} > C_0$ μg/m^3				
			C_0 = 5	C_0 = 10	C_0 = 15	C_0 = 25	C_0 = 35	C_0 = 5	C_0 = 10	C_0 = 15	C_0 = 25	C_0 = 35
PAN	669 (375, 957)	Xie et al. 2021	15.95 (4.80, 26.52)	10.11 (0.65, 21.24)	4.76 (0.08, 15.63)	0.38 (0.00, 5.27)	0.24 (0.00, 1.46)	107 (27, 189)	68 (4, 142)	32 (0, 98)	3 (0, 32)	2 (0, 9)
PER	4,508 (3,837, 5,159)	Age-specific curves	34.51 (19.14, 48.26)	27.50 (15.20, 39.04)	20.02 (11.05, 28.65)	7.14 (4.08, 10.15)	1.06 (0.69, 1.48)	1,556 (840, 2,107)	1,239 (667, 1,706)	902 (485, 1,262)	322 (178, 454)	48 (31, 65)
PER	4,508 (3,837, 5,159)	All-ages curve	33.50 (16.54, 47.99)	26.43 (8.97, 42.47)	19.12 (4.01, 36.45)	7.91 (0.61, 24.25)	2.82 (0.12, 14.56)	1,510 (707, 2,309)	1,192 (387, 2,054)	862 (174, 1,757)	357 (28, 1,163)	127 (5, 703)
PER	4,508 (3,837, 5,159)	Zhang et al. 2021	18.90 (13.10, 23.90)	14.93 (8.92, 20.17)	10.92 (5.15, 16.32)	4.30 (0.84, 9.01)	1.02 (0.03, 3.69)	852 (554, 1,108)	673 (387, 931)	492 (227, 745)	194 (39, 408)	46 (1, 174)
PER	4,508 (3,837, 5,159)	Xie et al. 2021	25.58 (10.60, 39.20)	20.25 (5.17, 34.76)	14.95 (2.00, 30.03)	6.51 (0.26, 20.43)	2.17 (0.10, 12.18)	1,153 (490, 1,746)	913 (241, 1,524)	674 (93, 1,304)	293 (12, 867)	98 (5, 526)
PHL	25,416 (20,367, 30,749)	Age-specific curves	29.94 (16.14, 42.92)	22.31 (11.92, 32.69)	14.07 (7.50, 20.99)	1.20 (0.66, 1.79)	0.00 (0.00, 0.00)	7,608 (4,295, 11,680)	5,670 (3,169, 8,869)	3,577 (1,996, 5,705)	304 (176, 488)	0 (0, 0)
PHL	25,416 (20,367, 30,749)	All-ages curve	28.92 (12.67, 42.92)	21.23 (4.95, 36.93)	13.42 (1.26, 30.31)	3.60 (0.07, 16.98)	0.83 (0.00, 7.62)	7,350 (3,127, 11,718)	5,395 (1,235, 9,881)	3,410 (312, 7,971)	916 (17, 4,430)	211 (0, 2,000)
PHL	25,416 (20,367, 30,749)	Zhang et al. 2021	15.78 (10.65, 20.25)	11.63 (6.29, 16.31)	7.43 (2.49, 12.19)	1.33 (0.04, 4.55)	0.08 (0.00, 0.67)	4,011 (2,579, 5,453)	2,955 (1,556, 4,277)	1,889 (601, 3,165)	337 (11, 1,152)	20 (0, 161)
PHL	25,416 (20,367, 30,749)	Xie et al. 2021	21.59 (8.17, 34.24)	15.94 (2.84, 29.43)	10.25 (0.67, 24.27)	2.55 (0.07, 13.86)	0.62 (0.03, 6.17)	5,488 (2,007, 8,842)	4,051 (769, 7,524)	2,606 (186, 6,184)	647 (18, 3,396)	158 (8, 1,445)
PNG	3,844 (2,329, 5,533)	Age-specific curves	19.62 (10.40, 29.43)	10.78 (5.63, 16.58)	2.54 (1.31, 3.97)	0.00 (0.00, 0.03)	0.00 (0.00, 0.00)	754 (380, 1,353)	415 (207, 756)	98 (49, 180)	0 (0, 1)	0 (0, 0)
PNG	3,844 (2,329, 5,533)	All-ages curve	19.29 (5.70, 30.62)	11.24 (0.77, 23.33)	4.74 (0.09, 15.56)	1.35 (0.00, 4.84)	0.66 (0.00, 1.55)	741 (170, 1,435)	432 (22, 1,069)	182 (3, 689)	52 (0, 205)	25 (0, 61)
PNG	3,844 (2,329, 5,533)	Zhang et al. 2021	9.88 (6.09, 13.22)	5.46 (1.77, 8.94)	1.65 (0.12, 4.61)	0.02 (0.00, 0.29)	0.00 (0.00, 0.01)	380 (190, 642)	210 (63, 419)	63 (5, 204)	1 (0, 12)	0 (0, 0)

ISO	Total number of stillbirths	Method	Fraction of stillbirths attributable to $PM_{2.5} > C_0$ μg/m^3					Number of stillbirths attributable to $PM_{2.5} > C_0$ μg/m^3				
			$C_0 = 5$	$C_0 = 10$	$C_0 = 15$	$C_0 = 25$	$C_0 = 35$	$C_0 = 5$	$C_0 = 10$	$C_0 = 15$	$C_0 = 25$	$C_0 = 35$
PNG	3,844 (2,329, 5,533)	Xie et al. 2021	13.61 (3.55, 23.58)	7.53 (0.47, 17.98)	3.22 (0.09, 12.12)	0.63 (0.00, 3.82)	0.17 (0.00, 1.04)	523 (142, 1,005)	290 (18, 741)	124 (3, 473)	24 (0, 148)	6 (0, 41)
PRK	3,285 (2,019, 4,605)	Age-specific curves	36.41 (18.15, 52.59)	30.07 (15.11, 44.23)	23.06 (11.68, 34.38)	9.12 (5.19, 13.44)	1.57 (1.02, 2.15)	1,196 (514, 1,988)	988 (426, 1,662)	757 (331, 1,280)	300 (142, 494)	51 (27, 82)
PRK	3,285 (2,019, 4,605)	All-ages curve	36.98 (18.91, 52.12)	30.22 (10.67, 47.01)	22.78 (4.87, 41.33)	10.35 (0.74, 28.80)	3.93 (0.12, 17.67)	1,215 (455, 2,006)	993 (292, 1,787)	748 (148, 1,503)	340 (22, 1,025)	129 (3, 635)
PRK	3,285 (2,019, 4,605)	Zhang et al. 2021	21.04 (14.78, 26.42)	17.13 (10.57, 22.79)	13.02 (6.34, 18.97)	5.44 (1.19, 11.01)	1.40 (0.08, 4.93)	691 (382, 1,041)	563 (286, 879)	428 (182, 722)	179 (35, 395)	46 (2, 156)
PRK	3,285 (2,019, 4,605)	Xie et al. 2021	28.38 (12.15, 42.86)	23.13 (6.18, 38.66)	17.53 (2.52, 34.13)	8.12 (0.41, 24.15)	3.16 (0.11, 15.13)	932 (371, 1,569)	760 (181, 1,406)	576 (69, 1,230)	267 (12, 837)	104 (4, 501)
PRY	1,652 (1,127, 2,097)	Age-specific curves	18.56 (9.34, 28.10)	9.82 (4.85, 15.28)	0.87 (0.44, 1.38)	0.00 (0.00, 0.03)	0.00 (0.00, 0.00)	307 (144, 502)	162 (75, 271)	14 (7, 24)	0 (0, 1)	0 (0, 0)
PRY	1,652 (1,127, 2,097)	All-ages curve	18.44 (5.19, 29.91)	10.04 (0.27, 22.81)	3.68 (0.02, 14.80)	0.10 (0.00, 3.61)	0.01 (0.00, 1.44)	305 (73, 539)	166 (5, 396)	61 (0, 244)	2 (0, 58)	0 (0, 23)
PRY	1,652 (1,127, 2,097)	Zhang et al. 2021	9.43 (5.83, 12.79)	4.91 (1.30, 8.42)	0.88 (0.02, 3.93)	0.00 (0.00, 0.12)	0.00 (0.00, 0.00)	156 (82, 225)	81 (20, 146)	15 (0, 64)	0 (0, 2)	0 (0, 0)
PRY	1,652 (1,127, 2,097)	Xie et al. 2021	13.01 (3.19, 22.77)	6.65 (0.34, 16.92)	2.07 (0.05, 11.03)	0.15 (0.00, 2.94)	0.03 (0.00, 1.08)	215 (46, 387)	110 (5, 275)	34 (1, 170)	3 (0, 44)	1 (0, 17)
PSE	1,598 (935, 2,272)	Age-specific curves	31.84 (16.85, 45.27)	24.48 (12.79, 35.67)	16.33 (8.48, 24.32)	0.96 (0.53, 1.44)	0.00 (0.00, 0.00)	509 (205, 811)	391 (156, 635)	261 (103, 428)	15 (6, 26)	0 (0, 0)
PSE	1,598 (935, 2,272)	All-ages curve	30.80 (13.82, 45.67)	23.01 (5.25, 40.23)	14.67 (1.14, 34.29)	3.42 (0.06, 20.53)	0.84 (0.00, 9.48)	492 (167, 901)	368 (64, 783)	234 (15, 660)	55 (1, 358)	13 (0, 152)
PSE	1,598 (935, 2,272)	Zhang et al. 2021	16.95 (11.55, 21.70)	12.84 (7.15, 17.86)	8.51 (2.70, 13.86)	1.09 (0.04, 5.44)	0.03 (0.00, 0.70)	271 (141, 421)	205 (96, 340)	136 (41, 257)	17 (1, 94)	0 (0, 12)

ISO	Total number of stillbirths	Method	Fraction of stillbirths attributable to $PM_{2.5} > C_0$ μg/m^3					Number of stillbirths attributable to $PM_{2.5} > C_0$ μg/m^3				
			$C_0 = 5$	$C_0 = 10$	$C_0 = 15$	$C_0 = 25$	$C_0 = 35$	$C_0 = 5$	$C_0 = 10$	$C_0 = 15$	$C_0 = 25$	$C_0 = 35$
PSE	1,598 (935, 2,272)	Xie et al. 2021	23.18 (8.94, 36.38)	17.59 (2.94, 31.67)	11.59 (0.66, 26.58)	2.93 (0.05, 15.42)	0.59 (0.00, 6.77)	370 (112, 685)	281 (45, 594)	185 (11, 476)	47 (1, 261)	9 (0, 108)
QAT	151 (124, 179)	Age-specific curves	71.16 (56.17, 80.88)	68.13 (52.60, 78.16)	64.77 (49.83, 74.41)	57.06 (41.52, 69.17)	48.15 (31.53, 62.44)	107 (79, 135)	103 (76, 131)	98 (72, 125)	86 (61, 113)	73 (46, 100)
QAT	151 (124, 179)	All-ages curve	66.81 (48.13, 78.50)	63.30 (42.55, 76.20)	59.41 (36.34, 73.64)	50.48 (22.24, 67.77)	40.44 (10.74, 61.12)	101 (71, 131)	96 (63, 126)	90 (54, 121)	76 (35, 110)	61 (18, 96)
QAT	151 (124, 179)	Zhang et al. 2021	54.59 (43.10, 63.11)	52.34 (40.32, 61.28)	49.98 (37.39, 59.34)	44.88 (31.10, 55.18)	39.25 (24.16, 50.58)	82 (61, 103)	79 (57, 99)	75 (54, 96)	68 (45, 88)	59 (35, 80)
QAT	151 (124, 179)	Xie et al. 2021	67.44 (40.52, 83.35)	65.09 (36.13, 82.15)	62.56 (31.39, 80.85)	56.88 (20.83, 77.94)	50.26 (10.95, 74.56)	102 (59, 137)	98 (52, 135)	94 (45, 132)	86 (31, 126)	76 (16, 118)
RWA	6,951 (6,452, 7,452)	Age-specific curves	48.72 (28.58, 64.25)	43.20 (25.07, 57.86)	37.07 (21.40, 50.31)	22.91 (13.56, 31.57)	6.43 (4.02, 8.94)	3,387 (2,092, 4,494)	3,003 (1,846, 4,044)	2,577 (1,569, 3,512)	1,592 (971, 2,223)	447 (281, 627)
RWA	6,951 (6,452, 7,452)	All-ages curve	46.87 (27.03, 63.37)	41.06 (19.13, 59.43)	34.57 (10.61, 55.02)	19.84 (1.76, 44.87)	8.93 (0.18, 33.40)	3,258 (1,858, 4,425)	2,854 (1,314, 4,141)	2,403 (729, 3,836)	1,379 (121, 3,138)	621 (12, 2,320)
RWA	6,951 (6,452, 7,452)	Zhang et al. 2021	28.14 (20.41, 34.58)	24.62 (16.51, 31.33)	20.92 (12.44, 27.92)	12.97 (3.91, 20.56)	4.81 (0.18, 12.45)	1,956 (1,392, 2,437)	1,711 (1,132, 2,212)	1,454 (859, 1,975)	901 (271, 1,442)	334 (12, 875)
RWA	6,951 (6,452, 7,452)	Xie et al. 2021	37.55 (17.79, 54.00)	33.12 (11.78, 50.69)	28.35 (5.91, 47.11)	17.73 (0.91, 39.11)	7.91 (0.16, 29.82)	2,610 (1,187, 3,796)	2,302 (796, 3,559)	1,971 (400, 3,288)	1,233 (65, 2,708)	550 (10, 2,049)
SAU	3,439 (2,022, 4,842)	Age-specific curves	68.38 (52.37, 79.06)	64.96 (50.63, 75.25)	61.15 (47.66, 70.86)	52.34 (38.80, 61.57)	42.11 (28.39, 53.66)	2,352 (1,383, 3,453)	2,234 (1,306, 3,283)	2,103 (1,224, 3,108)	1,800 (1,018, 2,720)	1,448 (736, 2,251)
SAU	3,439 (2,022, 4,842)	All-ages curve	62.89 (43.42, 75.27)	58.90 (37.33, 72.62)	54.45 (30.70, 69.67)	44.26 (17.22, 62.90)	33.18 (7.54, 55.28)	2,163 (1,144, 3,233)	2,026 (1,083, 3,098)	1,872 (937, 2,935)	1,522 (560, 2,578)	1,141 (280, 2,183)
SAU	3,439 (2,022, 4,842)	Zhang et al. 2021	48.32 (37.61, 56.51)	45.77 (34.52, 54.36)	43.09 (31.29, 52.11)	37.31 (24.37, 47.24)	31.01 (17.15, 41.89)	1,662 (959, 2,478)	1,574 (900, 2,361)	1,482 (818, 2,257)	1,283 (676, 2,024)	1,066 (504, 1,745)

ISO	Total number of stillbirths	Method	Fraction of stillbirths attributable to $PM_{2.5} > C_0$ μg/m^3					Number of stillbirths attributable to $PM_{2.5} > C_0$ μg/m^3				
			$C_0 = 5$	$C_0 = 10$	$C_0 = 15$	$C_0 = 25$	$C_0 = 35$	$C_0 = 5$	$C_0 = 10$	$C_0 = 15$	$C_0 = 25$	$C_0 = 35$
SAU	3,439 (2,022, 4,842)	Xie et al. 2021	60.58 (35.04, 77.09)	57.69 (30.31, 75.42)	54.58 (25.27, 73.61)	47.63 (15.14, 69.55)	39.79 (7.52, 64.82)	2,083 (930, 3,024)	1,984 (815, 2,911)	1,877 (692, 2,812)	1,638 (436, 2,620)	1,368 (204, 2,398)
SDN	31,918 (21,675, 43,101)	Age-specific curves	47.55 (28.12, 62.69)	41.92 (24.62, 56.02)	35.68 (20.88, 48.15)	21.39 (13.40, 29.22)	7.25 (4.81, 9.66)	15,176 (7,706, 22,544)	13,380 (6,780, 20,106)	11,388 (5,832, 17,204)	6,826 (3,654, 10,397)	2,314 (1,268, 3,431)
SDN	31,918 (21,675, 43,101)	All-ages curve	46.09 (26.62, 62.35)	40.25 (18.78, 58.32)	33.73 (10.81, 53.84)	19.58 (2.30, 43.56)	9.14 (0.49, 32.37)	14,711 (7,317, 23,561)	12,846 (5,023, 21,639)	10,768 (3,018, 19,788)	6,251 (654, 15,374)	2,916 (127, 11,044)
SDN	31,918 (21,675, 43,101)	Zhang et al. 2021	27.75 (20.10, 34.14)	24.18 (16.15, 30.88)	20.44 (12.01, 27.47)	12.46 (4.09, 20.12)	5.16 (0.55, 12.29)	8,856 (5,200, 13,486)	7,719 (4,122, 12,030)	6,525 (3,145, 10,498)	3,976 (1,088, 7,365)	1,646 (153, 4,362)
SDN	31,918 (21,675, 43,101)	Xie et al. 2021	36.91 (17.54, 53.21)	32.30 (11.57, 49.79)	27.34 (6.09, 46.11)	16.65 (1.03, 37.84)	7.80 (0.29, 28.52)	11,781 (5,143, 19,708)	10,311 (3,303, 17,917)	8,727 (1,755, 16,075)	5,314 (337, 12,744)	2,490 (92, 9,075)
SEN	10,951 (10,158, 11,876)	Age-specific curves	55.13 (34.70, 69.38)	50.31 (32.11, 64.15)	44.95 (29.10, 57.89)	32.63 (21.56, 42.73)	18.37 (12.31, 23.79)	6,038 (3,855, 7,788)	5,509 (3,529, 7,172)	4,923 (3,156, 6,447)	3,573 (2,298, 4,762)	2,011 (1,343, 2,716)
SEN	10,951 (10,158, 11,876)	All-ages curve	52.82 (31.87, 68.10)	47.55 (24.62, 64.64)	41.66 (16.59, 60.79)	28.19 (4.51, 51.91)	15.10 (1.02, 42.02)	5,785 (3,444, 7,500)	5,208 (2,665, 7,095)	4,562 (1,798, 6,637)	3,087 (491, 5,699)	1,653 (111, 4,630)
SEN	10,951 (10,158, 11,876)	Zhang et al. 2021	33.81 (25.06, 41.00)	30.54 (21.36, 38.10)	27.10 (17.49, 35.05)	19.69 (9.16, 28.48)	11.59 (2.23, 21.24)	3,702 (2,681, 4,469)	3,344 (2,277, 4,138)	2,967 (1,867, 3,815)	2,156 (995, 3,085)	1,269 (239, 2,284)
SEN	10,951 (10,158, 11,876)	Xie et al. 2021	44.37 (22.48, 61.65)	40.33 (16.85, 58.87)	35.96 (10.92, 55.86)	26.19 (2.82, 49.10)	15.93 (0.65, 41.22)	4,859 (2,395, 6,901)	4,416 (1,821, 6,582)	3,938 (1,195, 6,252)	2,868 (309, 5,436)	1,744 (71, 4,478)
SGP	109 (99, 119)	Age-specific curves	35.63 (17.13, 52.08)	28.95 (13.97, 43.33)	21.53 (10.46, 32.94)	4.24 (2.15, 6.69)	0.00 (0.00, 0.00)	39 (19, 56)	32 (15, 47)	23 (11, 35)	5 (2, 7)	0 (0, 0)
SGP	109 (99, 119)	All-ages curve	36.06 (16.29, 50.52)	30.01 (6.51, 45.38)	23.32 (1.80, 39.76)	9.48 (0.06, 26.99)	4.15 (0.00, 14.67)	39 (18, 57)	33 (7, 51)	25 (2, 44)	10 (0, 30)	5 (0, 16)
SGP	109 (99, 119)	Zhang et al. 2021	19.71 (13.61, 24.83)	15.83 (9.33, 21.15)	11.77 (4.77, 17.31)	3.35 (0.07, 8.91)	0.07 (0.00, 1.87)	21 (15, 27)	17 (10, 23)	13 (5, 19)	4 (0, 10)	0 (0, 2)

ISO	Total number of stillbirths	Method	Fraction of stillbirths attributable to $PM_{2.5} > C_0$ µg/m^3					Number of stillbirths attributable to $PM_{2.5} > C_0$ µg/m^3				
			$C_0 = 5$	$C_0 = 10$	$C_0 = 15$	$C_0 = 25$	$C_0 = 35$	$C_0 = 5$	$C_0 = 10$	$C_0 = 15$	$C_0 = 25$	$C_0 = 35$
SGP	109 (99, 119)	Xie et al. 2021	26.35 (10.53, 40.73)	20.63 (4.33, 36.32)	14.46 (1.05, 31.61)	4.98 (0.06, 21.11)	1.59 (0.00, 11.33)	29 (11, 44)	22 (4, 39)	16 (1, 34)	5 (0, 23)	2 (0, 12)
SLB	221 (123, 319)	Age-specific curves	12.42 (6.34, 19.06)	2.80 (1.40, 4.42)	0.00 (0.00, 0.09)	0.00 (0.00, 0.11)	0.00 (0.00, 0.00)	27 (11, 50)	6 (2, 12)	0 (0, 0)	0 (0, 0)	0 (0, 0)
SLB	221 (123, 319)	All-ages curve	12.15 (2.16, 21.99)	3.71 (0.00, 13.61)	0.62 (0.00, 7.01)	0.00 (0.00, 1.80)	0.00 (0.00, 0.58)	27 (4, 55)	8 (0, 31)	1 (0, 15)	0 (0, 4)	0 (0, 1)
SLB	221 (123, 319)	Zhang et al. 2021	5.91 (3.31, 8.76)	1.20 (0.05, 4.25)	0.00 (0.00, 0.68)	0.00 (0.00, 0.00)	0.00 (0.00, 0.00)	13 (6, 22)	3 (0, 10)	0 (0, 1)	0 (0, 0)	0 (0, 0)
SLB	221 (123, 319)	Xie et al. 2021	8.65 (1.13, 16.34)	2.60 (0.00, 10.14)	0.04 (0.00, 4.70)	0.00 (0.00, 1.40)	0.00 (0.00, 0.89)	19 (2, 42)	6 (0, 26)	0 (0, 11)	0 (0, 3)	0 (0, 2)
SLE	6,409 (3,576, 9,115)	Age-specific curves	38.40 (21.09, 53.52)	31.78 (17.32, 45.27)	24.44 (13.29, 35.49)	7.59 (4.17, 11.27)	0.00 (0.00, 0.00)	2,461 (1,087, 3,698)	2,037 (901, 3,118)	1,566 (694, 2,445)	486 (217, 780)	0 (0, 0)
SLE	6,409 (3,576, 9,115)	All-ages curve	37.47 (18.91, 53.05)	30.75 (10.36, 48.27)	23.29 (3.70, 42.97)	8.27 (0.20, 30.33)	1.10 (0.00, 17.77)	2,402 (902, 3,873)	1,971 (525, 3,452)	1,493 (160, 2,999)	530 (11, 2,071)	70 (0, 1,147)
SLE	6,409 (3,576, 9,115)	Zhang et al. 2021	21.15 (14.71, 26.50)	17.34 (10.45, 22.87)	13.34 (5.98, 19.06)	4.90 (0.26, 10.92)	0.86 (0.00, 3.01)	1,356 (665, 1,974)	1,111 (497, 1,672)	855 (288, 1,348)	314 (15, 687)	55 (0, 181)
SLE	6,409 (3,576, 9,115)	Xie et al. 2021	28.23 (12.31, 43.07)	22.66 (6.11, 38.71)	16.63 (1.83, 34.12)	5.92 (0.17, 24.02)	1.97 (0.00, 13.16)	1,809 (594, 3,061)	1,452 (289, 2,705)	1,066 (90, 2,346)	380 (10, 1,619)	126 (0, 856)
SLV	1,316 (935, 1,675)	Age-specific curves	38.48 (21.34, 53.21)	31.88 (17.40, 44.99)	24.56 (13.24, 35.24)	8.19 (4.57, 11.93)	0.01 (0.01, 0.02)	506 (254, 764)	419 (209, 642)	323 (159, 501)	108 (52, 171)	0 (0, 0)
SLV	1,316 (935, 1,675)	All-ages curve	37.94 (18.98, 53.12)	31.53 (10.26, 48.25)	24.41 (3.67, 42.83)	10.59 (0.34, 30.48)	3.48 (0.03, 18.12)	499 (226, 808)	415 (119, 719)	321 (46, 623)	139 (4, 432)	46 (0, 248)
SLV	1,316 (935, 1,675)	Zhang et al. 2021	21.26 (14.92, 26.73)	17.35 (10.71, 23.11)	13.23 (6.31, 19.31)	4.74 (0.54, 11.15)	0.43 (0.00, 3.60)	280 (166, 402)	228 (126, 345)	174 (77, 285)	62 (6, 159)	6 (0, 51)

ISO	Total number of stillbirths	Method	Fraction of stillbirths attributable to $PM_{2.5} > C_0$ μg/m^3					Number of stillbirths attributable to $PM_{2.5} > C_0$ μg/m^3				
			$C_0 = 5$	$C_0 = 10$	$C_0 = 15$	$C_0 = 25$	$C_0 = 35$	$C_0 = 5$	$C_0 = 10$	$C_0 = 15$	$C_0 = 25$	$C_0 = 35$
SLV	1,316 (935, 1,675)	Xie et al. 2021	28.87 (12.37, 43.57)	23.76 (6.20, 39.48)	18.28 (2.16, 35.07)	7.86 (0.25, 25.07)	2.46 (0.06, 14.82)	380 (138, 590)	313 (70, 522)	241 (22, 452)	103 (4, 311)	32 (1, 178)
SOM	16,635 (9,680, 23,718)	Age-specific curves	24.62 (13.48, 35.56)	16.34 (8.86, 23.94)	8.23 (4.54, 11.99)	2.08 (1.26, 2.99)	0.16 (0.11, 0.22)	4,095 (1,766, 6,778)	2,719 (1,177, 4,542)	1,370 (591, 2,264)	347 (168, 562)	26 (13, 43)
SOM	16,635 (9,680, 23,718)	All-ages curve	23.57 (9.40, 36.16)	15.42 (3.45, 29.40)	8.76 (1.09, 22.20)	3.10 (0.13, 11.11)	1.15 (0.02, 5.67)	3,921 (1,169, 7,177)	2,565 (464, 5,489)	1,457 (170, 3,913)	516 (17, 1,877)	191 (3, 962)
SOM	16,635 (9,680, 23,718)	Zhang et al. 2021	12.88 (8.36, 16.71)	8.66 (4.04, 12.60)	4.82 (1.79, 8.40)	1.32 (0.20, 3.21)	0.20 (0.01, 1.04)	2,142 (1,054, 3,271)	1,441 (527, 2,296)	803 (232, 1,418)	219 (28, 520)	34 (2, 156)
SOM	16,635 (9,680, 23,718)	Xie et al. 2021	17.61 (6.00, 28.71)	11.83 (1.99, 23.49)	7.16 (0.66, 17.98)	2.52 (0.11, 9.43)	1.05 (0.03, 5.01)	2,929 (804, 5,281)	1,968 (316, 4,187)	1,192 (105, 3,021)	419 (16, 1,585)	174 (4, 828)
SSD	11,379 (6,367, 16,113)	Age-specific curves	33.28 (18.10, 47.15)	26.00 (13.99, 37.69)	17.92 (9.60, 26.54)	2.05 (1.13, 3.06)	0.01 (0.01, 0.01)	3,786 (1,775, 6,423)	2,959 (1,387, 5,120)	2,040 (959, 3,586)	234 (115, 416)	1 (1, 2)
SSD	11,379 (6,367, 16,113)	All-ages curve	32.03 (14.90, 46.74)	24.54 (6.50, 41.11)	16.32 (1.81, 34.86)	4.80 (0.11, 21.18)	1.08 (0.01, 10.15)	3,644 (1,381, 6,643)	2,793 (572, 5,593)	1,857 (156, 4,641)	546 (9, 2,723)	123 (1, 1,253)
SSD	11,379 (6,367, 16,113)	Zhang et al. 2021	17.67 (12.12, 22.48)	13.61 (7.82, 18.63)	9.34 (3.46, 14.60)	1.89 (0.08, 6.24)	0.12 (0.00, 1.02)	2,011 (1,030, 3,181)	1,549 (629, 2,554)	1,063 (300, 1,937)	215 (9, 781)	14 (0, 121)
SSD	11,379 (6,367, 16,113)	Xie et al. 2021	24.06 (9.60, 37.61)	18.48 (3.64, 33.06)	12.53 (0.88, 28.15)	3.42 (0.07, 17.39)	0.84 (0.03, 8.14)	2,737 (912, 4,664)	2,103 (358, 4,020)	1,425 (90, 3,255)	389 (8, 1,984)	96 (3, 911)
STP	90 (52, 129)	Age-specific curves	25.93 (13.98, 37.82)	17.81 (9.45, 26.59)	8.78 (4.61, 13.34)	0.25 (0.15, 0.39)	0.00 (0.00, 0.00)	23 (10, 40)	16 (7, 28)	8 (3, 14)	0 (0, 0)	0 (0, 0)
STP	90 (52, 129)	All-ages curve	25.62 (9.49, 38.17)	18.36 (2.52, 31.76)	10.57 (0.46, 24.98)	2.70 (0.03, 11.82)	1.03 (0.00, 4.41)	23 (8, 42)	17 (2, 34)	10 (0, 26)	2 (0, 11)	1 (0, 4)
STP	90 (52, 129)	Zhang et al. 2021	13.27 (8.74, 17.28)	8.91 (4.33, 13.23)	4.48 (1.05, 8.95)	0.33 (0.01, 1.92)	0.00 (0.00, 0.20)	12 (6, 19)	8 (3, 14)	4 (1, 9)	0 (0, 2)	0 (0, 0)

ISO	Total number of stillbirths	Method	Fraction of stillbirths attributable to $PM_{2.5} > C_0$ μg/m^3					Number of stillbirths attributable to $PM_{2.5} > C_0$ μg/m^3				
			$C_0 = 5$	$C_0 = 10$	$C_0 = 15$	$C_0 = 25$	$C_0 = 35$	$C_0 = 5$	$C_0 = 10$	$C_0 = 15$	$C_0 = 25$	$C_0 = 35$
STP	90 (52, 129)	Xie et al. 2021	18.18 (5.99, 29.91)	12.08 (1.38, 24.76)	6.34 (0.29, 19.25)	1.41 (0.04, 8.92)	0.22 (0.01, 3.38)	16 (5, 30)	11 (1, 24)	6 (0, 18)	1 (0, 9)	0 (0, 3)
SUR	129 (80, 178)	Age-specific curves	26.17 (13.55, 38.44)	18.26 (9.29, 27.51)	9.48 (4.76, 14.64)	0.00 (0.00, 0.00)	0.00 (0.00, 0.00)	34 (13, 56)	24 (9, 40)	12 (5, 21)	0 (0, 0)	0 (0, 0)
SUR	129 (80, 178)	All-ages curve	25.39 (9.96, 39.25)	17.09 (2.48, 33.27)	8.47 (0.24, 25.98)	0.88 (0.00, 12.33)	0.04 (0.00, 4.25)	33 (9, 59)	22 (2, 48)	11 (0, 37)	1 (0, 15)	0 (0, 5)
SUR	129 (80, 178)	Zhang et al. 2021	13.99 (8.96, 17.79)	10.06 (4.32, 13.78)	5.96 (0.59, 9.61)	0.84 (0.00, 1.71)	0.00 (0.00, 0.07)	18 (9, 26)	13 (5, 19)	8 (1, 12)	1 (0, 2)	0 (0, 0)
SUR	129 (80, 178)	Xie et al. 2021	18.83 (6.53, 30.45)	12.93 (1.43, 25.25)	7.13 (0.20, 19.33)	1.65 (0.00, 8.22)	0.46 (0.00, 3.07)	24 (6, 45)	17 (1, 37)	9 (0, 28)	2 (0, 12)	1 (0, 4)
SWZ	428 (296, 555)	Age-specific curves	23.42 (12.05, 34.47)	15.16 (7.65, 22.86)	6.12 (3.09, 9.41)	0.00 (0.00, 0.01)	0.00 (0.00, 0.00)	100 (45, 164)	65 (28, 108)	26 (11, 44)	0 (0, 0)	0 (0, 0)
SWZ	428 (296, 555)	All-ages curve	23.25 (8.21, 35.68)	15.45 (1.69, 29.08)	8.00 (0.28, 21.77)	1.39 (0.00, 8.64)	0.61 (0.00, 2.91)	99 (29, 166)	66 (6, 130)	34 (1, 96)	6 (0, 38)	3 (0, 13)
SWZ	428 (296, 555)	Zhang et al. 2021	12.09 (7.78, 15.87)	7.73 (3.27, 11.74)	3.36 (0.47, 7.41)	0.07 (0.00, 1.05)	0.00 (0.00, 0.05)	52 (31, 75)	33 (13, 54)	14 (2, 34)	0 (0, 5)	0 (0, 0)
SWZ	428 (296, 555)	Xie et al. 2021	16.60 (5.27, 27.69)	10.46 (0.87, 22.29)	5.02 (0.17, 16.55)	1.05 (0.02, 6.78)	0.36 (0.00, 2.29)	71 (19, 129)	45 (3, 101)	21 (1, 73)	5 (0, 29)	2 (0, 10)
SYC	16 (13, 19)	Age-specific curves	8.17 (4.11, 12.91)	0.04 (0.02, 0.06)	0.00 (0.00, 0.16)	0.00 (0.00, 0.21)	0.00 (0.00, 0.00)	1 (1, 2)	0 (0, 0)	0 (0, 0)	0 (0, 0)	0 (0, 0)
SYC	16 (13, 19)	All-ages curve	7.74 (0.71, 16.69)	1.26 (0.00, 7.85)	0.17 (0.00, 2.69)	0.00 (0.00, 0.84)	0.00 (0.00, 0.15)	1 (0, 3)	0 (0, 1)	0 (0, 0)	0 (0, 0)	0 (0, 0)
SYC	16 (13, 19)	Zhang et al. 2021	4.09 (1.62, 6.20)	0.24 (0.00, 1.70)	0.00 (0.00, 0.10)	0.00 (0.00, 0.00)	0.00 (0.00, 0.00)	1 (0, 1)	0 (0, 0)	0 (0, 0)	0 (0, 0)	0 (0, 0)

ISO	Total number of stillbirths	Method	Fraction of stillbirths attributable to $PM_{2.5} > C_0$ μg/m^3					Number of stillbirths attributable to $PM_{2.5} > C_0$ μg/m^3				
			$C_0 = 5$	$C_0 = 10$	$C_0 = 15$	$C_0 = 25$	$C_0 = 35$	$C_0 = 5$	$C_0 = 10$	$C_0 = 15$	$C_0 = 25$	$C_0 = 35$
SYC	16 (13, 19)	Xie et al. 2021	5.41 (0.42, 12.01)	1.11 (0.00, 5.70)	0.38 (0.00, 1.68)	0.15 (0.00, 0.59)	0.00 (0.00, 0.33)	1 (0, 2)	0 (0, 1)	0 (0, 0)	0 (0, 0)	0 (0, 0)
SYR	5,019 (3,021, 7,094)	Age-specific curves	44.44 (25.39, 59.87)	38.54 (21.82, 52.72)	31.99 (18.06, 44.29)	16.96 (10.08, 23.69)	4.35 (2.82, 5.95)	2,230 (1,023, 3,477)	1,934 (889, 3,042)	1,606 (743, 2,544)	851 (410, 1,359)	218 (115, 345)
SYR	5,019 (3,021, 7,094)	All-ages curve	43.56 (24.41, 59.57)	37.60 (16.27, 55.27)	30.97 (8.63, 50.48)	17.02 (1.35, 39.50)	7.82 (0.23, 27.74)	2,186 (890, 3,479)	1,887 (609, 3,206)	1,554 (346, 2,870)	854 (67, 2,176)	392 (13, 1,450)
SYR	5,019 (3,021, 7,094)	Zhang et al. 2021	25.60 (18.36, 31.66)	21.95 (14.33, 28.28)	18.11 (10.09, 24.72)	9.93 (2.65, 17.06)	3.26 (0.23, 9.11)	1,285 (699, 1,853)	1,102 (563, 1,633)	909 (424, 1,416)	498 (117, 953)	164 (10, 482)
SYR	5,019 (3,021, 7,094)	Xie et al. 2021	34.20 (15.70, 50.05)	29.38 (9.48, 46.39)	24.18 (4.31, 42.42)	13.47 (0.71, 33.52)	5.98 (0.24, 23.69)	1,717 (686, 2,721)	1,475 (419, 2,491)	1,213 (208, 2,243)	676 (33, 1,703)	300 (11, 1,132)
TCD	18,009 (10,916, 25,485)	Age-specific curves	49.90 (31.57, 63.86)	44.52 (28.02, 57.67)	38.56 (24.92, 50.43)	24.90 (17.16, 32.71)	11.20 (7.52, 14.36)	8,986 (3,966, 13,649)	8,018 (3,563, 12,280)	6,944 (3,137, 10,781)	4,485 (2,212, 6,976)	2,017 (1,000, 3,071)
TCD	18,009 (10,916, 25,485)	All-ages curve	48.71 (28.88, 64.27)	43.25 (21.24, 60.48)	37.18 (13.38, 56.26)	23.70 (3.98, 46.58)	12.79 (1.41, 35.74)	8,772 (3,324, 13,455)	7,789 (2,476, 12,276)	6,695 (1,680, 11,015)	4,267 (644, 8,388)	2,303 (196, 6,165)
TCD	18,009 (10,916, 25,485)	Zhang et al. 2021	30.60 (22.55, 37.28)	27.16 (18.74, 34.19)	23.54 (14.74, 30.94)	15.76 (6.94, 23.94)	8.43 (2.72, 16.35)	5,511 (2,842, 7,991)	4,891 (2,395, 7,199)	4,239 (1,930, 6,350)	2,838 (928, 4,658)	1,518 (385, 2,968)
TCD	18,009 (10,916, 25,485)	Xie et al. 2021	40.22 (19.95, 56.64)	35.82 (14.10, 53.43)	31.07 (8.58, 49.97)	20.94 (2.81, 42.18)	12.43 (1.04, 33.36)	7,243 (3,197, 11,504)	6,451 (2,293, 10,498)	5,595 (1,519, 9,685)	3,770 (483, 8,036)	2,238 (188, 6,325)
TGO	6,050 (3,489, 8,563)	Age-specific curves	48.23 (28.48, 63.19)	42.64 (25.03, 56.58)	36.43 (21.36, 48.87)	22.14 (13.84, 29.85)	8.36 (5.70, 10.92)	2,918 (1,519, 4,777)	2,580 (1,356, 4,256)	2,204 (1,175, 3,681)	1,340 (739, 2,246)	506 (284, 822)
TGO	6,050 (3,489, 8,563)	All-ages curve	46.58 (27.01, 62.77)	40.86 (19.19, 58.73)	34.50 (11.32, 54.23)	20.30 (2.45, 43.88)	9.59 (0.59, 32.63)	2,818 (1,191, 4,613)	2,472 (823, 4,187)	2,087 (518, 3,792)	1,228 (149, 3,030)	580 (31, 2,128)
TGO	6,050 (3,489, 8,563)	Zhang et al. 2021	28.21 (20.52, 34.68)	24.67 (16.61, 31.46)	20.94 (12.51, 28.09)	12.93 (4.62, 20.82)	5.73 (0.90, 12.92)	1,707 (964, 2,646)	1,492 (803, 2,379)	1,267 (613, 2,073)	782 (249, 1,455)	346 (40, 867)

ISO	Total number of stillbirths	Method	Fraction of stillbirths attributable to $PM_{2.5} > C_0$ μg/m^3					Number of stillbirths attributable to $PM_{2.5} > C_0$ μg/m^3				
			$C_0 = 5$	$C_0 = 10$	$C_0 = 15$	$C_0 = 25$	$C_0 = 35$	$C_0 = 5$	$C_0 = 10$	$C_0 = 15$	$C_0 = 25$	$C_0 = 35$
TGO	6,050 (3,489, 8,563)	Xie et al. 2021	37.48 (17.96, 53.83)	32.92 (12.02, 50.47)	27.99 (6.46, 46.84)	17.60 (1.31, 38.69)	9.44 (0.30, 29.45)	2,268 (907, 3,890)	1,992 (606, 3,511)	1,694 (368, 3,194)	1,065 (83, 2,540)	571 (25, 1,897)
THA	4,748 (2,899, 6,637)	Age-specific curves	30.58 (16.12, 44.17)	23.16 (12.08, 34.20)	14.93 (7.76, 22.43)	1.87 (1.03, 2.77)	0.01 (0.00, 0.01)	1,452 (715, 2,395)	1,100 (535, 1,852)	709 (344, 1,215)	89 (45, 149)	0 (0, 1)
THA	4,748 (2,899, 6,637)	All-ages curve	30.26 (13.11, 44.24)	23.02 (4.96, 38.32)	15.25 (1.30, 31.75)	4.87 (0.09, 18.15)	1.42 (0.01, 8.40)	1,437 (445, 2,538)	1,093 (187, 2,084)	724 (55, 1,661)	231 (4, 814)	67 (0, 399)
THA	4,748 (2,899, 6,637)	Zhang et al. 2021	16.37 (11.09, 20.96)	12.24 (6.70, 17.06)	7.90 (2.58, 12.97)	1.38 (0.10, 4.81)	0.11 (0.00, 0.92)	777 (407, 1,189)	581 (266, 933)	375 (117, 686)	66 (5, 245)	5 (0, 45)
THA	4,748 (2,899, 6,637)	Xie et al. 2021	22.38 (8.43, 35.29)	16.74 (2.96, 30.53)	10.94 (0.85, 25.40)	3.30 (0.11, 14.59)	0.79 (0.06, 6.65)	1,062 (297, 1,898)	795 (109, 1,597)	520 (31, 1,299)	157 (5, 692)	38 (2, 306)
TJK	2,589 (1,474, 3,647)	Age-specific curves	41.39 (23.82, 55.89)	35.04 (20.02, 48.19)	28.14 (16.06, 39.35)	13.45 (7.66, 19.19)	2.44 (1.56, 3.37)	1,072 (483, 1,726)	907 (404, 1,468)	729 (322, 1,181)	348 (157, 557)	63 (32, 101)
TJK	2,589 (1,474, 3,647)	All-ages curve	40.34 (21.91, 55.99)	33.93 (13.87, 51.32)	26.98 (6.92, 46.18)	13.30 (1.19, 34.90)	5.14 (0.23, 23.71)	1,044 (379, 1,743)	879 (251, 1,572)	698 (129, 1,391)	344 (24, 989)	133 (5, 657)
TJK	2,589 (1,474, 3,647)	Zhang et al. 2021	23.45 (16.67, 29.18)	19.69 (12.59, 25.68)	15.80 (8.44, 22.02)	8.01 (1.84, 14.47)	2.40 (0.17, 7.17)	607 (325, 906)	510 (257, 779)	409 (193, 649)	207 (45, 393)	62 (4, 183)
TJK	2,589 (1,474, 3,647)	Xie et al. 2021	31.36 (14.06, 46.63)	26.26 (8.02, 42.68)	20.87 (3.30, 38.45)	10.42 (0.52, 29.23)	4.20 (0.16, 19.57)	812 (262, 1,332)	680 (148, 1,197)	540 (63, 1,068)	270 (9, 814)	109 (3, 536)
TKM	1,249 (749, 1,760)	Age-specific curves	41.35 (23.37, 56.23)	35.03 (19.66, 48.48)	28.03 (15.71, 39.49)	12.32 (6.99, 17.68)	1.94 (1.24, 2.69)	516 (244, 816)	438 (206, 701)	350 (165, 569)	154 (77, 254)	24 (12, 39)
TKM	1,249 (749, 1,760)	All-ages curve	40.50 (21.59, 56.18)	34.21 (13.19, 51.56)	27.23 (6.25, 46.41)	13.37 (1.01, 34.66)	5.16 (0.23, 22.68)	506 (171, 817)	427 (92, 736)	340 (40, 641)	167 (7, 453)	64 (2, 287)
TKM	1,249 (749, 1,760)	Zhang et al. 2021	23.33 (16.56, 29.07)	19.54 (12.43, 25.56)	15.57 (8.10, 21.87)	7.33 (1.57, 13.95)	1.87 (0.13, 6.47)	291 (139, 442)	244 (107, 387)	194 (79, 324)	92 (16, 203)	23 (2, 87)

ISO	Total number of stillbirths	Method	Fraction of stillbirths attributable to $PM_{2.5} > C_0$ μg/m^3					Number of stillbirths attributable to $PM_{2.5} > C_0$ μg/m^3				
			$C_0 = 5$	$C_0 = 10$	$C_0 = 15$	$C_0 = 25$	$C_0 = 35$	$C_0 = 5$	$C_0 = 10$	$C_0 = 15$	$C_0 = 25$	$C_0 = 35$
TKM	1,249 (749, 1,760)	Xie et al. 2021	31.29 (13.91, 46.58)	26.18 (7.66, 42.61)	20.67 (3.12, 38.33)	10.16 (0.49, 28.70)	4.01 (0.13, 18.68)	391 (129, 672)	327 (77, 604)	258 (29, 532)	127 (5, 384)	50 (2, 242)
TLS	492 (294, 700)	Age-specific curves	14.69 (7.54, 22.50)	5.35 (2.71, 8.41)	0.00 (0.00, 0.05)	0.00 (0.00, 0.09)	0.00 (0.00, 0.00)	72 (34, 132)	26 (13, 49)	0 (0, 0)	0 (0, 0)	0 (0, 0)
TLS	492 (294, 700)	All-ages curve	14.30 (2.91, 24.75)	6.00 (0.10, 17.05)	1.24 (0.00, 8.66)	0.00 (0.00, 1.64)	0.00 (0.00, 0.74)	70 (14, 134)	29 (0, 80)	6 (0, 42)	0 (0, 8)	0 (0, 3)
TLS	492 (294, 700)	Zhang et al. 2021	7.30 (4.09, 10.12)	2.73 (0.33, 5.73)	0.21 (0.00, 1.67)	0.00 (0.00, 0.02)	0.00 (0.00, 0.00)	36 (17, 56)	13 (1, 29)	1 (0, 8)	0 (0, 0)	0 (0, 0)
TLS	492 (294, 700)	Xie et al. 2021	9.87 (1.83, 18.50)	3.68 (0.13, 12.37)	0.73 (0.00, 6.73)	0.01 (0.00, 1.35)	0.00 (0.00, 0.49)	49 (10, 101)	18 (1, 66)	4 (0, 35)	0 (0, 7)	0 (0, 3)
TTO	187 (157, 220)	Age-specific curves	22.31 (11.13, 33.56)	14.04 (6.88, 21.71)	5.01 (2.42, 7.93)	0.00 (0.00, 0.01)	0.00 (0.00, 0.00)	42 (20, 67)	26 (13, 43)	9 (4, 16)	0 (0, 0)	0 (0, 0)
TTO	187 (157, 220)	All-ages curve	21.99 (7.61, 34.72)	13.63 (0.84, 28.01)	6.58 (0.04, 20.19)	1.27 (0.00, 7.81)	0.44 (0.00, 2.54)	41 (14, 68)	25 (1, 54)	12 (0, 38)	2 (0, 13)	1 (0, 5)
TTO	187 (157, 220)	Zhang et al. 2021	11.61 (7.44, 15.31)	7.25 (2.87, 11.19)	2.79 (0.19, 6.76)	0.12 (0.00, 0.59)	0.00 (0.00, 0.00)	22 (12, 29)	14 (5, 21)	5 (0, 13)	0 (0, 1)	0 (0, 0)
TTO	187 (157, 220)	Xie et al. 2021	16.29 (4.80, 26.68)	10.52 (0.59, 21.24)	5.00 (0.09, 15.41)	1.19 (0.00, 5.24)	0.12 (0.00, 1.59)	30 (8, 54)	20 (1, 43)	9 (0, 32)	2 (0, 11)	0 (0, 3)
TUN	2,520 (2,369, 2,664)	Age-specific curves	25.72 (13.08, 38.93)	17.74 (8.94, 27.57)	8.89 (4.49, 14.13)	0.13 (0.08, 0.21)	0.00 (0.00, 0.01)	648 (325, 948)	447 (222, 671)	224 (111, 343)	3 (2, 5)	0 (0, 0)
TUN	2,520 (2,369, 2,664)	All-ages curve	24.86 (10.05, 38.41)	16.53 (2.67, 32.01)	8.34 (0.44, 25.01)	1.70 (0.01, 11.11)	0.45 (0.00, 4.02)	627 (242, 998)	417 (63, 828)	210 (10, 643)	43 (0, 286)	11 (0, 101)
TUN	2,520 (2,369, 2,664)	Zhang et al. 2021	13.40 (8.82, 17.41)	9.14 (4.36, 13.32)	4.74 (0.85, 9.05)	0.24 (0.01, 1.67)	0.01 (0.00, 0.12)	338 (219, 450)	230 (108, 346)	119 (21, 232)	6 (0, 43)	0 (0, 3)

ISO	Total number of stillbirths	Method	Fraction of stillbirths attributable to $PM_{2.5} > C_0$ μg/m^3					Number of stillbirths attributable to $PM_{2.5} > C_0$ μg/m^3				
			$C_0 = 5$	$C_0 = 10$	$C_0 = 15$	$C_0 = 25$	$C_0 = 35$	$C_0 = 5$	$C_0 = 10$	$C_0 = 15$	$C_0 = 25$	$C_0 = 35$
TUN	2,520 (2,369, 2,664)	Xie et al. 2021	18.51 (6.33, 30.22)	12.68 (1.39, 25.21)	6.96 (0.23, 19.83)	0.98 (0.02, 8.79)	0.42 (0.00, 2.87)	466 (159, 761)	320 (35, 635)	175 (6, 497)	25 (0, 221)	11 (0, 75)
TUR	6,333 (6,167, 6,524)	Age-specific curves	39.99 (22.09, 55.05)	33.59 (18.38, 47.02)	26.49 (14.51, 37.56)	11.01 (6.23, 15.65)	1.94 (1.26, 2.70)	2,533 (1,396, 3,482)	2,127 (1,162, 2,979)	1,677 (921, 2,386)	697 (396, 989)	123 (81, 171)
TUR	6,333 (6,167, 6,524)	All-ages curve	39.23 (20.62, 54.67)	32.79 (12.29, 49.83)	25.65 (5.68, 44.43)	12.25 (0.88, 32.19)	4.65 (0.16, 20.64)	2,485 (1,303, 3,455)	2,077 (776, 3,135)	1,624 (359, 2,787)	776 (55, 2,012)	295 (10, 1,290)
TUR	6,333 (6,167, 6,524)	Zhang et al. 2021	22.47 (15.89, 28.10)	18.63 (11.72, 24.55)	14.60 (7.41, 20.82)	6.51 (1.44, 12.86)	1.68 (0.10, 5.82)	1,423 (1,005, 1,791)	1,180 (740, 1,562)	925 (466, 1,325)	412 (90, 816)	107 (6, 370)
TUR	6,333 (6,167, 6,524)	Xie et al. 2021	30.24 (13.31, 45.25)	25.12 (7.17, 41.24)	19.62 (2.92, 36.90)	9.51 (0.47, 27.24)	3.81 (0.15, 17.45)	1,915 (836, 2,871)	1,591 (451, 2,619)	1,242 (185, 2,346)	602 (30, 1,736)	241 (9, 1,113)
TZA	40,166 (33,359, 47,151)	Age-specific curves	29.07 (15.84, 41.69)	21.37 (11.56, 31.29)	12.91 (6.95, 19.20)	1.68 (0.98, 2.44)	0.02 (0.01, 0.02)	11,676 (6,397, 16,983)	8,582 (4,652, 12,730)	5,186 (2,788, 7,782)	674 (391, 986)	6 (4, 9)
TZA	40,166 (33,359, 47,151)	All-ages curve	28.40 (12.00, 42.02)	20.92 (4.35, 35.88)	13.45 (1.26, 29.17)	3.72 (0.13, 15.79)	1.14 (0.01, 7.18)	11,406 (4,724, 17,384)	8,405 (1,761, 14,868)	5,402 (503, 11,958)	1,495 (50, 6,067)	458 (4, 2,679)
TZA	40,166 (33,359, 47,151)	Zhang et al. 2021	15.34 (10.29, 19.72)	11.16 (5.88, 15.74)	6.85 (2.20, 11.57)	1.24 (0.11, 4.14)	0.11 (0.00, 0.90)	6,160 (3,963, 8,164)	4,482 (2,312, 6,404)	2,751 (909, 4,716)	500 (46, 1,621)	43 (0, 350)
TZA	40,166 (33,359, 47,151)	Xie et al. 2021	20.94 (7.73, 33.43)	15.17 (2.51, 28.54)	9.45 (0.64, 23.28)	2.85 (0.10, 12.72)	0.97 (0.04, 5.85)	8,412 (2,991, 13,900)	6,093 (949, 11,661)	3,798 (239, 9,388)	1,144 (37, 4,983)	389 (15, 2,273)
UGA	30,318 (26,704, 33,932)	Age-specific curves	40.30 (23.22, 54.80)	33.82 (19.40, 46.71)	26.63 (15.33, 37.21)	11.74 (6.81, 16.57)	1.84 (1.20, 2.59)	12,218 (7,214, 16,721)	10,253 (5,974, 14,161)	8,073 (4,683, 11,244)	3,558 (2,179, 4,968)	558 (350, 794)
UGA	30,318 (26,704, 33,932)	All-ages curve	39.17 (20.74, 54.61)	32.65 (12.57, 49.73)	25.44 (6.07, 44.29)	12.23 (0.63, 32.15)	4.43 (0.08, 20.66)	11,877 (5,991, 17,358)	9,898 (3,721, 15,785)	7,713 (1,804, 13,817)	3,709 (185, 9,931)	1,344 (26, 6,386)
UGA	30,318 (26,704, 33,932)	Zhang et al. 2021	22.53 (15.95, 28.16)	18.70 (11.79, 24.63)	14.67 (7.53, 20.91)	6.85 (1.49, 13.09)	1.72 (0.06, 6.38)	6,831 (4,419, 8,853)	5,669 (3,284, 7,751)	4,449 (2,132, 6,582)	2,075 (454, 4,046)	520 (17, 1,963)

ISO	Total number of stillbirths	Method	Fraction of stillbirths attributable to $PM_{2.5} > C_0$ µg/m^3					Number of stillbirths attributable to $PM_{2.5} > C_0$ µg/m^3				
			$C_0 = 5$	$C_0 = 10$	$C_0 = 15$	$C_0 = 25$	$C_0 = 35$	$C_0 = 5$	$C_0 = 10$	$C_0 = 15$	$C_0 = 25$	$C_0 = 35$
UGA	30,318 (26,704, 33,932)	Xie et al. 2021	30.31 (13.37, 45.28)	25.21 (7.37, 41.26)	19.76 (3.03, 36.93)	9.88 (0.44, 27.40)	4.09 (0.11, 17.87)	9,190 (3,964, 13,716)	7,643 (2,218, 12,304)	5,992 (914, 11,037)	2,996 (132, 8,273)	1,240 (34, 5,465)
URY	234 (220, 247)	Age-specific curves	13.24 (6.78, 20.34)	3.95 (1.99, 6.24)	0.00 (0.00, 0.08)	0.00 (0.00, 0.10)	0.00 (0.00, 0.00)	31 (16, 48)	9 (5, 15)	0 (0, 0)	0 (0, 0)	0 (0, 0)
URY	234 (220, 247)	All-ages curve	12.50 (2.64, 22.65)	4.02 (0.11, 14.56)	1.26 (0.00, 7.29)	0.01 (0.00, 1.64)	0.00 (0.00, 0.60)	29 (6, 54)	9 (0, 35)	3 (0, 17)	0 (0, 4)	0 (0, 1)
URY	234 (220, 247)	Zhang et al. 2021	6.66 (3.62, 9.34)	2.16 (0.19, 4.88)	0.07 (0.00, 1.20)	0.00 (0.00, 0.00)	0.00 (0.00, 0.00)	16 (8, 22)	5 (0, 11)	0 (0, 3)	0 (0, 0)	0 (0, 0)
URY	234 (220, 247)	Xie et al. 2021	9.13 (1.48, 17.44)	3.05 (0.11, 11.52)	0.59 (0.00, 5.93)	0.02 (0.00, 0.93)	0.00 (0.00, 0.45)	21 (4, 41)	7 (0, 27)	1 (0, 14)	0 (0, 2)	0 (0, 1)
UZB	5,263 (3,136, 7,316)	Age-specific curves	43.50 (24.99, 58.48)	37.39 (21.32, 51.23)	30.62 (17.44, 42.69)	15.38 (8.79, 21.88)	3.21 (2.03, 4.42)	2,289 (1,047, 3,604)	1,968 (898, 3,121)	1,611 (739, 2,586)	810 (393, 1,327)	169 (86, 264)
UZB	5,263 (3,136, 7,316)	All-ages curve	42.58 (23.78, 58.53)	36.37 (15.65, 54.07)	29.45 (8.09, 49.16)	15.14 (1.45, 37.89)	6.42 (0.30, 26.08)	2,241 (993, 3,573)	1,914 (692, 3,210)	1,550 (358, 2,851)	797 (65, 2,141)	338 (13, 1,444)
UZB	5,263 (3,136, 7,316)	Zhang et al. 2021	24.98 (17.89, 30.98)	21.28 (13.85, 27.56)	17.38 (9.62, 23.97)	9.20 (2.50, 16.26)	2.95 (0.26, 8.52)	1,315 (687, 1,966)	1,120 (596, 1,722)	915 (435, 1,455)	484 (100, 938)	155 (10, 472)
UZB	5,263 (3,136, 7,316)	Xie et al. 2021	33.49 (15.30, 49.13)	28.66 (9.20, 45.41)	23.48 (4.25, 41.39)	12.87 (0.64, 32.39)	5.41 (0.17, 22.67)	1,762 (691, 2,973)	1,509 (425, 2,663)	1,236 (187, 2,355)	677 (32, 1,753)	285 (8, 1,202)
VCT	22 (19, 25)	Age-specific curves	20.57 (10.51, 30.88)	12.01 (6.02, 18.53)	2.58 (1.28, 4.09)	0.00 (0.00, 0.01)	0.00 (0.00, 0.00)	5 (2, 7)	3 (1, 4)	1 (0, 1)	0 (0, 0)	0 (0, 0)
VCT	22 (19, 25)	All-ages curve	19.80 (6.47, 32.29)	10.89 (0.86, 25.46)	3.08 (0.02, 17.37)	0.29 (0.00, 5.65)	0.00 (0.00, 1.77)	4 (1, 7)	2 (0, 6)	1 (0, 4)	0 (0, 1)	0 (0, 0)
VCT	22 (19, 25)	Zhang et al. 2021	10.77 (6.55, 14.00)	6.63 (1.88, 9.81)	2.51 (0.05, 5.39)	0.02 (0.00, 0.23)	0.00 (0.00, 0.00)	2 (1, 3)	1 (0, 2)	1 (0, 1)	0 (0, 0)	0 (0, 0)

ISO	Total number of stillbirths	Method	Fraction of stillbirths attributable to $PM_{2.5} > C_0$ μg/m^3					Number of stillbirths attributable to $PM_{2.5} > C_0$ μg/m^3				
			$C_0 = 5$	$C_0 = 10$	$C_0 = 15$	$C_0 = 25$	$C_0 = 35$	$C_0 = 5$	$C_0 = 10$	$C_0 = 15$	$C_0 = 25$	$C_0 = 35$
VCT	22 (19, 25)	Xie et al. 2021	14.32 (4.21, 24.77)	7.90 (0.50, 19.31)	3.19 (0.03, 13.37)	0.46 (0.00, 4.15)	0.00 (0.00, 1.51)	3 (1, 5)	2 (0, 4)	1 (0, 3)	0 (0, 1)	0 (0, 0)
VEN	5,258 (2,999, 7,410)	Age-specific curves	28.33 (15.00, 40.75)	20.68 (10.78, 30.43)	12.36 (6.39, 18.58)	0.41 (0.24, 0.63)	0.00 (0.00, 0.00)	1,490 (672, 2,413)	1,087 (483, 1,791)	650 (287, 1,087)	22 (10, 36)	0 (0, 0)
VEN	5,258 (2,999, 7,410)	All-ages curve	27.98 (11.59, 41.91)	20.35 (3.59, 35.92)	12.46 (0.64, 29.30)	2.82 (0.04, 15.23)	0.54 (0.00, 6.29)	1,471 (479, 2,681)	1,070 (139, 2,228)	655 (29, 1,794)	149 (2, 861)	28 (0, 344)
VEN	5,258 (2,999, 7,410)	Zhang et al. 2021	15.02 (10.07, 19.39)	10.78 (5.66, 15.42)	6.41 (1.76, 11.28)	0.63 (0.02, 3.37)	0.02 (0.00, 0.33)	790 (398, 1,213)	567 (229, 923)	337 (67, 664)	33 (1, 192)	1 (0, 17)
VEN	5,258 (2,999, 7,410)	Xie et al. 2021	20.71 (7.61, 33.08)	15.06 (2.32, 28.21)	9.28 (0.48, 22.89)	2.13 (0.05, 12.19)	0.33 (0.02, 4.86)	1,089 (333, 1,865)	792 (98, 1,582)	488 (22, 1,256)	112 (2, 620)	17 (1, 256)
VNM	13,633 (9,353, 18,165)	Age-specific curves	35.68 (19.98, 49.27)	28.78 (16.09, 40.30)	21.13 (11.91, 29.89)	8.69 (5.01, 12.32)	1.93 (1.24, 2.71)	4,865 (2,360, 7,482)	3,924 (1,893, 6,138)	2,881 (1,389, 4,558)	1,184 (614, 1,866)	264 (142, 398)
VNM	13,633 (9,353, 18,165)	All-ages curve	35.17 (17.70, 49.74)	28.41 (9.98, 44.46)	21.16 (4.85, 38.59)	9.91 (0.83, 26.30)	4.10 (0.21, 16.79)	4,794 (1,978, 7,945)	3,873 (1,099, 6,985)	2,885 (520, 5,944)	1,351 (88, 3,835)	558 (25, 2,305)
VNM	13,633 (9,353, 18,165)	Zhang et al. 2021	19.90 (13.91, 25.05)	15.93 (9.66, 21.34)	11.77 (5.81, 17.45)	5.23 (1.32, 9.82)	1.73 (0.08, 4.80)	2,713 (1,677, 3,866)	2,172 (1,221, 3,195)	1,605 (749, 2,529)	713 (176, 1,428)	235 (9, 677)
VNM	13,633 (9,353, 18,165)	Xie et al. 2021	26.92 (11.30, 40.87)	21.63 (5.70, 36.58)	16.23 (2.40, 31.96)	7.87 (0.36, 22.19)	3.11 (0.11, 14.14)	3,669 (1,447, 6,116)	2,948 (702, 5,387)	2,213 (291, 4,629)	1,072 (42, 3,019)	424 (13, 1,916)
YEM	20,512 (15,380, 25,427)	Age-specific curves	53.63 (34.21, 68.09)	48.58 (30.72, 62.53)	42.98 (27.68, 55.99)	30.15 (20.11, 39.81)	15.64 (10.62, 20.45)	11,000 (6,487, 15,265)	9,965 (5,842, 13,967)	8,816 (5,156, 12,438)	6,185 (3,710, 8,813)	3,208 (2,032, 4,647)
YEM	20,512 (15,380, 25,427)	All-ages curve	51.91 (31.23, 67.19)	46.78 (24.08, 63.62)	41.07 (16.17, 59.64)	28.04 (4.46, 50.52)	16.01 (1.10, 40.38)	10,648 (5,834, 14,903)	9,595 (4,498, 13,907)	8,423 (3,001, 12,910)	5,751 (816, 10,766)	3,283 (218, 8,786)
YEM	20,512 (15,380, 25,427)	Zhang et al. 2021	32.87 (24.30, 39.95)	29.56 (20.57, 37.01)	26.07 (16.65, 33.92)	18.58 (8.31, 27.27)	10.50 (2.19, 19.96)	6,743 (4,198, 9,394)	6,063 (3,650, 8,697)	5,348 (2,996, 7,965)	3,810 (1,533, 6,304)	2,154 (414, 4,397)

ISO	Total number of stillbirths	Method	Fraction of stillbirths attributable to $PM_{2.5} > C_0$ μg/m^3					Number of stillbirths attributable to $PM_{2.5} > C_0$ μg/m^3				
			$C_0 = 5$	$C_0 = 10$	$C_0 = 15$	$C_0 = 25$	$C_0 = 35$	$C_0 = 5$	$C_0 = 10$	$C_0 = 15$	$C_0 = 25$	$C_0 = 35$
YEM	20,512 (15,380, 25,427)	Xie et al. 2021	43.23 (21.64, 60.31)	39.14 (15.86, 57.43)	34.72 (9.82, 54.32)	24.86 (2.41, 47.34)	14.75 (0.67, 39.22)	8,868 (4,028, 13,695)	8,028 (2,984, 12,834)	7,122 (1,934, 11,945)	5,100 (502, 10,186)	3,026 (122, 8,418)
ZAF	20,415 (17,209, 24,025)	Age-specific curves	30.73 (18.27, 41.73)	23.96 (14.32, 32.54)	17.58 (10.68, 23.76)	9.10 (6.25, 11.93)	4.75 (3.04, 6.11)	6,273 (3,274, 8,615)	4,891 (2,644, 6,690)	3,590 (2,095, 4,872)	1,857 (1,204, 2,481)	970 (650, 1,318)
ZAF	20,415 (17,209, 24,025)	All-ages curve	29.92 (15.60, 42.97)	23.19 (9.31, 37.09)	17.21 (5.31, 31.34)	9.37 (1.48, 21.34)	5.02 (0.48, 14.86)	6,108 (2,967, 9,279)	4,735 (1,890, 7,873)	3,514 (1,120, 6,661)	1,914 (304, 4,433)	1,024 (92, 2,917)
ZAF	20,415 (17,209, 24,025)	Zhang et al. 2021	17.67 (12.33, 22.27)	13.91 (8.65, 18.59)	10.55 (5.84, 15.17)	6.02 (2.70, 9.61)	3.44 (1.03, 6.23)	3,607 (2,358, 4,888)	2,840 (1,691, 4,027)	2,153 (1,177, 3,274)	1,228 (559, 2,073)	701 (219, 1,343)
ZAF	20,415 (17,209, 24,025)	Xie et al. 2021	23.68 (10.10, 35.92)	18.74 (5.88, 31.39)	14.38 (3.41, 27.02)	8.27 (1.12, 19.28)	4.94 (0.37, 13.81)	4,835 (1,857, 7,589)	3,826 (1,045, 6,628)	2,936 (601, 5,721)	1,688 (196, 4,054)	1,008 (67, 2,845)
ZMB	9,615 (8,097, 11,134)	Age-specific curves	31.13 (16.73, 44.56)	23.72 (12.57, 34.73)	15.50 (8.18, 23.16)	1.29 (0.72, 1.95)	0.00 (0.00, 0.00)	2,993 (1,594, 4,283)	2,281 (1,199, 3,340)	1,491 (783, 2,228)	124 (70, 188)	0 (0, 0)
ZMB	9,615 (8,097, 11,134)	All-ages curve	30.53 (13.42, 44.84)	23.18 (5.09, 39.08)	15.24 (1.11, 32.68)	4.80 (0.08, 18.94)	1.42 (0.00, 8.70)	2,936 (1,140, 4,503)	2,228 (420, 3,918)	1,465 (104, 3,268)	462 (8, 1,888)	137 (0, 872)
ZMB	9,615 (8,097, 11,134)	Zhang et al. 2021	16.58 (11.28, 21.26)	12.44 (6.91, 17.40)	8.10 (2.78, 13.34)	1.31 (0.04, 5.18)	0.04 (0.00, 0.74)	1,594 (997, 2,071)	1,196 (615, 1,676)	779 (250, 1,271)	126 (4, 477)	3 (0, 70)
ZMB	9,615 (8,097, 11,134)	Xie et al. 2021	22.76 (8.73, 35.74)	17.22 (3.05, 31.03)	11.52 (0.82, 25.90)	3.42 (0.06, 15.25)	0.99 (0.02, 7.16)	2,188 (810, 3,563)	1,656 (280, 3,094)	1,107 (69, 2,588)	329 (5, 1,484)	95 (2, 678)
ZWE	8,392 (7,954, 8,797)	Age-specific curves	24.30 (12.34, 35.86)	16.22 (8.12, 24.54)	7.43 (3.71, 11.46)	0.00 (0.00, 0.01)	0.00 (0.00, 0.00)	2,039 (1,047, 2,986)	1,361 (688, 2,048)	623 (314, 959)	0 (0, 0)	0 (0, 0)
ZWE	8,392 (7,954, 8,797)	All-ages curve	23.83 (9.29, 36.87)	15.57 (2.30, 30.34)	7.55 (0.35, 23.16)	0.96 (0.01, 10.01)	0.10 (0.00, 3.22)	2,000 (773, 3,152)	1,306 (192, 2,589)	634 (29, 1,965)	81 (1, 839)	9 (0, 272)
ZWE	8,392 (7,954, 8,797)	Zhang et al. 2021	12.79 (8.25, 16.65)	8.56 (3.75, 12.53)	4.28 (0.61, 8.22)	0.19 (0.00, 1.28)	0.01 (0.00, 0.05)	1,074 (695, 1,414)	718 (315, 1,068)	359 (51, 700)	16 (0, 106)	1 (0, 4)

ISO	Total number of stillbirths	Method	Fraction of stillbirths attributable to $PM_{2.5} > C_0$ µg/m^3					Number of stillbirths attributable to $PM_{2.5} > C_0$ µg/m^3				
			$C_0 = 5$	$C_0 = 10$	$C_0 = 15$	$C_0 = 25$	$C_0 = 35$	$C_0 = 5$	$C_0 = 10$	$C_0 = 15$	$C_0 = 25$	$C_0 = 35$
ZWE	8,392 (7,954, 8,797)	Xie et al. 2021	17.55 (5.82, 28.90)	11.60 (1.08, 23.68)	6.08 (0.18, 18.06)	1.17 (0.02, 7.68)	0.17 (0.00, 2.60)	1,473 (488, 2,432)	973 (90, 1,998)	510 (16, 1,529)	98 (2, 650)	14 (0, 219)
