# Supplementary material for: Estimation of stillbirths attributable to ambient fine particles in 137 countries
Source: Nat Commun. 2022 Nov 29;13:6950. doi: 10.1038/s41467-022-34250-4 (PMC9709081; doi:10.1038/s41467-022-34250-4)
Supplement: Supplementary file 3 — Reporting Summary [file 41467_2022_34250_MOESM3_ESM.pdf]

nature portfolio

Corresponding author(s): Tao Xue

Last updated by author(s): Oct 8, 2022

Reporting Summary

Nature Portfolio wishes to improve the reproducibility of the work that we publish. This form provides structure for consistency and transparency in reporting. For further information on Nature Portfolio policies, see our Editorial Policies and the Editorial Policy Checklist.

Statistics

For all statistical analyses, confirm that the following items are present in the figure legend, table legend, main text, or Methods section.

n/a	Confirmed	
☐	☒	The exact sample size (*n*) for each experimental group/condition, given as a discrete number and unit of measurement
☐	☒	A statement on whether measurements were taken from distinct samples or whether the same sample was measured repeatedly
☐	☒	The statistical test(s) used AND whether they are one- or two-sided<br>*Only common tests should be described solely by name; describe more complex techniques in the Methods section.*
☒	☐	A description of all covariates tested
☒	☐	A description of any assumptions or corrections, such as tests of normality and adjustment for multiple comparisons
☐	☒	A full description of the statistical parameters including central tendency (e.g. means) or other basic estimates (e.g. regression coefficient) AND variation (e.g. standard deviation) or associated estimates of uncertainty (e.g. confidence intervals)
☐	☒	For null hypothesis testing, the test statistic (e.g. *F*, *t*, *r*) with confidence intervals, effect sizes, degrees of freedom and *P* value noted<br>*Give P values as exact values whenever suitable.*
☒	☐	For Bayesian analysis, information on the choice of priors and Markov chain Monte Carlo settings
☒	☐	For hierarchical and complex designs, identification of the appropriate level for tests and full reporting of outcomes
☒	☐	Estimates of effect sizes (e.g. Cohen's *d*, Pearson's *r*), indicating how they were calculated

Our web collection on statistics for biologists contains articles on many of the points above.

Software and code

Policy information about availability of computer code

Data collection	The data were directly downloaded from open sources. No software or code was involved in data collection.
Data analysis	The statistical data were analyzed using R software (version 4.0.2). Statistical inference for the regression models was performed using the R package survival (version 3.3-1). The codes to generate Figures are within the supplemental materials.

For manuscripts utilizing custom algorithms or software that are central to the research but not yet described in published literature, software must be made available to editors and reviewers. We strongly encourage code deposition in a community repository (e.g. GitHub). See the Nature Portfolio guidelines for submitting code & software for further information.

Data

Policy information about availability of data

All manuscripts must include a data availability statement. This statement should provide the following information, where applicable:

- Accession codes, unique identifiers, or web links for publicly available datasets
- A description of any restrictions on data availability
- For clinical datasets or third party data, please ensure that the statement adheres to our policy

The datasets used in this study are publicly available. The Demographic and Health Survey (DHS) dataset, PM2.5 dataset, MERRA-2, stillbirth number, and population datasets of pregnancies and age-specific females are available from https://www.dhsprogram.com/, https://sites.wustl.edu/acag/datasets/surface-pm2-5/, https://disc.gsfc.nasa.gov/datasets?project=MERRA-2, http://childmortality.org/, and https://www.worldpop.org/, respectively.

Human research participants

Policy information about studies involving human research participants and Sex and Gender in Research.

Reporting on sex and gender	There were no sex- and gender-based analyses have been performed.
Population characteristics	We analyzed 46,319 cases of gestation linked to 13,870 mothers from 1998 to 2016. The mean maternal age in the control group was 24.97 years with a standard deviation (SD) of 6.02 years, younger than the stillbirth group (mean 26.56 years; SD 7.02 years). The mean length of intervals between stillbirth and livebirth was 3.81 (SD = 2.45) years.
Recruitment	This study is an observational study based on the extent data collected by DHS fieldworkers. We utilized all available samples from the DHS database.
Ethics oversight	NA. This study is based on publicly available population data. We adhered to the DHS data usage guidelines in all our analyses. No further ethic approval is required.

Note that full information on the approval of the study protocol must also be provided in the manuscript.

Field-specific reporting

Please select the one below that is the best fit for your research. If you are not sure, read the appropriate sections before making your selection.

☐ Life sciences ☐ Behavioural & social sciences ☒ Ecological, evolutionary & environmental sciences

For a reference copy of the document with all sections, see nature.com/documents/nr-reporting-summary-flat.pdf

Ecological, evolutionary & environmental sciences study design

All studies must disclose on these points even when the disclosure is negative.

Study description	We developed age-specific exposure–response curves to assess the global risk assessment on PM2.5-related stillbirths. We combined state-of-the-art estimates on key inputs, namely, (1) total number of stillbirths by country, (2) gridded PM2.5 concentrations, (3) gridded demographic variables on total pregnancies and sex-age-specific populations, and (4) multiple exposure–response functions obtained from previous meta-analyses or derived by a self-matched case-control study of individual-level reproductive histories from 54 low- and middle-income countries.
Research sample	Valid records of pregnancy outcomes were obtained from the Demographic and Health Surveys (DHS) program from 1998 to 2016. All eligible samples aged 15-49 years old from 54 LMICs were finally involved in our study.
Sampling strategy	This study was based on extant DHS database. For each mother, to minimize recall bias, we incorporated only the most recent case of pregnancy loss and all available controls in the study period. No further sampling procedure was conducted.
Data collection	The DHS surveys are household-based instruments, and the samples were selected using a complex two-stage design. The females of reproductive age (15–49 years) in each household were of particular interest, and their records for socioeconomic status, fertility, reproductive history, infant mortality, etc. were screened by well trained interviewers using standard questionnaires. DHS utilized a team approach to data collection. Usually, each DHS team is composed of a supervisor, field editor, and several interviewers. The cases are recorded by the interviewers using the reproductive module of the uniform questionnaire. The geographic information are recorded by the Global Position System (GPS) device.
Timing and spatial scale	To estimate the exposure-response curves, we included all eligible samples during the period (i.e., 1998 - 2016) from 113 geocoded surveys of 54 low- and middle-income countries. To combine the curves with state-of-the-art estimates on the population at risk, PM2.5 concentration, and baseline risk, we evaluated the number of stillbirths attributable to PM2.5 exposure in 137 countries from 2000 to 2019.
Data exclusions	For each mother, we incorporated only the most recent case of pregnancy loss and all available controls in the study period. No data were excluded.
Reproducibility	All attempts to repeat the experiment were successful.
Randomization	NA. This study is an observational study, and we didn't perform further sampling or randomizing.
Blinding	This study is an observational, and thus is not applicable for a blinding design.

Did the study involve field work? ☐ Yes ☒ No

Reporting for specific materials, systems and methods

We require information from authors about some types of materials, experimental systems and methods used in many studies. Here, indicate whether each material, system or method listed is relevant to your study. If you are not sure if a list item applies to your research, read the appropriate section before selecting a response.

Materials & experimental systems

n/a	Involved in the study	
☒	☐	Antibodies
☒	☐	Eukaryotic cell lines
☒	☐	Palaeontology and archaeology
☒	☐	Animals and other organisms
☒	☐	Clinical data
☒	☐	Dual use research of concern

Methods

n/a	Involved in the study	
☒	☐	ChIP-seq
☒	☐	Flow cytometry
☒	☐	MRI-based neuroimaging
