# Supplementary material for: Estimation of stillbirths attributable to ambient fine particles in 137 countries
Source: Nat Commun. 2022 Nov 29;13:6950. doi: 10.1038/s41467-022-34250-4 (PMC9709081; doi:10.1038/s41467-022-34250-4)
Supplement: Supplementary file 4 — Description of Additional Supplementary Files [file 41467_2022_34250_MOESM4_ESM.pdf]

Description of Additional Supplementary Files

File Name: Supplementary Data 1
Description: A series of look-up tables represent the exposure-response functions estimated in the current study or obtained from previous meta-analyses.

File Name: Supplementary Data 2
Description: The R codes and relevant data for the epidemiological analyses and figures.
