# Supplementary material for: Estimation of stillbirths attributable to ambient fine particles in 137 countries
Source: Nat Commun. 2022 Nov 29;13:6950. doi: 10.1038/s41467-022-34250-4 (PMC9709081; doi:10.1038/s41467-022-34250-4)
Supplement: Supplementary file 6 — Supplementary Data 2 [file 41467_2022_34250_MOESM6_ESM.zip › Supp_code.html]

Supplemental R codes to reproduce the epidemiological models


# Supplemental R codes to reproduce the epidemiological models

#### Tao Xue, et al.

#### 2022/7/27

## Dataset introduction

The DHS data are owned by the third party, and thus cannot be distributed by the authors. For research purpose, the readers should download the datasets by countries from the DHS website: https://dhsprogram.com/, and manually combine the country-specific datasets as we described in the manuscript.

To reproduce the epidemiological models on the association between PM2.5 and stillbirth, we generate a mimic dataset after a series of random perturbation of the original inputs. First, we add a random error to all continuous variables, and the random errors follow a gaussian distribution with a mean of zero and a 5% standard deviation of the variable. Second, we random sample the mothers within each country with replacement. Third, we use pseudo-IDs for country and mother. With those perturbation, the key features (e.g., sample size) of original data are remained, but the mimic data cannot be utalized to identify the original dataset.

The mimic data are shown as follows.

```
load("Mimic analyzed DHS data on stillbirth.RData")
head(dta)
```

```
         end    x maternal.age multiparous  TMP end.m end.date  ntl cc  v2id
2220803    1 20.6           20           N 10.4     7     1363  0.0  1 11963
22208031   0 23.9           21           N 14.9     2     1394  0.6  1 11963
2213811    1 25.4           22           Y 12.7    12     1260  0.0  1 11967
22138111   0 32.7           32           Y  5.7     6     1278  1.0  1 11967
2219470    1 20.9           24           N 14.4     8     1364 21.5  1 11968
22194701   0 20.7           29           N 15.7    10     1378 21.4  1 11968
            age4       ntl3
2220803  [20,30)    [0,0.6]
22208031 [20,30)    [0,0.6]
2213811  [20,30)    [0,0.6]
22138111 [30,35)  (0.6,6.7]
2219470  [20,30) (6.7,64.4]
22194701 [20,30) (6.7,64.4]
```

Outcome and exposure:

- end: stillbirth (1) / livebirth (0)
- x: PM2.5

Covariates:

- maternal.age: maternal age
- multiparous: multiparous (Y) / nulliparous (N)
- TMP: ambient temperature
- end.m: calendar month of pregnancy end
- end.date: date of pregnancy end (in CMC month)
- ntl: nightlight
- cc: country ID
- v2id: mother ID

Effect modifier examples:

- age4: levels of maternal age
- ntl3: levels of nightlight

## Parameter selections for random effects

The heterogeneous effects by countries are modeled as random effects, so that we need to first decide the degree of shrink in the random terms. We select the set of parameters that can minimize the AIC criteria.

```
library(survival)
library(splines)
dta$one=1
f="Surv(one,end)~x+ns(maternal.age,3)+multiparous+ns(TMP,3)+ns(end.m,4)+ntl+strata(v2id)"
turning=NULL
thetas=expand.grid(theta1=10^c(-1:2),theta2=10^(-1:2))

  bs<-model.matrix(lm(end~cc:x-1,data=dta))
  colnames(bs)=paste("s",gsub("cc|:x","",fixed=F,colnames(bs)),sep="")
  dta$end.date=scale(dta$end.date)*sd(dta$x)
  bs1<-model.matrix(lm(end~cc:end.date-1,data=dta))
  colnames(bs1)=paste("t",gsub("cc|:end.date","",fixed=F,colnames(bs1)),sep="")

for(i in 1:dim(thetas)[1])
{
  m<-coxph(as.formula(paste(f,"+ridge(",paste(colnames(bs),collapse=","),",theta=",thetas$theta1[i],")",                        "+ridge(",paste(colnames(bs1),collapse=","),",theta=",thetas$theta2[i],")",sep="")),data=cbind(dta,bs,bs1),robust=T)
  turning<-rbind(turning,
                 cbind(theta=i,AIC=AIC(m),BIC=BIC(m),LL=logLik(m))
                )
  print(i)
}
```

```
[1] 1
[1] 2
[1] 3
[1] 4
[1] 5
[1] 6
[1] 7
[1] 8
[1] 9
[1] 10
[1] 11
[1] 12
[1] 13
[1] 14
[1] 15
[1] 16
```

```
turning=as.data.frame(cbind(thetas,turning))
library(lattice)
levelplot(AIC~log10(theta1)+log10(theta2),data=turning)
```

```
theta1=turning[which.min(turning$AIC),"theta1"]
theta2=turning[which.min(turning$AIC),"theta2"]
```

note:

- f: the full terms for fixed effects
- bs: the country-specific slopes for exposure
- bs1: the country-specific temporal trends in the baseline risk of stillbirth
- The conditional logit regression is statistically identical to the cox model with “Surv(1,end)”.
- thetas: the turning parameters
- In practice, more combinations of the turning parameters (thetas) are applied than those applied here. We set the thetas as above, in order to save the computational time.

## Linear models with different covariate adjustments

The following codes are for the linear association models. We apply a series of models, which sequentially included different sets of covariates.

```
fs=c("Surv(one,end)~x+strata(v2id)",
     "Surv(one,end)~x+ns(maternal.age,3)+multiparous+strata(v2id)",
     "Surv(one,end)~x+ns(maternal.age,3)+multiparous+ns(TMP,3)+ns(end.m,4)+strata(v2id)",
     "Surv(one,end)~x+ns(maternal.age,3)+multiparous+ns(TMP,3)+ns(end.m,4)+ntl+strata(v2id)"
)
coef=NULL
for(f in fs)
{
  m<-coxph(as.formula(f),data=dta,robust=T)
  coef<-rbind(coef,cbind(model=which(fs==f),as.data.frame(summary(m)$coef)["x",c("coef","se(coef)","robust se","Pr(>|z|)")]))
}
colnames(coef)=c("model","coef","se(coef)","se2","p")

m<-coxph(as.formula(paste(fs[4],"+ridge(",paste(colnames(bs1),collapse=","),",theta=",theta2,")",sep="")),data=cbind(dta,bs1),robust=T)
coef<-rbind(coef,cbind(model=5,as.data.frame(summary(m)$coef)["x",c("coef","se(coef)","se2","p")]))

m<-coxph(as.formula(paste(fs[4],"+ridge(",paste(colnames(bs),collapse=","),",theta=",theta1,")","+ridge(",paste(colnames(bs1),collapse=","),",theta=",theta2,")",sep="")),data=cbind(dta,bs,bs1),robust=T)
coef<-rbind(coef,cbind(model=6,as.data.frame(summary(m)$coef)["x",c("coef","se(coef)","se2","p")]))

coef
```

```
   model        coef    se(coef)         se2            p
x      1 0.023684952 0.001619234 0.001382368 8.333019e-66
x1     2 0.013848187 0.001734863 0.001432721 4.219220e-22
x2     3 0.009685827 0.001883652 0.001539977 3.183022e-10
x3     4 0.009675851 0.001885533 0.001543049 3.596465e-10
x4     5 0.009833890 0.001671432 0.002051581 4.016547e-09
x5     6 0.010683855 0.001794033 0.002189455 2.597264e-09
```

Note: The results here are slightly different from the estimates from the original dataset.

## Modifications on linear association between PM2.5 and stillbirth

The following codes are used to derive the subpopulation-specific linear associations between PM2.5 and stillbirth. The significance of the interaction between PM2.5 and each subpopulation indicator is examined by Wald test.

```
library(aod)
efs=c("age4","ntl3")

f="Surv(one,end)~ef:x+ns(maternal.age,3)+multiparous+ns(TMP,3)+ns(end.m,4)+ntl+strata(v2id)"
f=paste(f,"+ridge(",paste(colnames(bs),collapse=","),",theta=",theta1,")",               "+ridge(",paste(colnames(bs1),collapse=","),",theta=",theta2,")",sep="")

coef=NULL
for(ef in efs)
{
  dta$ef=dta[,ef]
  m<-coxph(as.formula(f),data=cbind(dta,bs,bs1),robust=T)
  tmp=as.data.frame(summary(m)$coef)
  id<-which(substr(rownames(tmp),start=1,stop=2)=="ef")
  L=diag(rep(1,length(id)))[-length(id),]-diag(rep(1,length(id)))[-1,]
  if(class(L)=="numeric") L=t(L)
  wt<-wald.test(m$var2[id,id],coef(m)[id],L=L)
  tmp=tmp[id,]
  tmp$grp=gsub(":x","",fixed=T,gsub("ef","",rownames(tmp)))
  tmp$ef=ef
  tmp$pval=as.numeric(wt$result$chi2["P"])
  coef=rbind(coef,tmp)
}
coef
```

```
                      coef    se(coef)         se2    Chisq DF            p
ef[0,20):x     0.007666318 0.001941548 0.002356998 15.59112  1 7.862287e-05
ef[20,30):x    0.010672523 0.001825128 0.002226728 34.19380  1 4.988766e-09
ef[30,35):x    0.010307057 0.001906409 0.002336730 29.23057  1 6.425742e-08
ef[35,Inf):x   0.015486599 0.002070263 0.002553899 55.95784  1 7.404189e-14
ef[0,0.6]:x    0.009819629 0.001860276 0.002274913 27.86347  1 1.301847e-07
ef(0.6,6.7]:x  0.011325189 0.001860066 0.002254223 37.07101  1 1.139049e-09
ef(6.7,64.4]:x 0.011274373 0.002051733 0.002481097 30.19556  1 3.906036e-08
                      grp   ef         pval
ef[0,20):x         [0,20) age4 1.553668e-05
ef[20,30):x       [20,30) age4 1.553668e-05
ef[30,35):x       [30,35) age4 1.553668e-05
ef[35,Inf):x     [35,Inf) age4 1.553668e-05
ef[0,0.6]:x       [0,0.6] ntl3 1.566427e-01
ef(0.6,6.7]:x   (0.6,6.7] ntl3 1.566427e-01
ef(6.7,64.4]:x (6.7,64.4] ntl3 1.566427e-01
```

Note:

- efs: The effect modifiers examined in this study. Here, we use nightlight and maternal age as two examples. In practice, efs should document many modifiers.

## Nonlinear association between PM2.5 and stillbirth

The following codes are to derive the exposure-response curve for the all-ages group. The nonlinear association is estimated by a set of thin-plate spline functions.

```
library(mgcv)
ERF=data.frame(end=0,x=c(0,seq(quantile(dta$x,0.01),quantile(dta$x,0.99),0.1)))
B<-gam(end~s(x,k=4)-1,data=rbind(ERF,dta[,c("end","x")]),knots=list(x=seq(quantile(dta$x,0.01),quantile(dta$x,0.99),length=4)))
Bs=model.matrix(B)
colnames(Bs)=paste("Bs",1:dim(Bs)[2],sep="")
Bs1=Bs[-c(1:dim(ERF)[1]),]
Bs=Bs[1:dim(ERF)[1],]
      
m<-coxph(as.formula(gsub("~ef:x",paste("~",paste(colnames(Bs),collapse =  "+"),sep=""),f)),data=cbind(Bs1,dta,bs,bs1),robust=T)

ERF$fit=Bs%*%coef(m)[colnames(Bs)]
ERF$fit=ERF$fit-ERF$fit[1]
vc=m$var2
colnames(vc)=names(coef(m))
rownames(vc)=names(coef(m))
ERF$se=sqrt(diag(Bs%*%vc[colnames(Bs),colnames(Bs)]%*%t(Bs)))
ERF$lo=ERF$fit-ERF$se*1.96
ERF$up=ERF$fit+ERF$se*1.96

library(ggplot2)
ggplot(data=ERF)+geom_path(aes(x=x,y=fit))+geom_ribbon(aes(x=x,ymin=lo,ymax=up),alpha=0.1)+theme_bw()+xlab(expression(PM[2.5]~(mu~g/m^3)))+ylab("Log(OR)")
```

Note: + B: A pseudo-model to generate the thin-plate splines + Bs: The spline matrix for a series of PM2.5 concentrations to generate the exposure-response curve. In the sequence, the first value is set as zero, the reference level of no effect. + Bs1: The matrix under the same rule of spline expansion to be utalized in the regression model.

## Age-specific nonlinear associations between PM2.5 and stillbirth

The following codes are to derive the age-specific exposure-response curves, the main model utalized in our risk assessments.

```
ERF=expand.grid(end=0,x=c(0,seq(quantile(dta$x,0.01),quantile(dta$x,0.99),0.1)),age4=levels(dta$age4))
B<-gam(end~s(x,k=4,by=age4)-1,data=rbind(ERF,dta[,c("end","x","age4")]),knots=list(x=seq(quantile(dta$x,0.01),quantile(dta$x,0.99),length=4)))
  
Bs=model.matrix(B)
colnames(Bs)=paste("Bs",1:dim(Bs)[2],sep="")
Bs1=Bs[-c(1:dim(ERF)[1]),]
Bs=Bs[1:dim(ERF)[1],]

m<-coxph(as.formula(gsub("~ef:x",paste("~",paste(colnames(Bs),collapse =  "+"),sep=""),f)),data=cbind(Bs1,dta,bs,bs1),robust=T)
ERF$fit=Bs%*%coef(m)[colnames(Bs)]
ERF$fit=ERF$fit
vc=m$var2
colnames(vc)=names(coef(m))
rownames(vc)=names(coef(m))
ERF$se=sqrt(diag(Bs%*%vc[colnames(Bs),colnames(Bs)]%*%t(Bs)))
library(plyr)
ERF=ddply(ERF,.(age4),summarize,x=x,se=se,fit=fit-fit[which(x==0)])
ERF$lo=ERF$fit-ERF$se*1.96
ERF$up=ERF$fit+ERF$se*1.96

ggplot(data=ERF)+geom_path(aes(x=x,y=fit,group=age4,col=age4))+geom_ribbon(aes(x=x,ymin=lo,ymax=up,fill=age4),alpha=0.1)+theme_bw()+xlab(expression(PM[2.5]~(mu~g/m^3)))+ylab("Log(OR)")
```

Note: In the above codes, we set the no-effect reference as zero. It can be set as another value, e.g., the WHO AQG level, as well.

## Datasets to reproduce the figures in main-text

### Figure 1

```
load("Figure_1.RData")
library(ggplot2)
library(ggsci)
library(bbplot)
library(gridExtra)
grid.arrange(Fig1,Fig2,Fig3,ncol=1)
```

The R objects document the data underlying the figures. The readers can look at the data using the following codes.

```
names(Fig1)
```

```
 [1] "data"        "layers"      "scales"      "mapping"     "theme"      
 [6] "coordinates" "facet"       "plot_env"    "labels"      "guides"
```

```
str(Fig1$data)
```

```
'data.frame':   48411 obs. of  51 variables:
 $ long        : num  31.3 31.2 31.1 30.9 30.7 ...
 $ lat         : num  -22.4 -22.3 -22.3 -22.3 -22.3 ...
 $ order       : int  1 2 3 4 5 6 7 8 9 10 ...
 $ hole        : logi  FALSE FALSE FALSE FALSE FALSE FALSE ...
 $ piece       : Factor w/ 133 levels "1","2","3","4",..: 1 1 1 1 1 1 1 1 1 1 ...
 $ id          : chr  "0" "0" "0" "0" ...
 $ group       : Factor w/ 675 levels "0.1","1.1","2.1",..: 1 1 1 1 1 1 1 1 1 1 ...
 $ PM25        : num  18.8 18.8 18.8 18.8 18.8 ...
 $ majorage    : Factor w/ 4 levels "age10","age20",..: 2 2 2 2 2 2 2 2 2 2 ...
 $ PM25dlt     : num  0.0339 0.0339 0.0339 0.0339 0.0339 ...
 $ AF.5.Byage  : num  0.243 0.243 0.243 0.243 0.243 ...
 $ AN.5.Byage  : num  2039 2039 2039 2039 2039 ...
 $ AF.10.Byage : num  0.162 0.162 0.162 0.162 0.162 ...
 $ AN.10.Byage : num  1361 1361 1361 1361 1361 ...
 $ AF.15.Byage : num  0.0743 0.0743 0.0743 0.0743 0.0743 ...
 $ AN.15.Byage : num  623 623 623 623 623 ...
 $ AF.25.Byage : num  1.61e-05 1.61e-05 1.61e-05 1.61e-05 1.61e-05 ...
 $ AN.25.Byage : num  0.135 0.135 0.135 0.135 0.135 ...
 $ AF.35.Byage : num  0 0 0 0 0 0 0 0 0 0 ...
 $ AN.35.Byage : num  0 0 0 0 0 0 0 0 0 0 ...
 $ AF.5.Allage : num  0.238 0.238 0.238 0.238 0.238 ...
 $ AN.5.Allage : num  2000 2000 2000 2000 2000 ...
 $ AF.10.Allage: num  0.156 0.156 0.156 0.156 0.156 ...
 $ AN.10.Allage: num  1306 1306 1306 1306 1306 ...
 $ AF.15.Allage: num  0.0755 0.0755 0.0755 0.0755 0.0755 ...
 $ AN.15.Allage: num  634 634 634 634 634 ...
 $ AF.25.Allage: num  0.00961 0.00961 0.00961 0.00961 0.00961 ...
 $ AN.25.Allage: num  80.6 80.6 80.6 80.6 80.6 ...
 $ AF.35.Allage: num  0.00102 0.00102 0.00102 0.00102 0.00102 ...
 $ AN.35.Allage: num  8.54 8.54 8.54 8.54 8.54 ...
 $ AF.5.Zhang  : num  0.128 0.128 0.128 0.128 0.128 ...
 $ AN.5.Zhang  : num  1074 1074 1074 1074 1074 ...
 $ AF.10.Zhang : num  0.0856 0.0856 0.0856 0.0856 0.0856 ...
 $ AN.10.Zhang : num  718 718 718 718 718 ...
 $ AF.15.Zhang : num  0.0428 0.0428 0.0428 0.0428 0.0428 ...
 $ AN.15.Zhang : num  359 359 359 359 359 ...
 $ AF.25.Zhang : num  0.00193 0.00193 0.00193 0.00193 0.00193 ...
 $ AN.25.Zhang : num  16.2 16.2 16.2 16.2 16.2 ...
 $ AF.35.Zhang : num  0.000108 0.000108 0.000108 0.000108 0.000108 ...
 $ AN.35.Zhang : num  0.904 0.904 0.904 0.904 0.904 ...
 $ AF.5.Xie    : num  0.176 0.176 0.176 0.176 0.176 ...
 $ AN.5.Xie    : num  1473 1473 1473 1473 1473 ...
 $ AF.10.Xie   : num  0.116 0.116 0.116 0.116 0.116 ...
 $ AN.10.Xie   : num  973 973 973 973 973 ...
 $ AF.15.Xie   : num  0.0608 0.0608 0.0608 0.0608 0.0608 ...
 $ AN.15.Xie   : num  510 510 510 510 510 ...
 $ AF.25.Xie   : num  0.0117 0.0117 0.0117 0.0117 0.0117 ...
 $ AN.25.Xie   : num  97.8 97.8 97.8 97.8 97.8 ...
 $ AF.35.Xie   : num  0.00166 0.00166 0.00166 0.00166 0.00166 ...
 $ AN.35.Xie   : num  13.9 13.9 13.9 13.9 13.9 ...
 $ N           : num  8392 8392 8392 8392 8392 ...
```

Note:

- The data documented here should only be utilized to reproduce figures. For any other purposes, please contact the corresponding author.
- PM25: the pregnancy-number-weighted concentration of PM2.5
- AF.x.Y: attributable fraction (%) of PM2.5-related stillbirths
- AN.X.Y: attributable number of PM2.5-related stillbirths
- X = 5, 10, 15, 25, or 35: the reference level of no-effect concentration
- Y = Allage, Byage, Zhang, or Xie: four alternative exposure-response curves (for details, please refer to the main-text)
- In panel (a), we don’t show the geographic locations of the surveyed samples, in order to protect confidentiality. The detailed geographic information should be obtained from the DHS website with permission.

### Figure 2

```
load("Figure_2.RData")
print(Fig)
```

```
str(Fig$data)
```

```
'data.frame':   8939 obs. of  5 variables:
 $ y  : Ord.factor w/ 7 levels "Age-specific curve: < 20 yr"<..: 1 1 1 1 1 1 1 1 1 1 ...
 $ lo : num  -0.0942 -0.0939 -0.0936 -0.0932 -0.0929 ...
 $ up : num  -0.0942 -0.0908 -0.0874 -0.084 -0.0806 ...
 $ fit: num  -0.0942 -0.0924 -0.0905 -0.0886 -0.0867 ...
 $ x  : num  0 0.1 0.2 0.3 0.4 0.5 0.6 0.7 0.8 0.9 ...
```

Note:

- The data documented here should only be utilized to reproduce figures. For any other purposes, please contact the corresponding author.
- y: Exposure-response curve
- lo, up: 95% CI boundaries
- fit: point-estimate
- x: PM2.5 concentrations

### Figure 3

```
load("Figure_3.RData")
grid.arrange(Fig1,Fig2,ncol=1)
```

```
str(Fig1$data)
```

```
'data.frame':   5964 obs. of  6 variables:
 $ r  : int  1 2 3 4 5 6 7 8 9 10 ...
 $ c  : int  1 1 1 1 1 1 1 1 1 1 ...
 $ ann: num  1 1.1 1.2 1.3 1.4 1.5 1.6 1.7 1.8 1.9 ...
 $ y  : num  0.817 72.813 117.06 102.336 92.415 ...
 $ col: Ord.factor w/ 4 levels "< 20 yr"<"20-29 yr"<..: 1 1 1 1 1 1 1 1 1 1 ...
 $ x  : num  3.4 3.03 2.95 3.08 3.04 ...
```

Note:

- The data documented here should only be utilized to reproduce figures. For any other purposes, please contact the corresponding author.
- ann: annual mean of PM2.5 concentrations in 2015
- col: age groups
- y: population
- x: percentage

### Figure 4

```
load("Figure_4.RData")
print(Fig)
```

```
str(Fig$data)
```

```
'data.frame':   125 obs. of  8 variables:
 $ r    : Ord.factor w/ 25 levels "2000"<"2001"<..: 1 2 3 4 5 6 7 8 9 10 ...
 $ x    : num  1 2 3 4 5 6 7 8 9 10 ...
 $ reg  : Ord.factor w/ 6 levels "ALL"<"Latin America & Caribbean"<..: 4 4 4 4 4 4 4 4 4 4 ...
 $ est  : num  456098 446828 439736 429838 414178 ...
 $ lo   : num  371816 359509 358526 349344 338356 ...
 $ up   : num  545351 529368 523289 507656 489904 ...
 $ width: num  1 1 1 1 1 1 1 1 1 1 ...
 $ grp  : Ord.factor w/ 2 levels "Historical trend"<..: 1 1 1 1 1 1 1 1 1 1 ...
```

Note:

- The data documented here should only be utilized to reproduce figures. For any other purposes, please contact the corresponding author.
- x & r: index and labels for the x-axis
- reg: geographic regions
- est, lo & up: estimates of the PM2.5-related stillbirths and boundaries of their 95% CI
- y: population
- grp: different types of estimates
